# Supplementary material for: Exploring the lived experience and chronic low back pain beliefs of English-speaking Punjabi and white British people: a qualitative study within the NHS
Source: BMJ Open. 2018 Feb 11;8(2):e020108. doi: 10.1136/bmjopen-2017-020108 (PMC5829944; doi:10.1136/bmjopen-2017-020108)
Supplement: Supplementary file 1 [file bmjopen-2017-020108supp001.pdf]

Supplementary File 1

Consolidated criteria for reporting qualitative studies (COREQ): 32-item checklist

**No. Item**	**Guide questions**	**Description**	**Location in manuscript (Section)**
**Domain 1: Research team and reflexivity**			
Personal Characteristics			
1. Interviewer/facilitator	Which author/s conducted the interview or focus group?	GS, CN	Methods and Results
2. Credentials	What were the researcher's credentials? E.g. PhD, MD	GS – BSc<br>CN –MSc, BSc<br>KOS – PhD, MSc PG Cert HE<br>AS – PhD, BSc<br>NH - PhD, MSc, Grad Dip Phys, PG Cert HE,	Methods
3. Occupation	What was their occupation at the time of the study?	Musculoskeletal Physiotherapists	Methods
4. Gender	Was the researcher male or female?	Male	Methods
5. Experience and training	What experience or training did the researcher have?	GS/CN undertook 3 hours of NIHR training on semi-structured interviewing	Methods
Relationship with participants			
6. Relationship established	Was a relationship established prior to study commencement?	No	Methods
7. Participant knowledge of the interviewer	What did the participants know about the researcher? e.g. personal goals, reasons for doing the research	Participants were briefed on the purpose of the study and understood that it was a research project for GS.<br>Ethical approval had been granted, participants reviewed the participant information leaflet prior to giving their written informed consent to be involved.	Methods, Acknowledgements: footnotes (ethical approval)
8. Interviewer	What characteristics were	Potential source of bias: GS is a	Methods, Strengths

characteristics	reported about the inter viewer/facilitator? e.g. Bias, assumptions, reasons and interests in the research topic	British Punjabi male and CN a White British male with 11 and 13 years musculoskeletal physiotherapist experience respectively, both with special interests in CLBP disorders	limitations and implications for future research
**Domain 2: study design**			
Theoretical framework			
9. Methodological orientation and Theory	What methodological orientation was stated to underpin the study? e.g. grounded theory, discourse analysis, ethnography, phenomenology, content analysis	Interpretive description and thematic analysis	Methods
Participant selection			
10. Sampling	How were participants selected? e.g. purposive, convenience, consecutive, snowball	Purposive sampling	Methods
11. Method of approach	How were participants approached? e.g. face-to-face, telephone, mail, email	Mail – study<br><br>Information, and a consent letter for telephone contact were posted to eligible individuals with their physiotherapy appointment letter.	Methods
12. Sample size	How many participants were in the study?	Ten	Methods and Results – Table 1
13. Non-participation	How many people refused to participate or dropped out? Reasons?	Ten potential participants that were invited for a semi-structured interview, all gave informed consent and completed the interview. There were no participants who subsequently refused to participate, withdrew consent or dropped out.	Methods
Setting			
14. Setting of data collection	Where was the data collected? e.g. home, clinic, workplace	All interviews were conducted in a quiet room in the physiotherapy department.	Methods
15. Presence of non-participants	Was anyone else present besides the participants and researchers?	No	n/a
16. Description of sample	What are the important characteristics of the sample? e.g. demographic	The sample comprised of five White British (2 males: 3 females) and five English speaking Punjabi (3 males: 2 females) people, with	Methods and Results – Table 1

	data, date	a mean age of 40 years. Data was collected between April 2014 and April 2015.	
Data collection			
17. Interview guide	Were questions, prompts, guides provided by the authors? Was it pilot tested?	Interviews were semi-structured using a topic guide (Supplementary File 3) informed by contemporary CLBP literature, and research team expertise. Further refinements were made following two pilot interviews with CLBP patients.	Methods, Supplementary File 3
18. Repeat interviews	Were repeat inter views carried out? If yes, how many?	No	n/a
19. Audio/visual recording	Did the research use audio or visual recording to collect the data?	The interviews were audio-recorded	Methods
20. Field notes	Were field notes made during and/or after the inter view or focus group?	No	n/a
21. Duration	What was the duration of the interviews or focus group?	Interviews lasted between 60-70 minutes.	Methods
22. Data saturation	Was data saturation discussed?	Recruitment continued until data saturation was met for the purpose of the study.	Methods
23. Transcripts returned	Were transcripts returned to participants for comment and/or correction?	No	n/a
**Domain 3: analysis and findings**			
Data analysis			
24. Number of data coders	How many data coders coded the data?	GS, CN and AS independently assessed the accuracy and completeness of all the transcripts	Methods
25. Description of the coding tree	Did authors provide a description of the coding tree?	Provided in Table 3: The thematic development illustrating key stages when themes were changed.	Results – Table 3
26. Derivation of themes	Were themes identified in advance or derived from the data?	GS, CN and AS ensured themes related to the thematic development and emerging	Methods

		themes	
27. Software	What software, if applicable, was used to manage the data?	Microsoft word	n/a
28. Participant checking	Did participants provide feedback on the findings?	Member checking was not conducted to validate interview transcripts due to time and funding issues.	Strengths limitations and implications for future research
Reporting			
29. Quotations presented	Were participant quotations presented to illustrate the themes/findings? Was each quotation identified? e.g. participant number	Yes, specific comments were supported with direct quotes derived from each participant. Quotations were identified using anonymised participant codes.	Results and Supplementary file 5
30. Data and findings consistent	Was there consistency between the data presented and the findings?	Yes	n/a
31. Clarity of major themes	Were major themes clearly presented in the findings?	Yes	n/a
32. Clarity of minor themes	Is there a description of diverse cases or discussion of minor themes?	Yes	Discussion
