# Supplementary material for: Exploring the lived experience and chronic low back pain beliefs of English-speaking Punjabi and white British people: a qualitative study within the NHS
Source: BMJ Open. 2018 Feb 11;8(2):e020108. doi: 10.1136/bmjopen-2017-020108 (PMC5829944; doi:10.1136/bmjopen-2017-020108)
Supplement: Supplementary file 2 [file bmjopen-2017-020108supp002.pdf]

Supplementary File 2

1st Topic guide

Notes: The topic guide will be a flexible tool which is open to revision as new areas of interest arise during the process of data collection. In order to limit the length of the interviews, it is not essential that each interview should include every line of questioning as detailed below. Depth of exploration of fruitful areas of discussion is more important than complete coverage of all areas in every interview.

Introduction to the interview:

The interviewer will reassure the person being interviewed that:

- The research study is interested in finding out about their beliefs and experiences in living with CLBP
- There is no intention of 'testing' the interviewees about how well they are managing their CLBP.
- That they are free to say as much or as little as they wish in response to any line of questioning.
- That the content of the interview will not be divulged to their Doctor or Physiotherapist.

Background information:

In order to be able to describe the sample of patients who contribute to the study, the interviewer will invite the person being interviewed to provide information about:

- Ethnicity - participant to define their ethnicity
- Age
- Occupation
- When their LBP first started
- Previous treatment

Interview guide with prompts:

Opening

A. History of illness and health seeking behaviour

1) When did it start to impact on your daily life?

2) Who have seen regarding your CLBP problem?

Probe:

- Have you had any Investigations?

Prompts i.e MRI/ x-ray

- What did they show?
- What treatment (s) have you had in the past for your LBP?
- How **helpful** were these **treatments?**

B. Coping strategies

3) How do you cope with your CLBP at present?

Probe:

- What strategies do you have to manage your CLBP?
- What do you do when you have a flare up of pain?
- How do those around you cope/help (i.e. family or friends)?
- Does anyone in your family suffer with CLBP? How do they cope?

  How well do you think you manage your CLBP?

C. Cause of condition

4) What have you been told about the **cause** of your CLBP?
   - How did this make you feel?

5) What are your thoughts about the cause of your CLBP? Why do you think this?

D. CLBP beliefs

6) Have you come across any other views or beliefs related to the cause of CLBP amongst people you know?

Probe:

- Where do you think they come from?
- Is that what you think yourself?

E. Alternative treatments

7) Some people use alternative treatments e.g. acupuncture/reiki/reflexology for LBP,
8) What are your thoughts on these types of treatment?
9) Is this something you have used or would consider using?
   a. Why?
   b. Why not?

Probe:

- Benefits
- Disadvantages

F. CLBP and physical activity

10) What are your thoughts about people with back pain doing **physical tasks/jobs**?

Probe:

- Is bending/ lifting safe to do?
- Is there anything you avoid doing because of your CLBP?

G. Living with CLBP

11) What do you think is the effect of CLBP on your mood?

12) How much of your focus and attention does your CLBP take?

13) What do you feel will be the **best way to treat** your CLBP from now?

Probe:

- What type of treatment?

14) How do you see the **future** with regards to your CLBP?

Probe:

- How hopeful are you about the future?
- Do you feel your LBP will get better?

Ending questions

15) Is there anything else that you would like to say that we may not have covered already or discussed?

Thank you for co-operation and for taking the time to answer all the questions.

After 3rd interview Revised Topic guide
Background information:

In order to be able to describe the sample of patients who contribute to the study, the interviewer will invite the person being interviewed to provide information about:

- Ethnicity - participant to define their ethnicity
- Age

- Occupation
- PMH
- DH
- Activity levels /exercise
- Sleep hygiene

Interview guide with prompts:

Opening

A. History of illness and health seeking behaviour

Could you tell me the story about your LBP?

Probe (if it is not clear from the narrative question)

- When it started, how it started?
- When did it start to impact on your life?
- How did this affect you?

B. CLBP experiences and beliefs

Childhood/Family history of LBP/pain

Can you recall any early life experiences of LBP /pain?

- What was your experience?
- Were you told anything about it at the time? By whom?
- How did you cope with it?
- How did your family/friends react /respond towards your LBP/pain?
- What was the attitude of your parents towards your LBP/pain?

While you were growing up, did your parents/family member(s) suffer with LBP/pain?

- What was their experience?
- How did it impact on them?
- What was the cause of their pain?
- How did they try to cope with it?
- Did it have any impact on you and your family?
- Do you feel these experiences of pain have affected the way you think about your LBP?

Who have you been to see regarding your LBP?

- What was your experience?
- Did you have any examinations or tests? i.e. MRI/ x-ray
- What did they show?
- How was this explained to you?
- Was the language used simple, or did it include medical terms?
- What was your understanding of this?

- How did this make you feel?

What have you been told about the cause of your LBP?

- By whom?
- How was this explained to you?
- Was the language used simple, or did it include medical terms?
- What was your understanding of this?
- How did this make you feel?

What are your thoughts about the cause of your LBP?

- What makes you think this?
- What things have influenced your thoughts?
    - Family, culture, pain experiences, interactions with HCPs?

What do you feel people around you think causes LBP?

- Where do you think these ideas come from?

What treatment(s) have you had in the past for your LBP?

- What was your experience of these treatments?
- Did they match what you thought would be best for your LBP? If not, why not?
- Did these treatments influence your:
    - Control over your LBP?
    - Ability to do things you wanted to do?
    - Confidence regarding your back?

How do you cope with your LBP now?

- If you had a flare up, how would you manage it? Why?
- How would you manage an acute ankle or knee sprain?
- Would this be the same or different to a LBP flare up? If not, why?
- How do those around you respond or react to you regarding your LBP (i.e. family or friends)?

C. <u>HCP relationships</u>

How do you feel HCP's have interacted with you regarding your LBP?

- What was your experience of the time they spent?
- How well did they listening to you?
- Did they build your confidence?

Do you feel your cultural background influences your relationship with HCP's and the way you have been treated?

- What are your experiences?
- Have you experienced any differences or inequalities due to your cultural background?

D. Views on exercise and physical activity

What are your thoughts about people with LBP doing physical exercise?

- Why?
- Is there anything you should avoid doing because of your LBP? Why?
- What are views on people with LBP bending/ lifting? Why?

E. Alternative treatments

Many people use alternative treatments e.g. acupuncture/reiki/reflexology for LBP

What are your thoughts on these types of treatments? Why?

What do you think are the best treatments for LBP? Why?

F. Living with CLBP

In your experience has LBP impacted on your:

- Health? Activity? Social life? Work? General health? Comorbidities? Other pain areas? Sleep? Mood?
- How?
- How much do you think about your back? Why?

How do you picture yourself in 5 years with regards to your CLBP?

- How hopeful are you about the future?
- Do you feel your LBP will get better? Why?

Ending questions

Is there anything else that you would like to say that we may not have covered already or discussed?

Thank you for co-operation and for taking the time to answer all the questions.
