# Supplementary material for: Exploring the lived experience and chronic low back pain beliefs of English-speaking Punjabi and white British people: a qualitative study within the NHS
Source: BMJ Open. 2018 Feb 11;8(2):e020108. doi: 10.1136/bmjopen-2017-020108 (PMC5829944; doi:10.1136/bmjopen-2017-020108)
Supplement: Supplementary file 3 [file bmjopen-2017-020108supp003.pdf]

Supplementary File 3

Table 3: The thematic development illustrating key stages

	Thematic development stage 1		Thematic development stage 2	Thematic development stage 3	Final theme stage 4
**Theme 1**	Back pain beliefs	Revised Topic guide	Back pain beliefs	Biomedical Back pain beliefs	Biomedical Back pain beliefs
**Theme 2**	Coping with CLBP		Coping with CLBP	Coping with CLBP	Coping with CLBP
**Theme 3**	Psychosocial, dimensions of pain		Impact of CLBP	Psychological, emotional and social dimensions of CLBP	The psychological and emotional dimensions of living with CLBP
**Theme 4**	Cultural impact of CLBP		Cultural impact of CLBP	Cultural impact of CLBP	The social and cultural-religious impact of CLBP
**Theme 5**	HCP interactions		HCP interactions, management experience and future expectations	HCP interactions, management experience and future expectations	Reflecting on HCP interactions, management experience and expectations of future management

CLBP = Chronic low back pain
HCP = Health care practitioner
