# Supplementary material for: Exploring the lived experience and chronic low back pain beliefs of English-speaking Punjabi and white British people: a qualitative study within the NHS
Source: BMJ Open. 2018 Feb 11;8(2):e020108. doi: 10.1136/bmjopen-2017-020108 (PMC5829944; doi:10.1136/bmjopen-2017-020108)
Supplement: Supplementary file 4 [file bmjopen-2017-020108supp004.pdf]

Supplementary File 4

Audit Trail example

Interview transcript exerts and comments

The GS's comments on interviews are illustrated below,

<u>Transcript S1, lines 1019-1042</u>

I: Right, that's interesting. So with regards to bending and lifting, what are your thoughts on that?

P: I make sure that…. when I am lifting something or I have to bend down, I make absolutely sure that I am bending my knees and not just tipping over or whatever

I: Ok

P: You know, your manual handling and all that comes in….so you start thinking, the last thing I want to do is injure myself further or cause any more discomfort

I: Ok

P: So you are bending your knees and making sure you are lifting it*….**<u>GS's thoughts</u>** Impact of manual handling training/Impact of pain on function – bending/lifting - Adopted cautious/protective behvoiur to bending /lifting -Psychological impact vigilance/catstrophising*

I: And how long have you been doing that for?

P: Well, years now

I: So before your back started hurting?

P: O yeah

I: Then has that changed at all since your back?

P: No….I think a bit more….conscious. Consciously, it's almost like if you have….when I say you…, if I have a task that it would probably involve lifting a box or whatever, I will now consciously think, make sure you bend your knees and you keep your back straight if you're lifting something or whatever. Whereas previously you think you are fine, it doesn't matter, nothing is going to happen so*…. **<u>GS's thoughts</u>** Psychological impact of pain – hypervigilance/ catastrophising with lifting having - Adopted manual handling behavoiur to protect back from future pain - Increased threat to back*

I: So your thought process around your lifting has changed?

P: It has changed, yes. It will change

I: Ok. So what thoughts are going through your mind then when you see something heavy on the floor?

P: Making sure that you don't end up causing more damage ***<u>GS's thoughts</u>** Coping startgeies- Atuuidtde to pain -Biomedical belief -Protective behavoiur/Caution*
