# Supplementary material for: Exploring the lived experience and chronic low back pain beliefs of English-speaking Punjabi and white British people: a qualitative study within the NHS
Source: BMJ Open. 2018 Feb 11;8(2):e020108. doi: 10.1136/bmjopen-2017-020108 (PMC5829944; doi:10.1136/bmjopen-2017-020108)
Supplement: Supplementary file 5 [file bmjopen-2017-020108supp005.pdf]

Supplementary File 5

Table: Theme 1 examples

Theme	Subtheme	Code	Example quotes from participants
**Biomedical back pain beliefs**	*Subtheme 1.1: Cause of CLBP attributed to physical (structural/anatomical) factors*	Back strain with bending	"I went pick a pair of socks up from the floor so and as I went to pick it up as I was getting back up there was just like this crack sounding noise and erm I think that was my first experience of it." [S10]
		Back strain with lifting	"It was definitely related, there was a timber and I went to pick it up and I felt my back hurt" [S2]
		Specific traumatic event	"I was involved in a car accident where my car, at a roundabout junction, was hit from the back and I think because of the angle I was standing with my neck turned, the impact was quite severe" [S1 PM MD MPR]. "I had a complicated pregnancy in 1995. Ahh, with my son, it was a very difficult birth; really prolonged labour and ammm significant problems during the actual birth. That was quite a shock, needless to say. I didn't have any epidural or anything like that when I had him so my body went in to shock I think with the experience ….I started to get it…." [S4 WBF MD MPR]
		Biomedical self diagnosis	"It could be a trapped nerve, a slipped disc" [S1 PM MD MPR] "…I suppose I'd always think it gets to the point where they cant actually find anything wrong so…its wear and tear because of your age, because there's 50 odd years of…and its almost something which I accept. You almost expect it because you see old people who are struggling with joints and this sort of thing and you think well yeah but for 80 odd years those knees have worked so I can understand a bit of it" [S8 WM LD LPR]
		Biomedical Back Pain beliefs in the Family and cultural community	"My mother in law just says its lifting and stuff" [S3 WBF MD MPR]. "They think that by lifting heavy weights or something or moving something heavy that that does their back in"[S7 PM MD MPR]
	Subtheme 1.2: Recalling HCP biomedical diagnosis and the biomedical beliefs	Biomedical diagnosis	"They thought it was sciatica"[S5 PF HD HPR] "The doctors have told me that I have got sacralisation"[S2 WBM MD HPR]
		Biomedical diagnosis via MRI	[Recalling MRI diagnosis] "It ermm showed like a bulging…ermm at the lower…spine but it was down to my age" [S9 WBF MD HPR] "I had an M.R.I. scan which was showing that I had got wear and tear on the vertebrates and that L4 and L5 were closing up" [S3 WBF MD

	adopted	Manual handling influence: protect spine with lifting and bending<br><br>Nurse occupational belief	MPR].<br>" I suppose if you lift incorrectly it will cause back pain…. I mean we have all had it drummed in to us, how to bend our knees and how to do all that malarkey" [S2 WBM MD HPR]<br>"Well that's what you get on all these health and safety things at work and lifting weights, they, they reckon you're supposed to bend at the knees, keep your back straight and stand with your legs" [S8 WM LD LPR]<br>"I was a nurse for many many years on a very heavy surgical ward and I wonder if maybe I have damaged it while nursing, you kind of associate nursing with back pain, well I do anyway."[S2 WBM MD HPR]
	Subtheme 1.3: Vulnerability of the spine	Central to function<br><br>Can't see the back<br><br>Feeling weak and cautious<br><br>Precious<br><br>Needs urgent attention<br><br>Fear avoidant beliefs	"I rely on it well most people rely on their back"[S6 PM LD LPR]<br>"Your spine in general is…central to you ….. I think you're reminded of it more each day" [S8 WM LD LPR]<br>"Because your back's...behind you, you can't see what's going on inside it or outside it."[S10 PF HD HPR]<br>"It's always been slightly weaker well felt slightly weaker well it felt slightly weaker so I was just a bit more cautious of it"[S6 PM LD LPR]<br>"I've always had a bit of a weakness in my back for quite a few years" [S9 WBF MD HPR]<br>"My back seems a bit more precious" [S6 PM LD LPR]<br>"I feel like my back's more precious rather than my wrist or ankle because it is a vital piece" [S5 PF HD HPR]<br>"So if you know there is a problem, go and fix it….the thing is if you don't fix it…then the impact of it…so the old English saying 'a stitch in time saves nine" [S1 PM MD MPR].<br>"Do try and get it sorted out at the most earliest point. Don't leave it" [S3 WBF MD MPR].<br>"Obviously not lifting really heavy stuff"[S5 PF HD HPR]<br>"…Only pick up something that you feel you could pick up, it's not too heavy." [S10 PF HD HPR]
	Subtheme 1.4: Future outlook	Positivity<br><br>Pessimism	"I even think now that I will wake up and it will all go away and it maybe will." [S2 WBM MD HPR]<br>"Well hopefully in a little less pain than I am in right now" [S3 WBF MD MPR]<br>"I think it's always going to hurt me and it's always going to cause me pain" [S5 PF HD HPR]<br>"When I look into the future I just think how am I gonna cope with a child when I can't yknow

		Uncertainty<br><br>Religious beliefs interlinked with outlook on pain	carry heavy things" [S10 PF HD HPR)<br>"I can only play it by ear" [S8 WM LD LPR]<br>"But I'm not sure….having lived with it for this long, I'm not sure" [S3 WBF MD MPR]<br>[Referring to finger amputation] "As I would say, someone up there wanted them so they were gone …..my sin plate clean on that day and we start again sort of thing" [S1 PM MD MPR]

Table: Theme 2 examples

Theme	Subtheme	Code	Quote from participants
**Coping with CLBP**	*Subtheme 2.1: Active coping strategies*	Self-searching for knowledge and understanding<br><br>Learning from pain experience<br><br>Confronting pain and battling on<br><br>Acceptance and adapting to pain, due to a lack of understanding, ineffective interventions and pain duration	"Ammm, it was just something off the internet that suggested you stretch and what not…"[S3 WBF MD MPR]<br>"You have got to look at why it is hurting and you can ask yourself that question. Don't expect anyone else to have all the answers" [S4 WBF MD MPR]<br>"With me neck it certainly helps being..active, being yknow rather than sitting staring at a screen" [S8 WM LD LPR]<br>"All I was bothered about was getting things better for him (disabled child) because he literally couldn't do anything" [S4 WBF MD MPR]<br>"I just managed it and got on with it..I tend to be quite stubborn" [S3 WBF MD MPR]<br>"I have kind of giving up looking for an answer. I just think whatever happens I have just got to deal with it" [S5 PF HD HPR]<br>"Put up with it because basically nothing seemed to work" [S3 WBF MD MPR]<br>"It became normal to me because I've had that for so long. It got normal for me to me feel little niggles in my back"[S6 PM LD LPR]
	Subtheme 2.2: Reliance on HCP's and biomedical interventions	Unrelenting search for understanding answers<br><br>Explanation & reassurance via investigation	"I wonder what it is and for the children ….. I would like to know" [S3 WBF MD MPR]<br>"I'm looking for answers but I want those answers. I am a dog with a bone" [S4 WBF MD MPR]<br>"Well I wanted a second scan" [S3 WBF MD MPR]<br>"An X-ray, MRI scan or anything just so I could have some reassurance that it's nothing major" [S10 PF HD HPR]

		<u>Reliance on medication</u><br><br><br><br><br><br><u>Punjabi reliance on Biomedical Quick fix</u>	"I basically lived on painkillers ever since" [S3 WBF MD MPR]<br>"Well if it does get severe to the point where I can actually feel it…… That's the day I would probably go home and have a couple of paracetamol or something" [S1 PM MD MPR]<br>"Manipulation yeah …… I just wanted to get the back fixed"[S7 PM MD MPR]<br>" I have a massage every 4-6 weeks. Am… and acupuncture" [S5 PF HD HPR]<br>"I have had new chairs…..I got the one with the lumbar support and an incline" [S1 PM MD MPR]
	Subtheme 2.3: Protective and avoidance coping strategies	<u>Adopted caution, protect spine</u><br><br><br><br><br><br><br><br><br><br><br><br><br><br><br><br><br><br><u>Avoidance behaviour: exercise, physical task</u>	"If you keep bending at the back all the time. The back takes the strain for everything. If you're bending at the knee to access what it is you need to get it takes the pressure out of your back from a moving and handling perspective" [S4 WBF MD MPR]<br>"I can stay in the office and look after my back …… whereas previously I might think, o well I might go for a walk around the building ……I wouldn't be doing that because it wouldn't be enjoyable and it would hurt" [S2 WBM MD HPR]<br>"Subconsciously you know that you should move a certain way to avoid injury. You know that, well I do because it is part of how I live yeah" [S4 WBF MD MPR]<br>"I can't do this my back hurts, or I can't do that my back hurts" [S10 PF HD HPR]<br>"Yeah I have stopped swimming" [S4 WBF MD MPR]<br>"I used to be very active, I just don't do any exercise really at the minute" [S9 WBF MD HPR]<br>"If I bend over, that's how my back went, and it's more than once, it's twice now, anytime I go to bend down now I think maybe it's going to go again or I shouldn't be doing this" [S6 PM LD LPR]<br>"I don't do any heavy lifting" [S2 WBM MD HPR]<br>"On my drive now I've got a load of bricks and if I need to lift them up ….. ten years ago I wouldn't of thought nothing about it I would of picked them ….. whereas now maybe.... I think really haven't got it in me to lift them ….. I feel a bit weaker so it's more, now it's become if I can avoid it I avoid it . I just think why take the risk on that"[S6 PM LD LPR]
	Subtheme 2.4: Coping	<u>Punjabis passive to active</u>	"I felt like I was doing more instead of sitting in my bed all day" [S5 PF HD HPR]

	transition	coping style	"Over time I tried to not spend too much time lying down and just tried to keep myself active as possible …… just try to keep things moving" [S7 PM MD MPR]<br>"I've had to like buy books and stuff myself and do research on the internet myself to ermm be able to look into these things more" [S10 PF HD HPR]
	Subtheme 2.5: Health benefits of an active lifestyle in Punjabis	Psychological, emotional effect	"I try and do other things now to…like maybe go to the cinema and like sit in the big seats and like try and make myself a bit comfortable and like..because I've had it for so long I've gotten used to like erm making plans with my back pain so erm I'm not as depressed as I would've been maybe 5-10 years ago" [S10 PF HD HPR]

Table: Theme 3 examples

**Theme**	**Subtheme**	**Code**	**Quote from participants**
**The psychological and emotional impact of CLBP**	*Subtheme 3.1: Psychological and emotional consequences*	Depressed mood	"I wake up grumpy. I mean obviously for a person who has been able to sleep 7,8,9 hours when you are constantly waking up….even the small break in a sleep" [S1 PM MD MPR]<br>"I've never felt as low as I have done" [S9 WBF MD HPR]<br>"The injections didn't work. I kind of felt low"[S7 PM MD MPR]<br>"Sometimes I still feel really bad, really low mood because I can't do what I used "[S5 PF HD HPR]<br>"I am awful. I am moody a lot of the time…. it does make me grumpy" [S3 WBF MD MPR]
		Hopelessness	"Not much I can do about it is basically what the guy said" [S4 WBF MD MPR]<br>"There was no other route to go down" [S3 WBF MD MPR]<br>"I went and saw a chiropractor… he gave an x-ray looking for back symptoms …they said it didn't show anything, there was nothing they could do" [S7 PM MD MPR]
		Frustration	"I had an MRI scan and that's when they done and my spine was fine I was just annoyed that there was no answers" [S5 PF HD HPR]<br>"It's a ball ache and it's getting on my

			nerves" [S4 WBF MD MPR]
			"It's so frustrating where, when…sighs, yknow things like, picking up my sewing machine I just think well what's up with me?! It's ridiculous" [S9 WBF MD HPR]
		Catastrophising thoughts	"You wonder, what if? What if you can't carry on at the same level that you are" [S1 PM MD MPR] "It just makes you think is this the start of something bigger for me?"[S6 PM LD LPR]
		Uncertainty	"They couldn't really give me an explanation really I was thinking then oh what's caused my problem now"[S7 PM MD MPR] "Apprehensive, apprehensive about all those things that I have just spoken about. About what would the impact be in later life…..ahh, that's about it at the moment" [S1 PM MD MPR] "But I'm not sure….having lived with it for this long, I'm not sure" [S3 WBF MD MPR] "It's not improving, it's getting probably a bit more……regular" [S1 PM MD MPR]
		Lack of control over pain	"I'm just getting to the stage where I'm thinking on the days where it gets really really bad that I'm gone beyond the need of self-help" [S4 WBF MD MPR]
	Subtheme 3.2: Catastrophic nature of pain flare-ups	Catastrophic description: impact on body and mind	"I just…I got up and I had…I got up on the Monday morning ermmm and I felt like a cripple…I felt like I was folding it half, it was hard work to stand up straight"[S8 WBM LD LPR] "It's horrible. It's the worst pain. It makes you cry it's that bad. the pain is ermmm you just can't, you just feel like you've lost control. You can't do anything for yourself you can't like… you can't even go to yknow the loo because you're taking so many steps to get there and every step you take you're like ow, ow, ow and it's that acute ermm it's just horrible. You just…I think you just kind of lose yourself and that's when you start getting depressed like "oh god it's happened again" [S10 PF HD HPR]
	Subtheme 3.3: Threat to self-identity	Threat to self	"I suppose it's hindering my life" [S3 WBF MD MPR] "Obviously it's restricted what I can

			and can't do" [S2 WBM MD HPR]
		Disrupting personal care/daily function	"It's a small thing like bending over the sink to brush your teeth" [S4 WBF MD MPR]<br>"I got out of bed …it took me quite a while to sort of straighten up" [S8 WM LD LPR]<br>"My socks, my shoes, tying my shoes up, I can't bend down" [S9 WBF MD HPR]
		Dependency on family	"It's embarrassing sometimes and degrading because he ..has to help me put my underwear on because I cant bend down to even put my knickers on" [S9 WBF MD HPR]
		Disrupting house chores	"I get the hoover out and hoover, within 10 minutes I'm absolutely shattered and it's hurting my back" [S9 WBF MD HPR]<br>"Mowing the lawn... It's that pushing forward movement I mean it's a push mower.. I mean it takes us about an hour to mow our lawn. And I know that if I sit down after doing that, that I will be in trouble" [S3 WBF MD MPR]<br>"I can't stand at a sink for more than 10 minutes without pain." [S10 PF HD HPR]
		Barrier to recreational activities	"A fair weather golfer…. by the time I played a few holes. I could feel that there was additional pressure on the back" [S1 PM MD MPR]<br>"I can't ride my bike because it's in the leaning forward position and I'll have only gone half a mile but it causes pain in my back" [S3 WBF MD MPR]
		Sleep disruption	"Over the last year it is a genuine struggle if I woke up in the night I struggle to get back to sleep" [S4 WBF MD MPR]<br>"It's okay during the day but at night I'm really suffering"[S5 PF HD HPR]<br>"I am constantly tossing and turning ...."[S1 PM MD MPR]<br>"I sleep on my side but then….i start to ache in my hips and then I have to change sides …. I do wake up because of it and then it takes me about 20 minutes to go back to sleep" [S10 PF HD HPR].

Table: Theme 4 examples

Theme	Subtheme	Code	Quote from participants
**The social and cultural-religious impact of CLBP**	*Subtheme 4.1: Threat to family/friend relationships and social life*	Disrupting family roles/social activity	"If my back goes I can't do nothing, I wouldn't be able to do any housework or…ermmm no bending certainly no bending" [S10 PF HD HPR]<br>"If I'm restricted so is she [wife] in respect of doing things and going out and stuff…."[S2 WBM MD HPR]<br>"Even when I'm driving when we go over a speed bump or a pothole the intense pain that shoots up my back is amazing" [S3 WBF MD MPR]
		Elicit negative family emotions	"When I'm down everybody else feels down in the family" [S9 WBF MD HPR]
		Social disruption, isolation	"You have to stand up in a bar or evening and that just is so painful……., so I tend not to be social" [S10 PF HD HPR].
	Subtheme 4.2: Work impact	Disrupting work	"The pain constraints on work"[S1 PM MD MPR].<br>"I've also had to raise it with work because of the amount of time I sit by the computer and they've had to get me a special chair" [S10 PF HD HPR]
		Burdening colleagues	"While my job is actually fully sitting down, any activities where they have asked me to go and do outreach sessions, I have had to restrict them or send other people, which again isn't ideal but other people can deputise as necessary" [S2 WBM MD HPR]<br>"I didn't drive.,,.,,I went into the office a few times but then got a lift into the office from colleagues" [S7 PM MD MPR]
		Sickness absence	"I think I only might have had a few days off in the whole time I was there for the pain" [S3 WBF MD MPR]<br>"I'm off work I've had so much time off work"[S9 WBF MD HPR]
		Financial pressures	"I'm in the building trade without a back I can't earn no money. So that goes through your mind as well as that you're not going to get no sick pay or anything" [S6 PM LD LPR]<br>"Still go to work ermmm…because I've got still got bills to pay and I've still for me daughter to support through university and so on and so forth so there's that pressure" [S8 WM

		Changing role	LD LPR]<br>"Initially I suppose I thought…might as well have to look for a different type of job because of the….the physical aspect if you like" [S8 WBM LD LPR]
	Subtheme 4.3: The impact of CLBP on cultural and religious wellbeing	Religious rituals: disrupted sitting to meditate /secluding self<br><br>Disrupting female role within the home/burdening others	"I couldn't do like sit on the floor if it was too hard ..if I sat on the floor for a certain amount of time, then got up, started moving around, my legs would get pins and needles and would reach my back" [S7 PM MD MPR]<br>" [Referring to sitting in the temple] I'll just go when it's quiet yknow, ermmm do my praying and then come back out and then just go downstairs where there's chairs or.. I can just hang around somewhere else and that kind of…I feel secluded sometimes because yknow people tend to like wanna sit upstairs and I have to like go just downstairs" [S10 PF HD HPR]<br>"Because of the pain yknow like I can't help around the house as much as I would like" [S10 PF HD HPR]
	Subtheme 4.4: The response of family, friends and wider community to CLBP	Family: varying response/ support<br><br>Punjabi community: Lack of empathy/ understanding<br><br>Punjabi community: Stigmatized	"My brother……, he was just getting more and more annoyed every time because there was no answers"[S5 PF HD HPR]<br>"People were badgering me, yknow its not getting any better you ought to go …..and see"[S8 WM LD LPR]<br>"Yeah and whenever I did anything they'd say oh watch your back, you'd be carefully about, you don't want that happening again" [S9 WBF MD HPR]<br>"Everyone thinks like "oh her back her back, she just uses that as an excuse now…" [S10 PF HD HPR]<br>"Unfortunately there is always this advice from the community that oh I had a similar thing…no you didn't because you have no idea what the cause of my problem is…"[S1 PM MD MPR]<br>"I believe they're like the doctors that I've experienced. They always assume the worst first yknow" [S10 PF HD HPR]<br>"Oh there comes a fat cow, she's got back, yknow she just can't get up, or oh let's make room for her she's like she can't stand up or just that kind of response. It's not about pain it's about…oh she's too big, she can't do

			it cus she's too big"[S10 PF HD HPR]
	Subtheme 4.5: Recalling family experiences of CLBP	<u>Varying impact of CLBP/coping strategies</u><br><br><br><br><br><br><br><br><br><br><br><br><br><br><br><br><br><br><br><u>Varying HCP interactions</u>	"He [Brother] has medication to deal with that on a daily basis"[S4 WBF MD MPR]<br>"He [Dad] felt like a bit of self-dignity went because he was always self-dependent and independent, always doing his own thing"[S5 PF HD HPR]<br>"He [referring to husbands back pain] could sit but he couldn't stand and he couldn't walk…he could just barely walk from the edge of his room to the other side… to get to the kitchen and he would be bent over and he would be crippled". [S3 WBF MD MPR]<br>"He [Dad] has not let it really impact him to be honest" because he still goes to the gym, he still lift weights, just does everything. Before every now and again his back hurts a little bit. He does what he does it he hasn't changed anything"[S6 PM LD LPR]<br>"Well my mum used to have back pain and she always used to say 'oh doctors don't do anything they say they just can't do anything with it'"[S10 PF HD HPR]<br>"My dad suffered from back pain. He had a slipped disk. They said he would never walk again"[S5 PF HD HPR]<br>"She [Husbands physio] explained things; she showed us…..she showed us a back. What you should do with it, what you can do… she gave him exercises to do and how to manage it in the future better" [S3 WBF MD MPR]

Table: Theme 5 examples

Theme	Subtheme	Code	Quote from participants
**Reflecting on HCP interactions, management experience and expectations of future management**	*Subtheme 5.1: Varying quality of therapeutic alliance*	<u>Strong therapeutic alliance</u>	"I felt that it was explained pretty much…completely if you like" [S2 WBM MD HPR]<br>"I left that consultation knowing that I can physically do more than what I thought I could do"[S6 PM LD LPR]<br>"She helped me and showed me how to do it"[S5 PF HD HPR]<br>"I felt they were a lot more like approachable, they were a lot more

		Weak therapeutic alliance	understanding…..I was listened to, that was the biggest thing" [S10 PF HD HPR]<br>"I'm doing exercises, …. which I've been told to do"[S9 WBF MD HPR]<br>"My confidence was improving"[S5 PF HD HPR]<br>"[Referring to chiropractic] Why is that treatment not available on the NHS?" [S10 PF HD HPR]<br>"I get the feeling sometimes that it's maybe a textbook thing to treat back pain" [S4 WBF MD MPR]<br>"I think probably like my GP didn't take me seriously…. I don't think anybody like has the time" [S10 PF HD HPR].<br>"I don't think people understand the severity of the situation"[S4 WBF MD MPR]<br>"With the chiropractor there wasn't much listening ,with the back specialists there was to an extent but it wasn't too involved" [S6 PM LD LPR]<br>"I went back 5 times in total……………..So I kept going back until I got more or less what I had hoped for" [S2 WBM MD HPR]<br>"I got the impression from the Dr that when I went to see the back specialist they would do at least an ultrasound and she didn't" [S8 WM LD LPR]<br>"I was expecting a diagnosis. …. I want him to tell me" [S3 WBF MD MPR]<br>"Yeah, expected no more flare ups ….. within a matter of weeks, 2 weeks if that, I was just sitting down and had another flare up" [S7 PM MD MPR]<br>"She hadn't really done anything to my back, she hadn't really done the tests or anything"[S7 PM MD MPR]<br>"I think I was expecting to be….to kind of have something like that done to me rather than just saying, 'ok here are the exercises, do them at home" [S5 PF HD HPR]<br>"No sort of a aftercare at all you know put an ice pack on your back that's it "[S6 PM LD LPR]
	Subtheme 5.2: Interpreting the HCP explanation	Adopt caution, Protect spine<br><br><br><br><br>Diagnostic	"[Told by HCP] Be more careful so when you get into bed, don't just jump in"[S7 PM MD MPR]<br>"Just be careful of what I do, just be careful of how I bend, Dr's and physio" [S9 WBF MD HPR]<br>"It could be your movements, it could be

		uncertainty	your muscles, it could be disc, it could be .. anything. Ermmm but obviously…I don't know what's causing my back pain..” [S10 PF HD HPR]<br>“Mixed messages …. oh it could be your stomach, they told me that wear and tear is on my hip and that's causing pain in…. my back” [S7 PM MD MPR]
	Subtheme 5.3: Appraising interventions and ability to control CLBP	Ineffective medication, acupuncture<br><br>Short lasting manual therapies<br><br>Variable impact of injection and exercise therapies	“Some of the pain killers made no difference” [S2 WBM MD HPR]<br>“I went through the acupuncture stage and that didn't work” [S3 WBF MD MPR]<br>“I did go to see a chiropractor as well ermm because at one stage I was just like oh I can't take this anymore…. I had about 8 treatments?......I probably had about 2 or 3 treatments in one week … as soon as it stopped ermm probably about….the first 2 weeks I would say the pain was still there” [S10 PF HD HPR]<br>“They gave me some injections into my facet joints and they didn't help that much... they gave me some more injections but this time they were deeper. So since that time, I've not had any flareups as what they were before but I've had constant pain” [S7 PM MD MPR]<br>“Because I'm exercising its obviously building my muscles and whether that's causing me more pain”[S5 PF HD HPR]<br>“I went to a yoga class three weeks ago and it about killed me but I love it, the next day although there was a dull ache in my back it felt wonderful. I felt like I had really stretched it out” [S4 WBF MD MPR]<br>“My flexibility has increased”[S6 PM LD LPR]<br>“The pain goes away if I do stretching, it goes away and like arching your back over and putting your hands on your buttocks and arching back” [S3 WBF MD MPR]<br>“Exercises … I do them everyday, every morning or night… the underlying problems still there its not going away, its not getting any better” [S9 WBF MD HPR]<br>“When I was walking or running or whatever I was doing the pain was worse than it was before” [S10 PF HD HPR]<br>“My back pain got worse…it was like exercises everyday to do like my back and twisting it” [S7 PM MD MPR]

	Subtheme 5.4: Expectations of future management	Individualised management	"Almost take it case by case, person by person and work out what's best for that person"[S8 WM LD LPR]
		Mind body approach	"The psychological mixed with other things, it's a huge things" [S4 WBF MD MPR]<br>"I think you can help yourself a lot…. By...believing in yourself" [S8 WM LD LPR]
		Effective communication: listen, give time,	"Listening to what I have to say" [S6 PM LD LPR]<br>"They will give me enough time to explain the problem" [S2 WBM MD HPR]<br>"I think, to me its almost just a matter of communicating rather than having a rigid set routine" [S8 WM LD LPR]
		Physical examination	"Give me some sort of examination of the problem area" [S2 WBM MD HPR]
		Clear explanation and understanding of pain	"All I want to know is what's triggering it" [S4 WBF MD MPR]<br>"Explain why and what they're doing in detail that would be understandable, cause sometimes the physio would explain something in their term… like L1 or something, I remember hearing that"[S5 PF HD HPR]<br>"Give you the right advice and guidance"[S3 WBF MD MPR]
		Support and guidance, gain control	"Maybe some follow up consultations to see how have you progressed" [S6 PM LD LPR]<br>"Most importantly that they will give me some advice or treatment, exercise. Something that I can go home and feel that there is progress being made" [S2 WBM MD HPR]
		Increase exercise	"Exercise – yeah well I need to keep doing exercise"[S7 PM MD MPR]<br>"Just some sort of activity I can do that helps it and relieves the pain"[S9 WBF MD HPR]
