# Supplementary material for: Exploring the lived experience and chronic low back pain beliefs of English-speaking Punjabi and white British people: a qualitative study within the NHS
Source: BMJ Open. 2018 Feb 11;8(2):e020108. doi: 10.1136/bmjopen-2017-020108 (PMC5829944; doi:10.1136/bmjopen-2017-020108)
Supplement: Author's manuscript [file bmjopen-2017-020108.draft_revisions.pdf]

BMJ Open

BMJ Open is committed to open peer review. As part of this commitment we make the peer review history of every article we publish publicly available.

When an article is published we post the peer reviewers' comments and the authors' responses online. We also post the versions of the paper that were used during peer review. These are the versions that the peer review comments apply to.

The versions of the paper that follow are the versions that were submitted during the peer review process. They are not the versions of record or the final published versions. They should not be cited or distributed as the published version of this manuscript.

BMJ Open is an open access journal and the full, final, typeset and author-corrected version of record of the manuscript is available on our site with no access controls, subscription charges or pay-per-view fees (http://bmjopen.bmj.com).

If you have any questions on BMJ Open's open peer review process please email info.bmjopen@bmj.com

BMJ Open

Exploring the Lived Experience And Chronic Low Back Pain Beliefs Of White British And English Speaking Punjabi People

Journal:	*BMJ Open*
Manuscript ID	bmjopen-2017-020108
Article Type:	Research
Date Submitted by the Author:	13-Oct-2017
Complete List of Authors:	Singh, Gurpreet; University of Birmingham, School of Sport, Exercise and Rehabilitation Sciences; University Hospitals of Leicester NHS Trust<br>Newton, Christopher; University Hospitals of Leicester NHS Trust<br>O'Sullivan, Kieran; Aspetar Qatar Orthopaedic and Sports Medicine Hospital, Sports Spine Centre<br>Soundy, Andrew; University of Birmingham,<br>R Heneghan, Nicola; University of Birmingham, Centre of Precision Rehabilitation for Spinal Pain, School of Sport, Exercise and Rehabilitation Sciences
**Primary Subject Heading**:	Rehabilitation medicine
Secondary Subject Heading:	Patient-centred medicine
Keywords:	low back pain, lived experiences, cultural beliefs

SCHOLARONE™
Manuscripts

Title

Exploring the Lived Experience And Chronic Low Back Pain Beliefs Of White British And English Speaking Punjabi People

Authors

Gurpreet Singh[1, 2], Christopher Newton [1], Kieran O'Sullivan [3, 4], Andrew Soundy [2], Nicola R Heneghan [5]

Affiliations

1 University Hospitals of Leicester NHS Trust, Leicester, United Kingdom

2 School of Sport, Exercise and Rehabilitation Sciences, University of Birmingham, Birmingham, United Kingdom

3 Department of Clinical Therapies, University of Limerick, Limerick, Ireland

4 Sports Spine Centre, Aspetar Orthopaedic and Sports Medicine Hospital, Doha, Qatar

5 Centre of Precision Rehabilitation for Spinal Pain, School of Sport, Exercise and Rehabilitation Sciences, University of Birmingham, Birmingham, United Kingdom

Correspondence:

Nicola R Heneghan

Centre of Precision Rehabilitation for Spinal Pain (CPR Spine),
School of Sport, Exercise & Rehabilitation Sciences
University of Birmingham, Edgbaston,
Birmingham. B15 2TT. UK
Email: n.heneghan@bham.ac.uk

Tel: +44 121 415 8367

For peer review only

For peer review only - http://bmjopen.bmj.com/site/about/guidelines.xhtml

Abstract

Introduction: Disabling chronic low back pain (CLBP) is associated with negative beliefs and behaviours, which are influenced by culture, religion and interactions with healthcare practitioners (HCPs). In the United Kingdom (UK) HCPs encounter people from different cultures and ethnic backgrounds, with South Asian Indian's (including Punjabis) forming the largest ethnic minority group. Better understanding of the beliefs and experiences of ethnic minorities with CLBP might inform effective management.

Objectives: To explore the CLBP beliefs and experiences of English-speaking Punjabi and White British people living with CLBP, explore how beliefs may influence the lived experience of CLBP and conduct cross-cultural comparisons between the two groups.

Design: Qualitative study using semi structured interviews, set within an interpretive description framework and thematic analysis.

Setting: An NHS Hospital Physiotherapy department, Leicester, UK.

Participants: 10 CLBP participants (5 English-speaking Punjabi and 5 White British) purposively recruited from Physiotherapy waiting lists.

Results: Participants from both groups held negative biomedical CLBP beliefs, experienced unfulfilling interactions with HCP's and negative psychosocial dimensions of CLBP. Specific findings to Punjabi participants included (i) disruption to cultural-religious wellbeing, (ii) disruption to the perceived female 'home-maker' role within the home, as well as (iii) a perceived lack of understanding and empathy regarding their CLBP from the Punjabi community. In contrast to their White British counterparts', Punjabi participants reported initially utilising passive coping strategies, however all participants reported a transition towards active coping strategies.

Conclusion: CLBP beliefs and experiences, irrespective of ethnicity, were primarily biomedically-orientated. However, cross-cultural differences included cultural-religious well-being, the perceived female role, the community response to CLBP experienced by Punjabi

For peer review only - http://bmjopen.bmj.com/site/about/guidelines.xhtml

participants, and coping styles. These findings might help inform management of people with CLBP.

Strengths and limitations of this study

- The first study to provide a cross-cultural exploration of CLBP beliefs and experiences of English-speaking Punjabi and White British people living with CLBP.
- Using purposive sampling, 1:1 semi-structured interviews were conducted through a cultural lens to explore beliefs and experiences of Punjabi and White British people with CLBP.
- The study findings were data-driven and embedded in the participants' voice.
- Participants were all English-speaking and were only selected from one geographical location, which may limit the transferability of the findings.
- Member checking was not conducted to validate interview transcripts.

For peer review only

For peer review only - http://bmjopen.bmj.com/site/about/guidelines.xhtml

Introduction

Chronic low back pain (CLBP) is the primary cause of disability and financial burden to healthcare and society in the United Kingdom (UK).[1] Despite increasing resources spent to improve CLBP management, the associated disability continues to rise.[2]

Historically, the impact of CLBP on developing populations was perceived to be minimal, but is now understood to be similar to western populations.[3-8] In the UK a higher incidence of spinal pain has been reported in South Asians, [9] and a higher prevalence of widespread musculoskeletal pain reported in South Asian Indian Punjabis (22%) compared with Europeans (9%).[10]

Biomedical beliefs about CLBP are common within western populations and are emergent within developing populations.[3 11 12] Factors thought to influence these beliefs include ethnicity, religion, family and friends, as well as unhelpful interactions with healthcare practitioners (HCPs).[13 14]

Current CLBP research has largely focused on western societies with little emphasis on minority ethnic populations; a concern given ethnic migration and cultural diversity is increasing within western societies. According to the last UK Census (2011)[15] Leicester hosts the largest Indian (referring to themselves as Asian or British Asian) population (30%) of any local authority in England and Wales.

Understanding cultural variations in pain perception, beliefs, expectations, and behaviours is important to accurately identify patients' needs and behaviours relative to one's own potentially divergent culture.[16] This may help avoid health inequalities and suboptimal outcomes.[17 18] To tailor management, HCPs might benefit from understanding an individual's beliefs and experiences of CLBP within the cultural context in which they occur.[7 19] Therefore, research is required to understand the management of CLBP within different populations within the UK. Cross-cultural comparative studies utilising qualitative

For peer review only - http://bmjopen.bmj.com/site/about/guidelines.xhtml

For peer review only

methodologies may provide in-depth understanding of individual and culture specific beliefs and experiences of CLBP. However, the authors are not aware that any such comparisons have been made between English speaking Punjabi and White British CLBP populations.

Therefore, this study aimed to investigate the beliefs and experiences of people living with CLBP in English speaking Punjabi and White British populations. The objectives were to explore how these beliefs influence, and impact on, the experience of living with CLBP and identify similarities and differences between the two ethnic groups.

For peer review only

For peer review only - http://bmjopen.bmj.com/site/about/guidelines.xhtml

Methodology

Using semi structured interviews this study followed the COnsolidated criteria for REporting Qualitative studies (COREQ) (Supplementary File 1).[20] Interpretative description (ID) was chosen as this qualitative approach has been specifically developed for healthcare enquiries of a clinical phenomenon, using subjective accounts, for the purpose of informing clinical understanding.[21] Prior theoretical and clinical knowledge is valued as a starting point for research in ID, although this can be challenged and developed as the research progresses.[22]

Sample

Purposive sampling[23] was employed to recruit White British and Punjabi participants sufficient to enable relevant data to be obtained and analysed.[24] The study setting was Leicester (UK), which hosts a large White British and Punjabi population.

Eligible participants were White British and English speaking Punjabi people aged 18-65 years with CLBP of ≥ 6-month duration.[25] Individuals with a previous history of surgery for CLBP, diagnosed with a specific or a serious underlying cause of their CLBP (i.e. fracture, infection, inflammatory spondyloarthropathy, cancer, or nerve root compression)[26 27] or who had previous physiotherapy treatment from the authors (GS, CN) were excluded.

Recruitment

Potential participants were identified by GS following a GP or Consultant referral to an NHS Physiotherapy department for CLBP between April 2014 and April 2015. Study information was posted to eligible individuals with their physiotherapy appointment letter. Following this, an interview was arranged via telephone, prior to physiotherapy commencing. GS obtained written informed consent pre-interview. Recruitment continued until saturation was

For peer review only - http://bmjopen.bmj.com/site/about/guidelines.xhtml

achieved.[28] All participants were eligible and included. The sample comprised of five White British (2 males: 3 females) and five English speaking Punjabi (3 males: 2 females) people, with a mean age of 40 years (Table 1).

Data collection

In-depth semi-structured interviews were conducted by GS (British Punjabi male) or CN (White British male), with 11 and 13 years musculoskeletal physiotherapy experience respectively, with a special interest and *a-priori* knowledge of CLBP. Both authors undertook three hours of National Institute of Health Research training on semi-structured interviewing.[29] No prior relationship was established with participants. Interviews took place in a quiet room in the physiotherapy department and lasted between 60-70 minutes.

A topic guide was informed by contemporary CLBP literature,[7 13] and research team expertise. Further refinements were made following two pilot interviews with CLBP patients.[30] The topic guide included open-ended questions related to the individual's 'story of their CLBP', their beliefs about causation, management and the future) as well as the lived experience of CLBP (related to interaction with HCPs, coping with CLBP and its personal, psychological, social and cultural impact) (Supplementary File 2).

Participants provided demographic data and completed validated questionnaires for pain severity (Numerical Pain Rating Scale was a sub-item score from the Short Form Orebro Musculoskeletal Questionnaire (SFOQ)),[31] functional disability (Oswestry Disability Index)[32] and psychosocial risk profile (including sleep, anxiety, depression, catastrophising, fear-activity and fear-work were sub-item scores from the SFOQ)[31] (Tables 1 and 2).

Data analysis

Interviews were audio-recorded and transcribed verbatim by GS who analysed the data using thematic analysis.[33] Each transcript was analysed line-by-line using an iterative model

For peer review only - http://bmjopen.bmj.com/site/about/guidelines.xhtml

immediately after the first interview. This involved: data sampling, collection and analysis occurring in tandem as an on-going constant comparative process[34] to facilitate the capture of emergent themes during data collection.[33] This process allowed active engagement and familiarisation with the data and some of the terms for beliefs and coping were identified from previous literature.[7 13] From this, initial themes were generated and data-driven coding facilitated the development of a thematic table, which was modified as data analysis and interpretation evolved (Table 3). Crucially this involved critique by the co-authors (CN, KOS, AS and NH)[35] to enhance rigour and trustworthiness of study findings.[23] GS, CN and AS independently assessed the accuracy and completeness of all the transcripts, ensuring these related to the thematic development and emerging themes; this process was collated as an audit trail (Supplementary File 3). Data collection and analysis was transparent and detailed.

For peer review only

Results

Five main themes emerged from the interviews: i) biomedical back pain beliefs, ii) coping with CLBP, iii) the psychological and emotional dimensions of living with CLBP, iv) the social and cultural-religious impact of CLBP and v) reflecting on HCP interactions, management experience and expectations of future management. These themes are presented in a compare/contrast style between the ethnic groups. Due to the commonalities between the two groups, the findings presented apply to both groups unless otherwise stated (Supplementary File 4).

Table 1: Participant Characteristics

Participant code	CLBP duration (years)	Age (years)	Sex	Ethnicity
S1	25	40	Male	Punjabi
S2	2	51	Male	White British
S3	18	35	Female	White British
S4	20	42	Female	White British
S5	2	23	Female	Punjabi
S6	7	37	Male	Punjabi
S7	4	40	Male	Punjabi
S8	1	53	Male	White British
S9	2	49	Female	White British
S10	14	38	Female	Punjabi

For peer review only - http://bmjopen.bmj.com/site/about/guidelines.xhtml

Table 2: Participant pain, disability and psychosocial risk profile data

Participant code	NPRS	ODI (%)	SFOQ	Sleep	Anxiety	Depression	Catastrophising	Fear-Activity	Fear-Work	Employment Status
S1	7	(Moderate) 28	(Moderate) 48	4	5	4	7	7	3	Working
S2	8	(Moderate) 38	(High) 59	1	6	5	10	5	5	Working
S3	5	(Moderate) 24	(Moderate) 46	6	0	0	5	10	5	Working
S4	8	(Moderate) 30	(Moderate) 48	5	7	2	10	2	2	Retired
S5	3	(High) 51	(High) 50	7	6	7	5	0	1	Off work
S6	2	(Low) 14	(Low) 22	2	0	0	3	4	3	Working
S7	5	(Moderate) 24	(Moderate) 41	6	3	2	8	7	1	Working
S8	2	(Low) 14	(Low) 29	2	3	1	3	8	3	Working
S9	4	(Moderate) 40	(High) 73	7	8	9	8	9	3	Working
S10	8	(High) 50	(High) 77	7	1	6	10	10	7	Working

ODI = Oswestry Disability Index, SFOQ = Short Form Orebro musculoskeletal pain questionnaire, NPRS = Numeric Pain Rating Scale

For peer review only

For peer review only - http://bmjopen.bmj.com/site/about/guidelines.xhtml

Table 3: The thematic development illustrating key stages

	Thematic development stage 1		Thematic development stage 2	Thematic development stage 3	Final theme stage 4
**Theme 1**	Back pain beliefs	Revised Topic guide	Back pain beliefs	Biomedical Back pain beliefs	Biomedical Back pain beliefs
**Theme 2**	Coping with CLBP		Coping with CLBP	Coping with CLBP	Coping with CLBP
**Theme 3**	Psychosocial, dimensions of pain		Impact of CLBP	Psychological, emotional and social dimensions of CLBP	The psychological and emotional dimensions of living with CLBP
**Theme 4**	Cultural impact of CLBP		Cultural impact of CLBP	Cultural impact of CLBP	The social and cultural-religious impact of CLBP
**Theme 5**	HCP interactions		HCP interactions, management experience and future expectations	HCP interactions, management experience and future expectations	Reflecting on HCP interactions, management experience and expectations of future management

CLBP = Chronic low back pain
HCP = Health care practitioner

For peer review only - http://bmjopen.bmj.com/site/about/guidelines.xhtml

Theme 1: Biomedical back pain beliefs

Cause of CLBP attributed to physical and structural/anatomical factors

All participants held similar biomedical CLBP beliefs. Common causal beliefs attributed CLBP to physical and structural/anatomical factors. These mainly included bending and lifting strains, for example:

> *"I basically bent down to pick up a pen or something and it clicked and I couldn't straighten myself up"* [S6]

Consistent with these beliefs, in cases where a physical causal mechanism could not be recalled, participants self-diagnosed a structural/anatomical cause for their CLBP. The most frequently expressed labels included: 'slipped disc' (n=5), 'wear and tear' (n=3) and 'trapped nerve' (n=3).

Recalling HCPs biomedical diagnosis and the biomedical beliefs adopted

Most participants recalled a diagnostic label derived from HCPs embedded within the biomedical model, consistent with their own beliefs. Nonetheless, some interpreted this information negatively. Following a consultation with a chiropractor, one participant perceived his back *"...was out of place"* [S7]

Biomedical CLBP beliefs were influenced by manual-handling training and by participants' occupation. A nurse believed the repetitive nature of manual handling in ward settings to be a cause of his CLBP. Subsequently, participants' adopted the belief their spine needed protecting when bending and lifting:

> "I suppose if you lift incorrectly it will cause back pain. I mean we have all had it drummed in to us (manual-handling training), how to bend our knees and how to do all that malarkey" [S2]

Vulnerability of the spine

Many viewed their spine as vulnerable, central to function and critical to one's well-being. Given participants' had 'one back', that was hard to see, combined with a feeling of weakness, the back was commonly described as 'precious', and when compared to an ankle sprain required more protection:

For peer review only - http://bmjopen.bmj.com/site/about/guidelines.xhtml

"It just feels as though the spine controls so much of your function in your legs and everything, that if you injure it, it's much more serious than perhaps injuring your ankle" [S2]

Consistent with higher levels of disability, participants' fear avoidant beliefs suggested they believed pain indicated harm, and was a warning signal from the back:

"*your back is trying to tell you something. It's trying to tell you to stop doing whatever you're doing if it's hurting…because you are making it worse."* [S3]

<u>*Future outlook*</u>

Participants tried to maintain a positive future outlook; this was mainly based on prior pain experiences, and individual personalities.

"I even think now that I will wake up and it will all go away and it maybe will." [S2]

Despite this, there was an overriding feeling of uncertainty:

"*I don't want to think about that because I don't know how I'm going to be*" [S5]

For some Punjabi participants' religion was expressed as a key part of their lives and interlinked with their positive outlook on pain. One participant described a hand injury whereby his fingers were amputated *as 'God's will'* and expressed his positive outlook in the context of his religious beliefs:

"Someone up there wanted them so they were gone...my sin plate was wiped clean on that day and we start again." [S1]

Theme 2: Coping with CLBP

<u>*Active coping strategies*</u>

White British participants' predominantly demonstrated active coping 'self-help' strategies based on prior CLBP experiences and searching online for knowledge and understanding:

"I went onto NHS choices and typed in sciatica - just that word and a whole load of stuff comes up and there is one big sentence and it says 'keep active…." [S2]

Amongst the White British group, exercise strategies such as stretching, yoga and football were utilised. They commonly shared the narrative 'confronting pain and battling on'.

For peer review only

Influenced by a need to avoid interference in one's life, fulfill family and parental duties and to avoid burdening others, this often meant enduring pain.

"I even think now that I will wake up and it will all go away and it maybe will." [S2]

Reliance on HCPs and biomedical interventions

A lack of understanding and control over pain led some participants to rely on HCPs in an unrelenting search for a diagnostic label, whilst others sought reassurance via scans:

"I wanted to have a scan just to see if there was anything major ermmm major wrong" [S9]

Both groups expressed varying levels of reliance on medication, ranging from daily use, to situations of desperation where they felt 'physically stuck' or to prevent pain intensifying.

In contrast to White British participants, Punjabi participants' demonstrated a passive reliance on HCPs to provide 'quick fix" interventions including acupuncture, massage and most frequently manual therapies:

"you go there (manual therapist) for a quick fix" [S6]

Protective and avoidance coping strategies

In terms of bending, lifting and heavy physical tasks, many participants used protective and avoidance strategies. Meanwhile, experiencing exercise-related pain, increased fear avoidance around exercise, which promoted resting behaviours. Uncertainty about the cause of pain increased hypervigilance to the threat of pain and adherence to manual handling advice to protect the back reflecting a belief of spinal vulnerability:

"I think a bit more…. if I have a task that it would probably involve lifting a box or whatever, I will now consciously think, make sure you bend your knees and you keep your back straight if you're lifting something or whatever. Whereas previously you think you are fine, it doesn't matter, nothing is going to happen so…" [S1]

Coping transition

An interesting difference emerged in the coping trajectories of White British and Punjabi participants. Although, it was not clear how this transition took place, all Punjabi participants' reported a transition from a passive reliance on HCPs for a "quick fix" to more active coping

For peer review only - http://bmjopen.bmj.com/site/about/guidelines.xhtml

strategies such as self-searching the internet, as well as replacing rest with increased physical and social activities in order to resume normal life:

> *"Instead of relax it…. I'd just go back to my normal routine"* [S6].

Theme 3: The psychological and emotional dimensions of CLBP

Psychological and emotional consequences

Across both groups, a number of negative psychological and emotional dimensions of CLBP were expressed. Those with more disabling CLBP most frequently reported depressed mood, hopelessness, frustration, catastrophising thoughts, and uncertainty about pain and lack of control over it.

Depressed mood was most often expressed in relation to the persistence of CLBP, and as a consequence of failed interventions, disruption to sleep and engagement in meaningful activities. Some attributed disabling CLBP to weight gain resulting in depressed mood:

> *"because I put on a lot of weight when I was bed bound that got me really down"* [S5]

Hopelessness was attributed to a lack of control over pain and a lack of support from HCPs, who in some cases painted a pessimistic outlook:

> *"No I mean they just said it's just down to wear and tear basically and you've got to live with it"* [S9]

Frustration was repeatedly expressed in relation to a lack of understanding of the cause of pain, and the interference with daily life, and in some cases the desire to exercise and lose weight. Participants with more disabling pain held catastrophic CLBP thoughts. Similar to depressed mood and frustration, this was associated with diagnostic uncertainty and a lack of control over pain, resulting in catastrophising about the future:

> *"I might not walk again"* [S5]

Catastrophic nature of pain flare-ups

Often participants' used catastrophic language to portray the unpredictable, fluctuating and uncontrollable disabling impact of flare-ups, for example:

For peer review only - http://bmjopen.bmj.com/site/about/guidelines.xhtml

"I would say at it's very worst point it feels like a ball of fire. It's debilitating to the point where I want to cut my left-side off. It's awful and the longer the pain continues the worse it seems to spread" [S4]

For some this entailed a physical and emotional struggle. However only a few seemed to form a link between their thoughts and CLBP:

"When I have got a project or something... I am not thinking about my back at all. I am just cracking on. I'm noticing it more because I have got time on my hands" [S4]

Threat to self-identity

Cross-cultural comparisons highlighted that CLBP posed a major threat to participants' *'self' and their identity. S*ome described negative consequences of CLBP such as low self-esteem, reduced independence and disempowerment. One participant expressed disabling CLBP as a major loss:

"Everything is just gone now like dignity, confidence. I had a stick but I would only use that sometimes if I was going out" [S5]

During flare-ups, a small number expressed difficulties carrying out daily functional activities. The greatest impact seemed to be on bending-related tasks such as putting on socks and hoovering. This led to dependency on family members and changing roles in their relationships, which negatively impacted participants' psychological and emotional well-being.

Theme 4: The social and cultural-religious impact of CLBP

Threat to family/friend relationships and social life

Mostly expressed by White British participants', the interference of CLBP on family relationships and fulfilling parental roles was described as 'restrictive' on their spouse/partner, often eliciting negative emotions in family members. One participant identified her coping strategies as a potential cause of frustration:

"My husband also gets frustrated with me. He says 'what you being a legend for, why don't you just sit down and do it later'. I don't know… he still thinks I have OCD" [S4]

For peer review only - http://bmjopen.bmj.com/site/about/guidelines.xhtml

CLBP inhibited social interactions across both groups. Social isolation was the consequence for some previously sociable participants' with more disabling pain:

> *"Just walking round town I have to stop and go in a café and have a coffee just to sit down to rest my back. I stay at home now"* [S9]

<u>*Work impact*</u>

Disrupting work roles, those sitting at work constantly fidgeted and had move in an attempt to control pain. Participants also commented how CLBP impacted on colleagues. Sickness absence was discussed by most with varied views, some cited having time off work due to a flare-up:

> *"I did have time off 'cos my back was hurting too much"* [S7]

<u>*The impact of CLBP on cultural and religious wellbeing*</u>

CLBP negatively impacted cultural-religious wellbeing of Punjabi participants, consistently impeding meditation, particularly as this involved sitting cross-legged on a floor for long periods:

> *"I do sit down crossed legs on the floor when we pray and meditate and maybe that has slight impact on it, you know when it gets just maybe it gets tired"* [S6]

This impact varied from 'the hips and back locking', to an inability to sit leaving one participant secluding herself from religious rituals that involved sitting:

> "[Referring to sitting in the temple] I'll just go when it's quiet y'know, ermmm do my praying and then come back out and then just go downstairs where there's chairs or.. I can just hang around somewhere else and that kind of…I feel excluded sometimes because y'know people tend to like wanna sit upstairs and I have to like go just downstairs" [S10]

Cultural roles and obligations were impacted. In this context, one Punjabi female described the 'perceived' female cultural role and how CLBP disrupted her ability to carry out household duties including cooking. Viewed as essential to the female role amongst Punjabi's, difficulties with or an inability to carry out these duties had far-reaching consequences such as finding a marriage partner:

For peer review only - http://bmjopen.bmj.com/site/about/guidelines.xhtml

"With the Asian culture a girl has to do housework - she has to get prepared for her married life, so she has to learn how to cook, she has to learn how to do housework, look after her husband and when you can't do that, you're you know not suitable anymore" [S10]

The response of family, friends and wider community to CLBP

Participants' reported experiencing varied responses from friends and family members to their CLBP. These included feeling pushed to seek healthcare to 'get it fixed', feeling supported in some cases, and over supported in others. In contrast, one Punjabi participant experienced very little empathy and support:

"My family don't take me seriously anymore because they're just sick of hearing about it and my friends just don't understand" [S10]

Some Punjabi participants' felt stigmatized, with cultural comparisons indicating a perceived lack of empathy and understanding from people within the Punjabi community:

"In other cultures do kind of ... they tend to be a bit more understanding" [S10]

CLBP advice from Punjabi community members, the self-acclaimed 'back pain experts', left one participant frustrated:

"*Unfortunately we live in a community that everyone thinks they are a qualified doctor. You know, don't do this, do this sort of a thing*" [S1]

Recalling family experiences of CLBP

Participants recalled back pain experiences of family members with regards to their coping strategies, levels of disability and interactions with HCPs. Some family members reluctantly relied on medication, whilst others adopted active coping strategies and demonstrated self-efficacy. However, participants did not consistently adopt the coping strategies observed. For example, one Punjabi participant initially utilised passive interventions (including massage) in contrast to his father:

For peer review only - http://bmjopen.bmj.com/site/about/guidelines.xhtml

"He has not let it really impact him to be honest because he still goes to the gym, he still lift weights, just does everything. Before every now and again his back hurts a little bit. He does what he does - he hasn't changed anything" [S6]

Theme 5: Reflecting on HCP interactions, management experience and expectations of future management

Varying quality of therapeutic alliance

Reflecting on previous HCP interactions, many experienced variations in therapeutic alliance, with mostly negative interactions. Strong therapeutic alliance was associated with HCPs providing clear communication. This included a clear explanation about the cause of pain, as well as reassurance, collaborative compassionate care with on-going support and guidance. This increased adherence to treatment, built empowerment and trust in their HCPs:

"*I've great faith in the physio*" [S2]

In contrast, several factors were attributed to weak therapeutic alliance. Some depicted a power struggle, where the HCP was in control*, and* access to investigations such as MRI scans was rejected, others reported a lack of individualised holistic care. However, most cited HCPs communication as a major problem, particularly not being given time, not being taken seriously, not feeling understood nor listened to. One participant reported feeling disrespected:

"It really did upset me when I went to see a consultant - I felt very belittled by how he approached me" [S9]

Expectations were often unmet, where participants did not receive investigations, a clear diagnosis, a physical examination or manual therapy whilst enduring unexpected pain flare-ups. A perceived lack of guidance and support from HCPs was also cited, in some cases resulting in feelings of helplessness and low mood.

For peer review only - http://bmjopen.bmj.com/site/about/guidelines.xhtml

Interpreting the HCP explanation

Iatrogenic language used by HCPs was fear-inducing for some participants. Interpretations of HCPs explanations led to a sense of vulnerability around the spine and a need to adopt caution. One participant in his thirties recalled his interpretation of 'wear and tear':

> *"It makes you think that something you've got something permanent and you're basically going to have to live with it"* [S6]

However, uncertainty about the actual cause of their pain was most common, partly due to mixed messages conveyed by HCPs and inconclusive radiological investigations.

Appraising interventions and ability to control CLBP

Reflecting on prior CLBP management, this was embedded within the biomedical model for the majority. Treatment interventions were appraised in relation to their therapeutic effect and the participant's ability to control their pain. Medications, despite being taken for long periods were deemed largely ineffective by many, as was acupuncture. In addition, a reliance on manual therapies offered minimal long-term effect:

> *"If I'm honest - at the time it's a psychological plaster"* [S4]

Expectations of future management

Interestingly, when exploring cross-cultural expectations of future management, all participants' sought an individualised, mind-body approach, which involved a physical 'hands on' examination. Many expressed the need for HCPs to possess strong communication skills which include: empathy, active listening skills, providing time, clear communication and to:

> *"Explain things thoroughly, don't frighten the patient, and just generally be welcoming"* [S3]

For peer review only - http://bmjopen.bmj.com/site/about/guidelines.xhtml

Discussion

This is the first study to examine the lived experience and CLBP beliefs of White British and English speaking Punjabi people. Our findings suggest several between-group similarities including biomedical back pain beliefs, unfulfilling HCP interactions and negative psychological emotional and social influences of CLBP. Differences included CLBP disrupting Punjabi participants' participation in cultural-religious activities, and the perceived female role within the home. Whilst White British participants adopted active coping strategies, their Punjabi counterparts initially reported a preference for passive coping strategies, but all reported a transition to active coping strategies. Punjabi participants also experienced a lack of empathy and understanding from the Punjabi community.

Biomedical beliefs

Biomedical CLBP beliefs were common amongst all participants, their family, friends and particularly the wider Punjabi community. This supports the view that biomedical beliefs may not be exclusive to certain populations, instead reflecting the views of western society overall.[8 36] Biomedical beliefs conveyed by HCPs were adopted by, or were similar to those already held by, participants', consistent with other studies.[13 37] These beliefs were often associated with negative CLBP information,[38] around bending and lifting, perpetuating beliefs of spinal vulnerability culminating in fear avoidance beliefs and behaviours.[39] Further, the role of cultural-religious pain beliefs in promoting a positive future outlook has been documented in other cultural groups.[40] However only one Punjabi participant expressed pain beliefs within a positive cultural-religious context, perhaps reflecting participants' predominantly biomedical beliefs.

Coping strategies and transition

Illustrated as an on-going challenge by many participants, coping strategies have been shown to influence the development and persistence of CLBP.[41] Most White British

For peer review only - http://bmjopen.bmj.com/site/about/guidelines.xhtml

participants at times utilised active coping strategies such as self-searching for knowledge and exercises, thus demonstrating self-efficacy. A recent qualitative CLBP study[42] reported patients require an explanation and understanding of their CLBP, consistent with our study. Many searched relentlessly, primarily via reliance on HCPs for biomedical interventions, perhaps reflecting the importance participants attached to finding a diagnosis that would legitimise their pain.[19] Meanwhile, Punjabi participants' initial reliance on HCPs to provide passive 'quick fix' interventions and a dependency on family members may highlight their biomedical beliefs, underpinned by a lack of understanding and control over pain resulting in low self–efficacy. In support, passive coping strategies have been identified in a previous UK study among a South Asian population with chronic pain.[43] However, acculturation levels were low and perhaps participants lacked knowledge about western medicine. Other UK studies in South Asian populations have identified a reliance on 'complementary' medications.[44] In contrast, in our study Punjabi participants' pursued more conventional western medications and interventions. This may reflect greater awareness of, or access to, these treatment options. A novel finding of our study is that all Punjabi participants reported a transition from passive to active coping strategies.[3 5] This may reflect the limited effects of passive interventions, as well as greater knowledge and understanding of CLBP and the potential benefits of active coping strategies. Alternatively, this group may have complied with the HCPs approach to management even if it was not their preference.

Psychological and emotional dimensions

The impact of CLBP has been found to extend beyond physical domains,[3] with many negative and often life-changing psychological and emotional effects.[19 36 45] Contrary to earlier research,[46] participants did not appear to consider these factors as contributors to CLBP, instead viewing these as secondary effects of CLBP. Supporting this, one study found South Asians were unwilling to recognise the influence of psychological, emotional and social factors on their pain.[43] However, negative beliefs about the control of CLBP and the resulting passive coping often reported by participants may have a mediating influence

For peer review only - http://bmjopen.bmj.com/site/about/guidelines.xhtml

on depressed mood, pain and disability.[47] Feelings of frustration were common amongst participants' due to a lack of explanation and understanding about CLBP, including inconclusive diagnostic radiological investigations. This may reflect their desire for answers linked with pain legitimisation and validation.[48] Contrary to previous research,[49] perceptions of 'not feeling believed' were not consistent with participants' views, nor were feelings of anger and perceived injustice associated with the negative impact of CLBP. Perhaps, these feelings did exist, but were not expressed due to fear of being judged or it negatively impacting on physiotherapy. Furthermore, our findings lend support to a study showing catastrophic thoughts were associated with a magnified threat to the 'unpredictable' and 'fluctuating' nature of pain flare-ups, excessive worry about pain and a pessimistic view of controlling pain.[50]

Social and cultural-religious dimensions

Similar to other studies, CLBP was highly disabling, described as a 'major loss' by some. It impacted on many aspects of individual's lives including their identity, self-esteem and independence, leaving some disempowered.[45 51 52] For many, this meant their lives were 'on hold', a finding consistent with a recent systematic review.[36] Our data supports the notion that CLBP impacts meaningful relationships, threatening parental and family duties and for some resulting in social isolation.[3 5 45] With regards to work, although participants' demonstrated some avoidance behaviours in the workplace, at the time of interviewing only one participant was absent from work due to CLBP. This may reflect active coping in relation to work, possibly influenced by financial concerns associated with sickness absence, good work support and job satisfaction [53] or positive HCP advice relating to work.

Therapeutic alliance and a person-centred approach

Strong therapeutic alliance in the management of CLBP has been associated with greater treatment compliance, improved clinical outcomes[54] and greater levels of patient

For peer review only - http://bmjopen.bmj.com/site/about/guidelines.xhtml

satisfaction.[55] However, most participants' experienced weak therapeutic alliance. Associated with weak therapeutic alliance,[56] the management of CLBP for most was viewed as lacking an individualised and holistic approach. Furthermore, participants' predominantly experienced a lack of guidance and support, and poor HCP communication.[19] Examination of HCP communication revealed a lack of clear explanation and participants' understanding about pain, instead creating uncertainty for many. Language has been identified as an important facet of effective communication given it is personal and variable, particularly amongst different ethnic populations. Consistent with findings in other populations,[7] HCPs commonly used fear-inducing language, which in combination with biomedical CLBP beliefs and poor HCP communication is linked to weak therapeutic alliance and CLBP related disability.[57] These factors may reflect HCPs lack of interpersonal skills, particularly specialised communication skills, and their over-utilisation of biomedical approaches to CLBP management,[58] posing a greater challenge to managing ethnic minority populations. These findings question how HCPs interact with people living with CLBP. HCPs may require training to enhance their communication skills and partnerships with patients.[59 60]

It is also worth noting that participants' in our study did not experience inequalities in accessing care or treatment, contrary to findings in other South Asian ethnic populations,[61 62] who utilised participants' with low acculturation levels. Participants' experience of HCP interactions highlighted a biomedical approach to CLBP. This may be due to the influence of HCPs biomedical CLBP beliefs on their clinical management.[13] Interestingly, most participants' sought an individualised self-management approach[59 60 63] delivered by empathetic HCPs with effective communication,[64] perhaps more aligned with the bio-psychosocial model. This quest, along with Punjabi participants' transition to seek active coping strategies, demonstrates a desire for self-efficacy, which has been linked with reduced disability.[65] HCPs biomedical preference for managing CLBP may highlight discordance with the bio-psychosocial model advocated by recent NICE guidelines,[69] and has been associated with poor adherence to treatment.[13] Thus more individualised multi-

For peer review only - http://bmjopen.bmj.com/site/about/guidelines.xhtml

dimensional approaches to management,[66] built on effective communication facilitating strong therapeutic alliance[67] and self-management might be needed.

Cultural differences

To date, qualitative research in the UK has paid little attention to the CLBP experience through a cultural and ethnic lens. This requires consideration, given the degree to which individuals identify with their ethnic group, share beliefs and engage in culture roles, which can influence pain experiences.[40] Consistent with other ethnic minority CLBP studies,[7 11] Punjabi participants' experienced a disruption to cultural-religious wellbeing, and endured a negative response from the wider community. In this context, some novel and potentially important themes were identified. As Punjabi participants expressed, religion maybe important to people in ethnic minority populations,[40] disruption to sitting-based meditation in some cases resulted in immense frustration and isolation. In some populations the perceived view of the female role involves housework and preparing for marriage, or serving the husband.[3 11] This perception appears similar to those held within the Punjabi community. CLBP undermined the ability to carry out these duties. For one participant this created uncertainty about finding a marriage partner, and feelings of guilt and burden on other female family members, due to increased workloads placed upon them. Our findings support those in other ethnic populations where gender differences exist in the experience of CLBP.[3 5] One novel finding of our study was that Punjabi participants' perceived a lack of empathy and understanding from the Punjabi community, and for some CLBP was a source of stigmatisation.[59] These factors may reflect cultural attitudes towards people with CLBP within Punjabi communities. Influencing factors may include biomedical beliefs held or limited understanding of CLBP or because stoicism and playing down pain may be more acceptable than gesturing emotion.[68] Findings of this study illustrate the existence of cultural-religious and gender differences specific to Punjabi participants. It is noteworthy that while the recent NICE guidelines[69] call for a bio-psychosocial approach to CLBP, there is no specific guidance on how to acknowledge or manage socio-cultural factors and beliefs.

For peer review only - http://bmjopen.bmj.com/site/about/guidelines.xhtml

Consequently, it may be challenging for HCPs to provide individualised, culturally sensitive bio-psychosocial management for CLBP patients from different ethnic populations.

Strengths limitations and implications for future research

One of the study strengths is its relevance to clinical practice. This is the first cross-cultural study to explore CLBP beliefs and experiences in English speaking Punjabi and White British people living with CLBP. The study findings were data-driven and embedded in the participants' voice. Reflexivity was demonstrated throughout with the authors declaring how their ethnicity; novice researcher role, special clinical interest in CLBP and *a-priori* knowledge may have influenced data collection, analysis and interpretation.

Member checking was not conducted to validate interview transcripts due to time and funding. High acculturation levels and variations in socio-demographic status, may limit the transferability of the findings. Therefore, future research could consider Punjabi populations with low acculturation rates in different geographical regions.

Implications for practice and policy

This study contributes to existing knowledge by providing HCPs managing CLBP in White British and English-speaking Punjabi people new insights,

Which could improve CLBP management within these groups. There may be specific training needs for HCPs to better understand the multifactorial nature of CLBP, specifically the individual's beliefs and experiences within their psychosocial and cultural-religious context.[58 70] This, in addition to developing a flexible communication style that facilitates strong therapeutic alliance may help tailor management within a person-centred approach. Other HCP priorities could include disseminating evidence-based beliefs amongst patients and the public including ethnic minority populations.[7 47]

For peer review only - http://bmjopen.bmj.com/site/about/guidelines.xhtml

Conclusion

CLBP beliefs and experiences similar across both groups were biomedically-orientated. CLBP was associated with negative psychological and social consequences. Cross-cultural differences related to the negative impact on cultural-religious aspects of Punjabi participants' lives particularly the perceived female role. Punjabi participants also reported a transition from passive to active CLBP coping strategies and experiencing a lack of empathy from Punjabi community members. HCPs should therefore adopt a culturally-sensitive approach to the management of CLBP, which considers individuals' beliefs and experiences.

Acknowledgements

Thanks to all participants who took the time to share their stories and experiences.

Footnotes

Twitter: Follow @gsingh1902, @ChrisNewtonPT, @A_Soundy, @kieranosull @HeneghanNicola

Contributors: GS together with CN, KOS, AS and NH were responsible for the conception and design of the study. GS and CN were responsible for data collection. GS was responsible for transcription, leading data analysis and initial drafting of the article. GS, CN, KOS, AS and NH contributed to analysis, interpretation and manuscript development. All authors approved final submitted manuscript.

Funding: GS time funded by National Institute of Health Research

Data sharing: Data retained by the corresponding and lead author, available as requested.

Competing interests: None Declared

Ethics Approval: Ethical approval was obtained from NRES Committee, London – Riverside, Reference Number: 14/LO/0510.

For peer review only

For peer review only - http://bmjopen.bmj.com/site/about/guidelines.xhtml

References

1. Hoy D, Brooks P, Blyth F, et al. The epidemiology of low back pain. *Best Pract Res Clin Rheumatol* 2010;24:769-81. doi.org/10.1016/j.berh.2010.10.002.
2. Freburger JK, Holmes GM, Agans RP, et al. The rising prevalence of chronic low back pain. *Arch Intern Med* 2009;169:251-58. doi:10.1001/archinternmed.2008.543.
3. Tavafian SS, Gregory D, Montazeri A. The experience of low back pain in Iranian women: a focus group study. *Health Care Women Int* 2008;29:339-48 doi.org/10.1080/07399330701876356.
4. Snelgrove S, Liossi C. An interpretative phenomenological analysis of living with chronic low back pain. *Br J Health Psychol* 2009;14:735-49 doi: 10.1348/135910709X402612.
5. Lin IB, O'sullivan PB, Coffin JA, et al. 'I am absolutely shattered': The impact of chronic low back pain on Australian Aboriginal people. *Eur J Pain* 2012;16:1331-41 doi:10.1002/j.1532-2149.2012.00128.x.
6. Scheermesser M, Bachmann S, Schämann A, et al. A qualitative study on the role of cultural background in patients' perspectives on rehabilitation. *BMC Musculoskelet Disord* 2012;13:5.doi.org/10.1186/1471-2474-13-5.
7. Lin IB, O'Sullivan PB, Coffin JA, et al. Disabling chronic low back pain as an iatrogenic disorder: a qualitative study in Aboriginal Australians. *BMJ open* 2013;3:e002654.dx.doi.org/10.1136/bmjopen-2013-002654.
8. Darlow B, Dean S, Perry M, et al. Easy to harm, hard to heal: patient views about the back. *Spine* 2015;40:842-50. doi: 10.1097/BRS.0000000000000901.
9. Webb R, Brammah T, Lunt M, et al. Prevalence and predictors of intense, chronic, and disabling neck and back pain in the UK general population. *Spine* 2003; 28:1195-1202.
10. Palmer B, Macfarlane G, Afzal C, et al. Acculturation and the prevalence of pain amongst South Asian minority ethnic groups in the UK. *Rheumatology* 2007;46:1009-1014. doi.org/10.1093/rheumatology/kem037.
11. Madden V, O'Sullivan P, Fisher J, et al. Our training left us unprepared-two physiotherapists' reflections after working with women with low back pain in a rural Zulu community in South Africa. *J Community Health* 2013;8(2).
12. Zusman M. Belief reinforcement: one reason why costs for low back pain have not decreased. *J Multidiscip Healthc* 2013;6:197. doi.org/10.2147/JMDH.S44117.
13. Darlow B, Fullen BM, Dean S, et al. The association between health care professional attitudes and beliefs and the attitudes and beliefs, clinical management, and outcomes of patients with low back pain: a systematic review. *Eur J Pain* 2012;16:3-17. DOI: 10.1016/j.ejpain.2011.06.006.

14. Schulz PJ, Hartung U, Riva S. Causes, coping, and culture: a comparative survey study on representation of back pain in three Swiss language regions. *PLoS one* 2013;8(11):e78029.doi.org/10.1371/journal.pone.0078029

15. Census Key Statistics for Local Authorities in England and Wales [online]. Office for National Statistics (2011) http://www.ons.gov.uk/ons/rel/census/2011-census/key-statistics-for-local-authorities-in-england-and-wales/rpt-ethnicity.html#tab-Differences-in-ethnicity-across-local-authorities [Accessed 20th January, 2015].

For peer review only - http://bmjopen.bmj.com/site/about/guidelines.xhtml

16. Hobara M. Beliefs about appropriate pain behavior: cross-cultural and sex differences between Japanese and Euro-Americans. *Eur J Pain*2005;9(4):389–93 doi: 10.1016/j.ejpain.2004.09.006.

17. Brockopp DY, Ryan P, Warden S. Nurses' willingness to manage the pain of specific groups of patients. *Br J Nurs*.2003;12(7):409-15 doi.org/10.12968/bjon.2003.12.7.11261
18. Nazroo J. Health and Social research in Multiethnic Societies. Oxford and New York: Routledge 2006.
19. Snelgrove S, Liossi C. Living with chronic low back pain: a metasynthesis of qualitative research. *Chronic Illn*2013;9(4):283-301. doi: 10.1177/1742395313476901.
20. Tong A, Sainsbury P, Craig J. Consolidated criteria for reporting qualitative research (COREQ): a 32-item checklist for interviews and focus groups. *Int J Qual Health Care* 2007;19:349–57.doi:10.1093/intqhc/mzm042
21. Thorne S, Kirkham SR, MacDonald-Emes, J. Interpretive description: a noncategorical qualitative alternative for developing nursing knowledge. *Res Nurs Health*1997;20(2):169–177.
22. Thorne S, Kirkham SR, O'Flynn-Magee K. The analytic challenge in interpretive description. *Int J Qual Methods*2008;3(1):1-11.
23. Green J, Thorogood N. Qualitative methods for health research. London: Sage 2009.
24. Sandelowski M. Sample size in qualitative research. *Res Nurs Health*1995;18:179–183. doi: 10.1002/nur.4770180211
25. Airaksinen O, Brox JI, Cedrassch C, et al. European guidelines for the management of non-specific chronic low back pain. *Eur Spine J*2006;15 (2):s192-300. DOI:10.1007/s00586-006-1072-1
26. Van Tulder MW, Furlan A, Bombadier C, et al. Updated method guidelines for systematic reviews in the Cochrane Collaboration Back Review Group. *Spine(Phila Pa 1976)*2003;28(12):1290–9. DOI:10.1097/01.BRS.0000065484.95996.AF
27. Slade SC, Keating JL. Trunk-strengthening exercises for chronic low back pain: a systematic review. *J Manipulative Physiol Ther*2006;29(2):163-73. DOI: 10.1016/j.jmpt.2005.12.011
28. Guest G, Bunce A, Johnson L. How many interviews are enough? An experiment with data saturation and variability. *Field methods*2006;18(1):59-82.
29. Loera GM. Experiencing the barriers of nursing research. *Gastroenterol*2006;29(3):251-53.
30. Buck D, Gamble C, Dudley L, et al. From plans to actions in patient and public involvement: qualitative study of documented plans and the accounts of researchers and patients sampled from a cohort of clinical trials. *BMJ Open*2014;4(12):e006400. dx.doi.org/10.1136/bmjopen-2014-006400.
31. Linton SJ, Nicholas M, MacDonald S. Development of a short form of the Orebro Musculoskeletal Pain Screening Questionnaire. *Spine(Phila Pa 1976)* 2011;36:1891–5.doi: 10.1097/BRS.0b013e3181f8f775.
32. Fairbank JC, Pynsent PB. The Oswestry Disability Index. *Spine(Phila Pa 1976)*2000;25(22):2940-2952.
33. Braun V, Clark V. Using thematic analysis in psychology. *Qual Res Psychol*2006;3(2),77–101.
34. Bazeley P. Qualitative data analysis: Practical strategies. London: Sage 2013.
35. Norris N. Error, bias and validity in qualitative research. *Educational Action*

For peer review only - http://bmjopen.bmj.com/site/about/guidelines.xhtml

Research 1997;5:172–6.

36. Bunzli S, Watkins R, Smith A, et al. Lives on hold: A qualitative synthesis exploring the experience of chronic low-back pain. *Clin J Pain* 2013;29(10):907-16. doi: 10.1097/AJP.0b013e31827a6dd8.
37. Daykin A, Richardson B. Physiotherapists' pain beliefs and their influence on the management of patients with chronic low back pain. *Spine(Phila Pa 1976)* 2004;29(7):783-95. doi: 10.1097/01.BRS.0000115135.19082.97.
38. Linton S, Boersma K, Jannson M, et al. A randomised controlled trial of exposure in vivo for patients with spinal pain reporting fear of work-related activities. *Eur J Pain* 2007;12:722-30. DOI:10.1016/j.ejpain.2007.11.001.
39. Bunzli S, Smith A, Watkins R, et al. What Do People who score highly on the Tampa Scale of Kinesiophobia really believe? A mixed methods investigation in people with chronic non-specific low back pain. *Clin J Pain* 2015;31(7):621-32. doi: 10.1097/AJP.0000000000000143.
40. Shavers VL, Bakos A, Sheppard VB. Race, ethnicity, and pain among the US adult population. *J Health Care Poor Underserved* 2010;21(1):177-220. doi: 10.1353/hpu.0.0255.
41. Wertli MM, Burgstaller JM, Weiser S, et al. Influence of catastrophizing on treatment outcome in patients with nonspecific low back pain: a systematic review. *Spine(Phila Pa 1976)* 2014;39(3):263-73. doi:10.1097/BRS.0000000000000504.
42. Bunzli S, McEvoy S, Dankaerts W, et al. Patient perspectives on participation in Cognitive Functional Therapy for chronic low back pain: A qualitative study. *Phys Ther* 2016;96(9):1397-407. doi: 10.2522/ptj.20140570.
43. Rogers A, Allison T. What if my back breaks?: Making sense of musculoskeletal pain among South Asian and African–Caribbean people in the North West of England. *J Psychosom Res* 2004;57(1):79-87. doi: dx.doi.org/10.1016/S0022-3999(03)00570-1.
44. Samanta A, Samanta J, Johnson, MR, et al. Rheumatoid arthritis in minority ethnic groups: patterns of disease, clinical and sociocultural features among British South Asians. *Diversity in Health and Social Care* 2005;2(2):99-118. URI: http://hdl.handle.net/2086/1315 (accessed Sept 2017).
45. Froud R, Patterson S, Eldridge S, et al. A systematic review and meta-synthesis of the impact of low back pain on people's lives. *BMC Musculoskelet Disord* 2014;15(1):50. doi: 10.1186/1471-2474-15-50.
46. Linton SJ. A review of psychological risk factors in back and neck pain. *Spine(Phila Pa 1976)* 2000;25(9):1148-1156.
47. Main CJ, Foster N, Buchbinder R. How important are back pain beliefs and expectations for satisfactory recovery from back pain? ***Best** Pract Res Clin Rheumatol* 2010;24(2):205-217. doi: 10.1016/j.berh.2009.12.012.
48. Toye F, Barker K. "Could I be imagining this?"—the dialectic struggles of people with persistent unexplained back pain. *Disabil Rehabil* 2010;32:1722–1732. doi:10.3109/09638281003657857.
49. Scott W, Trost Z, Bernier E, et al. Anger differentially mediates the relationship between perceived injustice and chronic pain outcomes. *Pain2013*;154(9):1691-8. doi: 10.1016/j.**pain**.2013.05.015.
50. Sullivan MJ, Lynch ME, Clark AJ. Dimensions of catastrophic thinking associated with pain experience and disability in patients with neuropathic pain conditions. *Pain* 2005;113(3):310-15. doi:10.1016/j.pain.2004.11.003.
51. Walker J, Sofaer B, Holloway I. The experience of chronic back pain: accounts of loss

For peer review only - http://bmjopen.bmj.com/site/about/guidelines.xhtml

in those seeking help from pain clinics. *Eur J Pain*2006;10(3):199–207. doi: 10.1016/j.ejpain.2005.03.007.

52. Crowe M, Whitehead L, Gagan MJ, et al. Listening to the body and talking to myself–the impact of chronic lower back pain: a qualitative study. *Int J Nurs Stud*2010;47(5):586-92. doi: 10.1016/j.ijnurstu.2009.09.012.
53. Salathé CR, Melloh M, Mannion AF, et al. Resources for preventing sickness absence due to low back pain. *Occup Med*2012;*62*(4):273-280. doi.org/10.1093/occmed/kqs024.
54. Ferreira PH, Ferreira ML, Maher CG, et al. The therapeutic alliance between clinicians and patients predicts outcome in chronic low back pain. *Phys Ther2013*;93(4):470-78. doi:10.2522/ptj.20120137.
55. Staiger TO, Jarvik JG, Deyo RA, et al. Patient–physician agreement as a predictor of outcomes in patients with back pain. *J Gen Intern Med*2005;20:935–7. doi: 10.1111/j.1525-1497.2005.0175.
56. Potter M, Gordon S, Hamer P. The physiotherapy experience in private practice: the patients' perspective. *Aust J Physiother*2003;49(3):195-202.
57. Toye F, Barker K. I can't see any reason for stopping doing anything, but I might have to do it differently - restoring hope to patients with persistent non-specific low back pain - a qualitative study. *Disabil Rehabil*2012;34(11):894-903. doi:10.3109/09638288.2011.626483.
58. Synnott A, O'Keeffe M, Bunzli S, et al. Physiotherapists may stigmatise or feel unprepared to treat people with low back pain and psychosocial factors that influence recovery: a systematic review. *J Physiother*2015;61(2):68-76. doi: 10.1016/j.jphys.2015.02.016.
59. Slade SC, Molloy E, Keating JL. 'Listen to me, tell me': a qualitative study of partnership in care for people with non-specific chronic low back pain. *Clin Rehabil*2009;23: 270–80. doi: 10.1177/0269215508100468.
60. Jeffrey JE, Foster NE. A qualitative investigation of physical therapists' experiences and feelings of managing patients with nonspecific low back pain. *Phys Ther*2012;92(2):266-78. doi: 10.2522/ptj.20100416.
61. Greenhalgh T, Helman C, Chowdhury AM. Health beliefs and folk models of diabetes in British Bangladeshis: a qualitative study. *BMJ*1998;316(7136):978-83.
62. Allison TR, Symmons DP, Brammah T, et al. Musculoskeletal pain is more generalised among people from ethnic minorities than among white people in Greater Manchester. *Ann Rheum* Dis2002;61(2):151-6.
63. Klaber Moffett JA, Newbronner E, Waddell G, et al. Public perceptions about low back pain and its management: a gap between expectations and reality? *Health Expect2000*;3(3):161-68.
64. May SJ. Patient satisfaction with management of back pain. Part 2: An explorative, qualitative study into patients' satisfaction with physiotherapy. *Physiotherapy*2001; 87:10–20. dOI: http://dx.doi.org/10.1016/S0031-9406(05)61186-8
65. Costa LD, Maher CG, McAuley JH, et al. Self-efficacy is more important than fear of movement in mediating the relationship between pain and disability in chronic low back pain. *Eur J Pain*2011;15(2):213-9. doi: 10.1016/j.ejpain.2010.06.014
66. Fersum VK, O'Sullivan P, Skouen JS, et al. Efficacy of classification-based cognitive functional therapy in patients with non-specific chronic low back pain: A randomized controlled trial. *Eur J Pain*2013;*17*(6):916-928. doi: 10.1002/j.1532-2149.2012.00252.x

For peer review only - http://bmjopen.bmj.com/site/about/guidelines.xhtml

67. Parsons S, Harding G, Breen A, et al. The influence of patients' and primary care practitioners' beliefs and expectations about chronic musculoskeletal pain on the process of care: a systematic review of qualitative studies. *Clin J Pain*2007;23(1): 91–98. DOI: 10.1097/01.ajp.0000210947.34676.34
68. Peacock S, Patel S. Cultural influences on pain. *Rev Pain*2008;1(2):6–9.doi: 10.1177/204946370800100203.
69. Bernstein IA, Malik Q, Carville S, et al. Low back pain and sciatica: summary of NICE guidance. *BMJ* (Online)2017;356. doi: https://doi.org/10.1136/bmj.i6748 (Published 06 January 2017).
70. Nicholas MK, Linton SJ, Watson PJ, et al. Early identification and management of psychological risk factors ('yellow flags') in patients with low back pain: a reappraisal. *Phys Ther* 2011;91:737–53 doi.org/10.2522/ptj.20100224.

For peer review only

For peer review only - http://bmjopen.bmj.com/site/about/guidelines.xhtml

Supplementary File 2

1st Topic guide

Notes: The topic guide will be a flexible tool which is open to revision as new areas of interest arise during the process of data collection. In order to limit the length of the interviews, it is not essential that each interview should include every line of questioning as detailed below. Depth of exploration of fruitful areas of discussion is more important than complete coverage of all areas in every interview.

Introduction to the interview:

The interviewer will reassure the person being interviewed that:

- The research study is interested in finding out about their beliefs and experiences in living with CLBP
- There is no intention of 'testing' the interviewees about how well they are managing their CLBP.
- That they are free to say as much or as little as they wish in response to any line of questioning.
- That the content of the interview will not be divulged to their Doctor or Physiotherapist.

Background information:

In order to be able to describe the sample of patients who contribute to the study, the interviewer will invite the person being interviewed to provide information about:

- Ethnicity - participant to define their ethnicity
- Age
- Occupation
- When their LBP first started
- Previous treatment

Interview guide with prompts:

Opening

A. History of illness and health seeking behaviour

1) When did it start to impact on your daily life?

2) Who have seen regarding your CLBP problem?

Probe:

- Have you had any Investigations?

For peer review only

Prompts i.e MRI/ x-ray

- What did they show?
- What treatment (s) have you had in the past for your LBP?
- How **helpful** were these **treatments?**

B. Coping strategies

3) How do you cope with your CLBP at present?

Probe:

- What strategies do you have to manage your CLBP?
- What do you do when you have a flare up of pain?
- How do those around you cope/help (i.e. family or friends)?
- Does anyone in your family suffer with CLBP? How do they cope?

  How well do you think you manage your CLBP?

C. Cause of condition

4) What have you been told about the **cause** of your CLBP?
   - How did this make you feel?

5) What are your thoughts about the cause of your CLBP? Why do you think this?

D. CLBP beliefs

6) Have you come across any other views or beliefs related to the cause of CLBP amongst people you know?

Probe:

- Where do you think they come from?
- Is that what you think yourself?

E. Alternative treatments

7) Some people use alternative treatments e.g. acupuncture/reiki/reflexology for LBP,
8) What are your thoughts on these types of treatment?
9) Is this something you have used or would consider using?
   a. Why?
   b. Why not?

For peer review only

Probe:

- Benefits
- Disadvantages

F. CLBP and physical activity

10) What are your thoughts about people with back pain doing **physical tasks/jobs**?

Probe:

- Is bending/ lifting safe to do?
- Is there anything you avoid doing because of your CLBP?

G. Living with CLBP

11) What do you think is the effect of CLBP on your mood?

12) How much of your focus and attention does your CLBP take?

13) What do you feel will be the **best way to treat** your CLBP from now?

Probe:

- What type of treatment?

14) How do you see the **future** with regards to your CLBP?

Probe:

- How hopeful are you about the future?
- Do you feel your LBP will get better?

Ending questions

15) Is there anything else that you would like to say that we may not have covered already or discussed?

Thank you for co-operation and for taking the time to answer all the questions.

After 3rd interview Revised Topic guide

Background information:

In order to be able to describe the sample of patients who contribute to the study, the interviewer will invite the person being interviewed to provide information about:

- Ethnicity - participant to define their ethnicity
- Age

For peer review only

- Occupation
- PMH
- DH
- Activity levels /exercise
- Sleep hygiene

Interview guide with prompts:

Opening

A. History of illness and health seeking behaviour

Could you tell me the story about your LBP?

Probe (if it is not clear from the narrative question)

- When it started, how it started?
- When did it start to impact on your life?
- How did this affect you?

B. CLBP experiences and beliefs

Childhood/Family history of LBP/pain

Can you recall any early life experiences of LBP /pain?

- What was your experience?
- Were you told anything about it at the time? By whom?
- How did you cope with it?
- How did your family/friends react /respond towards your LBP/pain?
- What was the attitude of your parents towards your LBP/pain?

While you were growing up, did your parents/family member(s) suffer with LBP/pain?

- What was their experience?
- How did it impact on them?
- What was the cause of their pain?
- How did they try to cope with it?
- Did it have any impact on you and your family?
- Do you feel these experiences of pain have affected the way you think about your LBP?

Who have you been to see regarding your LBP?

- What was your experience?
- Did you have any examinations or tests? i.e. MRI/ x-ray
- What did they show?
- How was this explained to you?
- Was the language used simple, or did it include medical terms?
- What was your understanding of this?

For peer review only

- How did this make you feel?

What have you been told about the cause of your LBP?

- By whom?
- How was this explained to you?
- Was the language used simple, or did it include medical terms?
- What was your understanding of this?
- How did this make you feel?

What are your thoughts about the cause of your LBP?

- What makes you think this?
- What things have influenced your thoughts?
    - Family, culture, pain experiences, interactions with HCPs?

What do you feel people around you think causes LBP?

- Where do you think these ideas come from?

What treatment(s) have you had in the past for your LBP?

- What was your experience of these treatments?
- Did they match what you thought would be best for your LBP? If not, why not?
- Did these treatments influence your:
    - Control over your LBP?
    - Ability to do things you wanted to do?
    - Confidence regarding your back?

How do you cope with your LBP now?

- If you had a flare up, how would you manage it? Why?
- How would you manage an acute ankle or knee sprain?
- Would this be the same or different to a LBP flare up? If not, why?
- How do those around you respond or react to you regarding your LBP (i.e. family or friends)?

C. <u>HCP relationships</u>

How do you feel HCP's have interacted with you regarding your LBP?

- What was your experience of the time they spent?
- How well did they listening to you?
- Did they build your confidence?

For peer review only

Do you feel your cultural background influences your relationship with HCP's and the way you have been treated?

- What are your experiences?
- Have you experienced any differences or inequalities due to your cultural background?

D. <u>Views on exercise and physical activity</u>

What are your thoughts about people with LBP doing physical exercise?

- Why?
- Is there anything you should avoid doing because of your LBP? Why?
- What are views on people with LBP bending/ lifting? Why?

E. <u>Alternative treatments</u>

Many people use alternative treatments e.g. acupuncture/reiki/reflexology for LBP

What are your thoughts on these types of treatments? Why?

What do you think are the best treatments for LBP? Why?

F. <u>Living with CLBP</u>

In your experience has LBP impacted on your:

- Health? Activity? Social life? Work? General health? Comorbidities? Other pain areas? Sleep? Mood?
- How?
- How much do you think about your back? Why?

How do you picture yourself in 5 years with regards to your CLBP?

- How hopeful are you about the future?
- Do you feel your LBP will get better? Why?

<u>Ending questions</u>

Is there anything else that you would like to say that we may not have covered already or discussed?

Thank you for co-operation and for taking the time to answer all the questions.

For peer review only

Supplementary File 3

Audit Trail example

Interview transcript exerts and comments

The GS's comments on interviews are illustrated below,

Transcript S1, lines 1019-1042

I: Right, that's interesting. So with regards to bending and lifting, what are your thoughts on that?

P: I make sure that…. when I am lifting something or I have to bend down, I make absolutely sure that I am bending my knees and not just tipping over or whatever

I: Ok

P: You know, your manual handling and all that comes in….so you start thinking, the last thing I want to do is injure myself further or cause any more discomfort

I: Ok

P: So you are bending your knees and making sure you are lifting it….**GS's thoughts** Impact of manual handling training/Impact of pain on function – bending/lifting - Adopted cautious/protective behvoiur to bending /lifting -Psychological impact vigilance/catstrophising

I: And how long have you been doing that for?

P: Well, years now

I: So before your back started hurting?

P: O yeah

I: Then has that changed at all since your back?

P: No….I think a bit more….conscious. Consciously, it's almost like if you have….when I say you…, if I have a task that it would probably involve lifting a box or whatever, I will now consciously think, make sure you bend your knees and you keep your back straight if you're lifting something or whatever. Whereas previously you think you are fine, it doesn't matter, nothing is going to happen so…. **GS's thoughts** Psychological impact of pain – hypervigilance/ catastrophising with lifting having - Adopted manual handling behavoiur to protect back from future pain - Increased threat to back

I: So your thought process around your lifting has changed?

P: It has changed, yes. It will change

I: Ok. So what thoughts are going through your mind then when you see something heavy on the floor?

P: Making sure that you don't end up causing more damage **GS's thoughts** Coping startgeies- Atuuidtde to pain -Biomedical belief -Protective behavoiur/Caution

Supplementary File 4

Table: Theme 1 examples

Theme	Subtheme	Code	Example quotes from participants
**Biomedical back pain beliefs**	*Subtheme 1.1: Cause of CLBP attributed to physical (structural/anatomical) factors*	Back strain with bending	"I went pick a pair of socks up from the floor so and as I went to pick it up as I was getting back up there was just like this crack sounding noise and erm I think that was my first experience of it." [S10]
		Back strain with lifting	"It was definitely related, there was a timber and I went to pick it up and I felt my back hurt" [S2]
		Specific traumatic event	"I was involved in a car accident where my car, at a roundabout junction, was hit from the back and I think because of the angle I was standing with my neck turned, the impact was quite severe" [S1 PM MD MPR]. "I had a complicated pregnancy in 1995. Ahh, with my son, it was a very difficult birth; really prolonged labour and ammm significant problems during the actual birth. That was quite a shock, needless to say. I didn't have any epidural or anything like that when I had him so my body went in to shock I think with the experience ….I started to get it…." [S4 WBF MD MPR]
		Biomedical self diagnosis	"It could be a trapped nerve, a slipped disc" [S1 PM MD MPR] "…I suppose I'd always think it gets to the point where they cant actually find anything wrong so…its wear and tear because of your age, because there's 50 odd years of…and its almost something which I accept. You almost expect it because you see old people who are struggling with joints and this sort of thing and you think well yeah but for 80 odd years those knees have worked so I can understand a bit of it" [S8 WM LD LPR]
		Biomedical Back Pain beliefs in the Family and cultural community	"My mother in law just says its lifting and stuff" [S3 WBF MD MPR]. "They think that by lifting heavy weights or something or moving something heavy that that does their back in"[S7 PM MD MPR]
	Subtheme 1.2: Recalling HCP biomedical diagnosis and the biomedical beliefs	Biomedical diagnosis	"They thought it was sciatica"[S5 PF HD HPR] "The doctors have told me that I have got sacralisation"[S2 WBM MD HPR]
		Biomedical diagnosis via MRI	[Recalling MRI diagnosis] "It ermm showed like a bulging…ermm at the lower…spine but it was down to my age" [S9 WBF MD HPR] "I had an M.R.I. scan which was showing that I had got wear and tear on the vertebrates and that L4 and L5 were closing up" [S3 WBF MD

For peer review only

	adopted	Manual handling influence: protect spine with lifting and bending<br><br>Nurse occupational belief	MPR].<br>" I suppose if you lift incorrectly it will cause back pain…. I mean we have all had it drummed in to us, how to bend our knees and how to do all that malarkey" [S2 WBM MD HPR]<br>"Well that's what you get on all these health and safety things at work and lifting weights, they, they reckon you're supposed to bend at the knees, keep your back straight and stand with your legs" [S8 WM LD LPR]<br>"I was a nurse for many many years on a very heavy surgical ward and I wonder if maybe I have damaged it while nursing, you kind of associate nursing with back pain, well I do anyway."[S2 WBM MD HPR]
	Subtheme 1.3: Vulnerability of the spine	Central to function<br><br>Can't see the back<br><br>Feeling weak and cautious<br><br>Precious<br><br>Needs urgent attention<br><br>Fear avoidant beliefs	"I rely on it well most people rely on their back"[S6 PM LD LPR]<br>"Your spine in general is…central to you ….. I think you're reminded of it more each day" [S8 WM LD LPR]<br>"Because your back's...behind you, you can't see what's going on inside it or outside it."[S10 PF HD HPR]<br>"It's always been slightly weaker well felt slightly weaker well it felt slightly weaker so I was just a bit more cautious of it"[S6 PM LD LPR]<br>"I've always had a bit of a weakness in my back for quite a few years" [S9 WBF MD HPR]<br>"My back seems a bit more precious" [S6 PM LD LPR]<br>"I feel like my back's more precious rather than my wrist or ankle because it is a vital piece" [S5 PF HD HPR]<br>"So if you know there is a problem, go and fix it….the thing is if you don't fix it…then the impact of it…so the old English saying 'a stitch in time saves nine" [S1 PM MD MPR].<br>"Do try and get it sorted out at the most earliest point. Don't leave it" [S3 WBF MD MPR].<br>"Obviously not lifting really heavy stuff"[S5 PF HD HPR]<br>"…Only pick up something that you feel you could pick up, it's not too heavy." [S10 PF HD HPR]
	Subtheme 1.4: Future outlook	Positivity<br><br>Pessimism	"I even think now that I will wake up and it will all go away and it maybe will." [S2 WBM MD HPR]<br>"Well hopefully in a little less pain than I am in right now" [S3 WBF MD MPR]<br>"I think it's always going to hurt me and it's always going to cause me pain" [S5 PF HD HPR]<br>"When I look into the future I just think how am I gonna cope with a child when I can't yknow

For peer review only

		Uncertainty<br><br>Religious beliefs interlinked with outlook on pain	carry heavy things" [S10 PF HD HPR)<br>"I can only play it by ear" [S8 WM LD LPR]<br>"But I'm not sure….having lived with it for this long, I'm not sure" [S3 WBF MD MPR]<br>[Referring to finger amputation] "As I would say, someone up there wanted them so they were gone …..my sin plate clean on that day and we start again sort of thing" [S1 PM MD MPR]

Table: Theme 2 examples

Theme	Subtheme	Code	Quote from participants
**Coping with CLBP**	*Subtheme 2.1: Active coping strategies*	Self-searching for knowledge and understanding<br><br>Learning from pain experience<br><br>Confronting pain and battling on<br><br>Acceptance and adapting to pain, due to a lack of understanding, ineffective interventions and pain duration	"Ammm, it was just something off the internet that suggested you stretch and what not…"[S3 WBF MD MPR]<br>"You have got to look at why it is hurting and you can ask yourself that question. Don't expect anyone else to have all the answers" [S4 WBF MD MPR]<br>"With me neck it certainly helps being..active, being yknow rather than sitting staring at a screen" [S8 WM LD LPR]<br>"All I was bothered about was getting things better for him (disabled child) because he literally couldn't do anything" [S4 WBF MD MPR]<br>"I just managed it and got on with it..I tend to be quite stubborn" [S3 WBF MD MPR]<br>"I have kind of giving up looking for an answer. I just think whatever happens I have just got to deal with it" [S5 PF HD HPR]<br>"Put up with it because basically nothing seemed to work" [S3 WBF MD MPR]<br>"It became normal to me because I've had that for so long. It got normal for me to me feel little niggles in my back"[S6 PM LD LPR]
	Subtheme 2.2: Reliance on HCP's and biomedical interventions	Unrelenting search for understanding answers<br><br>Explanation & reassurance via investigation	"I wonder what it is and for the children ….. I would like to know" [S3 WBF MD MPR]<br>"I'm looking for answers but I want those answers. I am a dog with a bone" [S4 WBF MD MPR]<br>"Well I wanted a second scan" [S3 WBF MD MPR]<br>"An X-ray, MRI scan or anything just so I could have some reassurance that it's nothing major" [S10 PF HD HPR]

For peer review only - http://bmjopen.bmj.com/site/about/guidelines.xhtml

		Reliance on medication<br><br><br><br><br><br><br>Punjabi reliance on Biomedical Quick fix	"I basically lived on painkillers ever since" [S3 WBF MD MPR]<br>"Well if it does get severe to the point where I can actually feel it…… That's the day I would probably go home and have a couple of paracetamol or something" [S1 PM MD MPR]<br>"Manipulation yeah …… I just wanted to get the back fixed"[S7 PM MD MPR]<br>" I have a massage every 4-6 weeks. Am… and acupuncture" [S5 PF HD HPR]<br>"I have had new chairs…..I got the one with the lumbar support and an incline" [S1 PM MD MPR]
	Subtheme 2.3: Protective and avoidance coping strategies	Adopted caution, protect spine<br><br><br><br><br><br><br><br><br><br><br><br><br><br><br><br><br><br><br>Avoidance behaviour: exercise, physical task	"If you keep bending at the back all the time. The back takes the strain for everything. If you're bending at the knee to access what it is you need to get it takes the pressure out of your back from a moving and handling perspective" [S4 WBF MD MPR]<br>"I can stay in the office and look after my back …… whereas previously I might think, o well I might go for a walk around the building ……I wouldn't be doing that because it wouldn't be enjoyable and it would hurt" [S2 WBM MD HPR]<br>"Subconsciously you know that you should move a certain way to avoid injury. You know that, well I do because it is part of how I live yeah" [S4 WBF MD MPR]<br>"I can't do this my back hurts, or I can't do that my back hurts" [S10 PF HD HPR]<br>"Yeah I have stopped swimming" [S4 WBF MD MPR]<br>"I used to be very active, I just don't do any exercise really at the minute" [S9 WBF MD HPR]<br>"If I bend over, that's how my back went, and it's more than once, it's twice now, anytime I go to bend down now I think maybe it's going to go again or I shouldn't be doing this" [S6 PM LD LPR]<br>"I don't do any heavy lifting" [S2 WBM MD HPR]<br>"On my drive now I've got a load of bricks and if I need to lift them up ….. ten years ago I wouldn't of thought nothing about it I would of picked them ….. whereas now maybe.... I think really haven't got it in me to lift them ….. I feel a bit weaker so it's more, now it's become if I can avoid it I avoid it . I just think why take the risk on that"[S6 PM LD LPR]
	Subtheme 2.4: Coping	Punjabis passive to active	"I felt like I was doing more instead of sitting in my bed all day" [S5 PF HD HPR]

For peer review only

	transition	coping style	"Over time I tried to not spend too much time lying down and just tried to keep myself active as possible …… just try to keep things moving" [S7 PM MD MPR]<br>"I've had to like buy books and stuff myself and do research on the internet myself to ermm be able to look into these things more" [S10 PF HD HPR]
	Subtheme 2.5: Health benefits of an active lifestyle in Punjabis	Psychological, emotional effect	"I try and do other things now to…like maybe go to the cinema and like sit in the big seats and like try and make myself a bit comfortable and like..because I've had it for so long I've gotten used to like erm making plans with my back pain so erm I'm not as depressed as I would've been maybe 5-10 years ago" [S10 PF HD HPR]

Table: Theme 3 examples

Theme	Subtheme	Code	Quote from participants
**The psychological and emotional impact of CLBP**	*Subtheme 3.1: Psychological and emotional consequences*	Depressed mood	"I wake up grumpy. I mean obviously for a person who has been able to sleep 7,8,9 hours when you are constantly waking up….even the small break in a sleep" [S1 PM MD MPR]<br>"I've never felt as low as I have done" [S9 WBF MD HPR]<br>"The injections didn't work. I kind of felt low"[S7 PM MD MPR]<br>"Sometimes I still feel really bad, really low mood because I can't do what I used "[S5 PF HD HPR]<br>"I am awful. I am moody a lot of the time…. it does make me grumpy" [S3 WBF MD MPR]
		Hopelessness	"Not much I can do about it is basically what the guy said" [S4 WBF MD MPR]<br>"There was no other route to go down" [S3 WBF MD MPR]<br>"I went and saw a chiropractor… he gave an x-ray looking for back symptoms …they said it didn't show anything, there was nothing they could do" [S7 PM MD MPR]<br>"I had an MRI scan and that's when they done and my spine was fine I was just annoyed that there was no answers" [S5 PF HD HPR]
		Frustration	"It's a ball ache and it's getting on my

		Catastrophising thoughts<br><br>Uncertainty<br><br>Lack of control over pain	nerves" [S4 WBF MD MPR]<br>"It's so frustrating where, when…sighs, yknow things like, picking up my sewing machine I just think well what's up with me?! It's ridiculous" [S9 WBF MD HPR]<br>"You wonder, what if? What if you can't carry on at the same level that you are" [S1 PM MD MPR]<br>"It just makes you think is this the start of something bigger for me?"[S6 PM LD LPR]<br>"They couldn't really give me an explanation really I was thinking then oh what's caused my problem now"[S7 PM MD MPR]<br>"Apprehensive, apprehensive about all those things that I have just spoken about. About what would the impact be in later life…..ahh, that's about it at the moment" [S1 PM MD MPR]<br>"But I'm not sure….having lived with it for this long, I'm not sure" [S3 WBF MD MPR]<br>"It's not improving, it's getting probably a bit more……regular" [S1 PM MD MPR]<br>"I'm just getting to the stage where I'm thinking on the days where it gets really really bad that I'm gone beyond the need of self-help" [S4 WBF MD MPR]
	Subtheme 3.2: Catastrophic nature of pain flare-ups	Catastrophic description: impact on body and mind	"I just…I got up and I had…I got up on the Monday morning ermmm and I felt like a cripple…I felt like I was folding it half, it was hard work to stand up straight"[S8 WBM LD LPR]<br>"It's horrible. It's the worst pain. It makes you cry it's that bad. the pain is ermmm you just can't, you just feel like you've lost control. You can't do anything for yourself you can't like… you can't even go to yknow the loo because you're taking so many steps to get there and every step you take you're like ow, ow, ow and it's that acute ermm it's just horrible. You just…I think you just kind of lose yourself and that's when you start getting depressed like "oh god it's happened again" [S10 PF HD HPR]
	Subtheme 3.3: Threat to self-identity	Threat to self	"I suppose it's hindering my life" [S3 WBF MD MPR]<br>"Obviously it's restricted what I can

			and can't do" [S2 WBM MD HPR]
		Disrupting personal care/daily function	"It's a small thing like bending over the sink to brush your teeth" [S4 WBF MD MPR]<br>"I got out of bed …it took me quite a while to sort of straighten up" [S8 WM LD LPR]<br>"My socks, my shoes, tying my shoes up, I can't bend down" [S9 WBF MD HPR]
		Dependency on family	"It's embarrassing sometimes and degrading because he ..has to help me put my underwear on because I cant bend down to even put my knickers on" [S9 WBF MD HPR]
		Disrupting house chores	"I get the hoover out and hoover, within 10 minutes I'm absolutely shattered and it's hurting my back" [S9 WBF MD HPR]<br>"Mowing the lawn... It's that pushing forward movement I mean it's a push mower.. I mean it takes us about an hour to mow our lawn. And I know that if I sit down after doing that, that I will be in trouble" [S3 WBF MD MPR]<br>"I can't stand at a sink for more than 10 minutes without pain." [S10 PF HD HPR]
		Barrier to recreational activities	"A fair weather golfer…. by the time I played a few holes. I could feel that there was additional pressure on the back" [S1 PM MD MPR]<br>"I can't ride my bike because it's in the leaning forward position and I'll have only gone half a mile but it causes pain in my back" [S3 WBF MD MPR]
		Sleep disruption	"Over the last year it is a genuine struggle if I woke up in the night I struggle to get back to sleep" [S4 WBF MD MPR]<br>"It's okay during the day but at night I'm really suffering"[S5 PF HD HPR]<br>"I am constantly tossing and turning ...."[S1 PM MD MPR]<br>"I sleep on my side but then….i start to ache in my hips and then I have to change sides …. I do wake up because of it and then it takes me about 20 minutes to go back to sleep" [S10 PF HD HPR].

For peer review only

Table: Theme 4 examples

Theme	Subtheme	Code	Quote from participants
**The social and cultural-religious impact of CLBP**	*Subtheme 4.1: Threat to family/friend relationships and social life*	Disrupting family roles/social activity	"If my back goes I can't do nothing, I wouldn't be able to do any housework or…ermmm no bending certainly no bending" [S10 PF HD HPR]<br>"If I'm restricted so is she [wife] in respect of doing things and going out and stuff…."[S2 WBM MD HPR]<br>"Even when I'm driving when we go over a speed bump or a pothole the intense pain that shoots up my back is amazing" [S3 WBF MD MPR]
		Elicit negative family emotions	"When I'm down everybody else feels down in the family" [S9 WBF MD HPR]
		Social disruption, isolation	"You have to stand up in a bar or evening and that just is so painful……., so I tend not to be social" [S10 PF HD HPR].
	Subtheme 4.2: Work impact	Disrupting work	"The pain constraints on work"[S1 PM MD MPR].<br>"I've also had to raise it with work because of the amount of time I sit by the computer and they've had to get me a special chair" [S10 PF HD HPR]
		Burdening colleagues	"While my job is actually fully sitting down, any activities where they have asked me to go and do outreach sessions, I have had to restrict them or send other people, which again isn't ideal but other people can deputise as necessary" [S2 WBM MD HPR]<br>"I didn't drive.,,.,,I went into the office a few times but then got a lift into the office from colleagues" [S7 PM MD MPR]
		Sickness absence	"I think I only might have had a few days off in the whole time I was there for the pain" [S3 WBF MD MPR]<br>"I'm off work I've had so much time off work"[S9 WBF MD HPR]
		Financial pressures	"I'm in the building trade without a back I can't earn no money. So that goes through your mind as well as that you're not going to get no sick pay or anything" [S6 PM LD LPR]<br>"Still go to work ermmm…because I've got still got bills to pay and I've still for me daughter to support through university and so on and so forth so there's that pressure" [S8 WM

For peer review only

		Changing role	LD LPR]<br>"Initially I suppose I thought…might as well have to look for a different type of job because of the….the physical aspect if you like" [S8 WBM LD LPR]
	Subtheme 4.3: The impact of CLBP on cultural and religious wellbeing	Religious rituals: disrupted sitting to meditate /secluding self<br><br>Disrupting female role within the home/burdening others	"I couldn't do like sit on the floor if it was too hard ..if I sat on the floor for a certain amount of time, then got up, started moving around, my legs would get pins and needles and would reach my back" [S7 PM MD MPR]<br>" [Referring to sitting in the temple] I'll just go when it's quiet yknow, ermmm do my praying and then come back out and then just go downstairs where there's chairs or.. I can just hang around somewhere else and that kind of…I feel secluded sometimes because yknow people tend to like wanna sit upstairs and I have to like go just downstairs" [S10 PF HD HPR]<br>"Because of the pain yknow like I can't help around the house as much as I would like" [S10 PF HD HPR]
	Subtheme 4.4: The response of family, friends and wider community to CLBP	Family: varying response/ support<br><br>Punjabi community: Lack of empathy/ understanding<br><br>Punjabi community: Stigmatized	"My brother……, he was just getting more and more annoyed every time because there was no answers"[S5 PF HD HPR]<br>"People were badgering me, yknow its not getting any better you ought to go …..and see"[S8 WM LD LPR]<br>"Yeah and whenever I did anything they'd say oh watch your back, you'd be carefully about, you don't want that happening again" [S9 WBF MD HPR]<br>"Everyone thinks like "oh her back her back, she just uses that as an excuse now…" [S10 PF HD HPR]<br>"Unfortunately there is always this advice from the community that oh I had a similar thing…no you didn't because you have no idea what the cause of my problem is…"[S1 PM MD MPR]<br>"I believe they're like the doctors that I've experienced. They always assume the worst first yknow" [S10 PF HD HPR]<br>"Oh there comes a fat cow, she's got back, yknow she just can't get up, or oh let's make room for her she's like she can't stand up or just that kind of response. It's not about pain it's about…oh she's too big, she can't do

For peer review only

			it cus she's too big"[S10 PF HD HPR]
	Subtheme 4.5: Recalling family experiences of CLBP	<u>Varying impact of CLBP/coping strategies</u><br><br><u>Varying HCP interactions</u>	"He [Brother] has medication to deal with that on a daily basis"[S4 WBF MD MPR]<br>"He [Dad] felt like a bit of self-dignity went because he was always self-dependent and independent, always doing his own thing"[S5 PF HD HPR]<br>"He [referring to husbands back pain] could sit but he couldn't stand and he couldn't walk…he could just barely walk from the edge of his room to the other side… to get to the kitchen and he would be bent over and he would be crippled". [S3 WBF MD MPR]<br>"He [Dad] has not let it really impact him to be honest" because he still goes to the gym, he still lift weights, just does everything. Before every now and again his back hurts a little bit. He does what he does it he hasn't changed anything"[S6 PM LD LPR]<br>"Well my mum used to have back pain and she always used to say 'oh doctors don't do anything they say they just can't do anything with it'"[S10 PF HD HPR]<br>"My dad suffered from back pain. He had a slipped disk. They said he would never walk again"[S5 PF HD HPR]<br>"She [Husbands physio] explained things; she showed us…..she showed us a back. What you should do with it, what you can do… she gave him exercises to do and how to manage it in the future better" [S3 WBF MD MPR]

Table: Theme 5 examples

Theme	Subtheme	Code	Quote from participants
**Reflecting on HCP interactions, management experience and expectations of future management**	*Subtheme 5.1: Varying quality of therapeutic alliance*	<u>Strong therapeutic alliance</u>	"I felt that it was explained pretty much…completely if you like" [S2 WBM MD HPR]<br>"I left that consultation knowing that I can physically do more than what I thought I could do"[S6 PM LD LPR]<br>"She helped me and showed me how to do it"[S5 PF HD HPR]<br>"I felt they were a lot more like approachable, they were a lot more

For peer review only

		Weak therapeutic alliance	understanding…..I was listened to, that was the biggest thing" [S10 PF HD HPR]<br>"I'm doing exercises, …. which I've been told to do"[S9 WBF MD HPR]<br>"My confidence was improving"[S5 PF HD HPR]<br>"[Referring to chiropractic] Why is that treatment not available on the NHS?" [S10 PF HD HPR]<br>"I get the feeling sometimes that it's maybe a textbook thing to treat back pain" [S4 WBF MD MPR]<br>"I think probably like my GP didn't take me seriously…. I don't think anybody like has the time" [S10 PF HD HPR].<br>"I don't think people understand the severity of the situation"[S4 WBF MD MPR]<br>"With the chiropractor there wasn't much listening ,with the back specialists there was to an extent but it wasn't too involved" [S6 PM LD LPR]<br>"I went back 5 times in total……………..So I kept going back until I got more or less what I had hoped for" [S2 WBM MD HPR]<br>"I got the impression from the Dr that when I went to see the back specialist they would do at least an ultrasound and she didn't" [S8 WM LD LPR]<br>"I was expecting a diagnosis. …. I want him to tell me" [S3 WBF MD MPR]<br>"Yeah, expected no more flare ups ….. within a matter of weeks, 2 weeks if that, I was just sitting down and had another flare up" [S7 PM MD MPR]<br>"She hadn't really done anything to my back, she hadn't really done the tests or anything"[S7 PM MD MPR]<br>"I think I was expecting to be….to kind of have something like that done to me rather than just saying, 'ok here are the exercises, do them at home" [S5 PF HD HPR]<br>"No sort of a aftercare at all you know put an ice pack on your back that's it "[S6 PM LD LPR]
	Subtheme 5.2: Interpreting the HCP explanation	Adopt caution, Protect spine<br><br>Diagnostic	"[Told by HCP] Be more careful so when you get into bed, don't just jump in"[S7 PM MD MPR]<br>"Just be careful of what I do, just be careful of how I bend, Dr's and physio" [S9 WBF MD HPR]<br>"It could be your movements, it could be

		uncertainty	your muscles, it could be disc, it could be .. anything. Ermmm but obviously…I don't know what's causing my back pain..” [S10 PF HD HPR]<br>“Mixed messages …. oh it could be your stomach, they told me that wear and tear is on my hip and that's causing pain in…. my back” [S7 PM MD MPR]
	Subtheme 5.3: Appraising interventions and ability to control CLBP	Ineffective medication, acupuncture<br><br>Short lasting manual therapies<br><br>Variable impact of injection and exercise therapies	“Some of the pain killers made no difference” [S2 WBM MD HPR]<br>“I went through the acupuncture stage and that didn't work” [S3 WBF MD MPR]<br>“I did go to see a chiropractor as well ermm because at one stage I was just like oh I can't take this anymore…. I had about 8 treatments?......I probably had about 2 or 3 treatments in one week … as soon as it stopped ermm probably about….the first 2 weeks I would say the pain was still there” [S10 PF HD HPR]<br>“They gave me some injections into my facet joints and they didn't help that much... they gave me some more injections but this time they were deeper. So since that time, I've not had any flareups as what they were before but I've had constant pain” [S7 PM MD MPR]<br>“Because I'm exercising its obviously building my muscles and whether that's causing me more pain”[S5 PF HD HPR]<br>“I went to a yoga class three weeks ago and it about killed me but I love it, the next day although there was a dull ache in my back it felt wonderful. I felt like I had really stretched it out” [S4 WBF MD MPR]<br>“My flexibility has increased”[S6 PM LD LPR]<br>“The pain goes away if I do stretching, it goes away and like arching your back over and putting your hands on your buttocks and arching back” [S3 WBF MD MPR]<br>“Exercises … I do them everyday, every morning or night… the underlying problems still there its not going away, its not getting any better” [S9 WBF MD HPR]<br>“When I was walking or running or whatever I was doing the pain was worse than it was before” [S10 PF HD HPR]<br>“My back pain got worse…it was like exercises everyday to do like my back and twisting it” [S7 PM MD MPR]

For peer review only

	Subtheme 5.4: Expectations of future management	Individualised management<br>Mind body approach<br><br><br><br><br>Effective communication: listen, give time,<br><br><br>Physical examination<br>Clear explanation and understanding of pain<br><br><br><br><br><br>Support and guidance, gain control<br><br><br><br><br><br>Increase exercise	"Almost take it case by case, person by person and work out what's best for that person"[S8 WM LD LPR]<br>"The psychological mixed with other things, it's a huge things" [S4 WBF MD MPR]<br>"I think you can help yourself a lot…. By...believing in yourself" [S8 WM LD LPR]<br>"Listening to what I have to say" [S6 PM LD LPR]<br>"They will give me enough time to explain the problem" [S2 WBM MD HPR]<br>"I think, to me its almost just a matter of communicating rather than having a rigid set routine" [S8 WM LD LPR]<br>"Give me some sort of examination of the problem area" [S2 WBM MD HPR]<br>"All I want to know is what's triggering it" [S4 WBF MD MPR]<br>"Explain why and what they're doing in detail that would be understandable, cause sometimes the physio would explain something in their term… like L1 or something, I remember hearing that"[S5 PF HD HPR]<br>"Give you the right advice and guidance"[S3 WBF MD MPR]<br>"Maybe some follow up consultations to see how have you progressed" [S6 PM LD LPR]<br>"Most importantly that they will give me some advice or treatment, exercise. Something that I can go home and feel that there is progress being made" [S2 WBM MD HPR]<br>"Exercise – yeah well I need to keep doing exercise"[S7 PM MD MPR]<br>"Just some sort of activity I can do that helps it and relieves the pain"[S9 WBF MD HPR]

For peer review only

Supplementary File 1

Consolidated criteria for reporting qualitative studies (COREQ): 32-item checklist

No. Item	Guide questions	Description	Location in manuscript (Section)
**Domain 1: Research team and reflexivity**			
Personal Characteristics			
1. Interviewer/facilitator	Which author/s conducted the interview or focus group?	GS, CN	Methods and Results
2. Credentials	What were the researcher's credentials? E.g. PhD, MD	GS – BSc<br><br>CN –MSc, BSc<br><br>KOS – PhD, MSc PG Cert HE<br><br>AS – PhD, BSc<br><br>NH - PhD, MSc, Grad Dip Phys, PG Cert HE,	Methods
3. Occupation	What was their occupation at the time of the study?	Musculoskeletal Physiotherapists	Methods
4. Gender	Was the researcher male or female?	Male	Methods
5. Experience and training	What experience or training did the researcher have?	GS/CN undertook 3 hours of NIHR training on semi-structured interviewing	Methods
Relationship with participants			
6. Relationship established	Was a relationship established prior to study commencement?	No	Methods
7. Participant knowledge of the interviewer	What did the participants know about the researcher? e.g. personal goals, reasons for doing the research	Participants were briefed on the purpose of the study and understood that it was a research project for GS.<br><br>Ethical approval had been granted, participants reviewed the participant information leaflet prior to giving their written informed consent to be involved.	Methods, Acknowledgements: footnotes (ethical approval)
8. Interviewer	What characteristics were	Potential source of bias: GS is a	Methods, Strengths

For peer review only

characteristics	reported about the inter viewer/facilitator? e.g. Bias, assumptions, reasons and interests in the research topic	British Punjabi male and CN a White British male with 11 and 13 years musculoskeletal physiotherapist experience respectively, both with special interests in CLBP disorders	limitations and implications for future research
**Domain 2: study design**			
Theoretical framework			
9. Methodological orientation and Theory	What methodological orientation was stated to underpin the study? e.g. grounded theory, discourse analysis, ethnography, phenomenology, content analysis	Interpretive description and thematic analysis	Methods
Participant selection			
10. Sampling	How were participants selected? e.g. purposive, convenience, consecutive, snowball	Purposive sampling	Methods
11. Method of approach	How were participants approached? e.g. face-to-face, telephone, mail, email	Mail – study<br><br>Information, and a consent letter for telephone contact were posted to eligible individuals with their physiotherapy appointment letter.	Methods
12. Sample size	How many participants were in the study?	Ten	Methods and Results – Table 1
13. Non-participation	How many people refused to participate or dropped out? Reasons?	Ten potential participants that were invited for a semi-structured interview, all gave informed consent and completed the interview. There were no participants who subsequently refused to participate, withdrew consent or dropped out.	Methods
Setting			
14. Setting of data collection	Where was the data collected? e.g. home, clinic, workplace	All interviews were conducted in a quiet room in the physiotherapy department.	Methods
15. Presence of non-participants	Was anyone else present besides the participants and researchers?	No	n/a
16. Description of sample	What are the important characteristics of the sample? e.g. demographic	The sample comprised of five White British (2 males: 3 females) and five English speaking Punjabi (3 males: 2 females) people, with	Methods and Results – Table 1

	data, date	a mean age of 40 years. Data was collected between April 2014 and April 2015.	
Data collection			
17. Interview guide	Were questions, prompts, guides provided by the authors? Was it pilot tested?	Interviews were semi-structured using a topic guide (Supplementary File 3) informed by contemporary CLBP literature, and research team expertise. Further refinements were made following two pilot interviews with CLBP patients.	Methods, Supplementary File 3
18. Repeat interviews	Were repeat inter views carried out? If yes, how many?	No	n/a
19. Audio/visual recording	Did the research use audio or visual recording to collect the data?	The interviews were audio-recorded	Methods
20. Field notes	Were field notes made during and/or after the inter view or focus group?	No	n/a
21. Duration	What was the duration of the interviews or focus group?	Interviews lasted between 60-70 minutes.	Methods
22. Data saturation	Was data saturation discussed?	Recruitment continued until data saturation was met for the purpose of the study.	Methods
23. Transcripts returned	Were transcripts returned to participants for comment and/or correction?	No	n/a
**Domain 3: analysis and findings**			
Data analysis			
24. Number of data coders	How many data coders coded the data?	GS, CN and AS independently assessed the accuracy and completeness of all the transcripts	Methods
25. Description of the coding tree	Did authors provide a description of the coding tree?	Provided in Table 3: The thematic development illustrating key stages when themes were changed.	Results – Table 3
26. Derivation of themes	Were themes identified in advance or derived from the data?	GS, CN and AS ensured themes related to the thematic development and emerging	Methods

For peer review only

		themes	
27. Software	What software, if applicable, was used to manage the data?	Microsoft word	n/a
28. Participant checking	Did participants provide feedback on the findings?	Member checking was not conducted to validate interview transcripts due to time and funding issues.	Strengths limitations and implications for future research
Reporting			
29. Quotations presented	Were participant quotations presented to illustrate the themes/findings? Was each quotation identified? e.g. participant number	Yes, specific comments were supported with direct quotes derived from each participant. Quotations were identified using anonymised participant codes.	Results and Supplementary file 5
30. Data and findings consistent	Was there consistency between the data presented and the findings?	Yes	n/a
31. Clarity of major themes	Were major themes clearly presented in the findings?	Yes	n/a
32. Clarity of minor themes	Is there a description of diverse cases or discussion of minor themes?	Yes	Discussion

For peer review only

BMJ Open

Exploring the Lived Experience And Chronic Low Back Pain Beliefs Of English Speaking Punjabi And White British People: a qualitative study within the NHS

Journal:	*BMJ Open*
Manuscript ID	bmjopen-2017-020108.R1
Article Type:	Research
Date Submitted by the Author:	14-Dec-2017
Complete List of Authors:	Singh, Gurpreet; University of Birmingham, School of Sport, Exercise and Rehabilitation Sciences; University Hospitals of Leicester NHS Trust<br>Newton, Christopher; University Hospitals of Leicester NHS Trust<br>O'Sullivan, Kieran; Aspetar Qatar Orthopaedic and Sports Medicine Hospital, Sports Spine Centre<br>Soundy, Andrew; University of Birmingham,<br>Heneghan, Nicola; University of Birmingham, Centre of Precision Rehabilitation for Spinal Pain, School of Sport, Exercise and Rehabilitation Sciences
**Primary Subject Heading**:	Rehabilitation medicine
Secondary Subject Heading:	Patient-centred medicine
Keywords:	low back pain, lived experiences, cultural beliefs

SCHOLARONE™ Manuscripts

For peer review only - http://bmjopen.bmj.com/site/about/guidelines.xhtml

Title

Exploring the Lived Experience And Chronic Low Back Pain Beliefs Of English Speaking Punjabi And White British People: A qualitative study within the NHS

Authors

Gurpreet Singh[1, 2], Christopher Newton [1], Kieran O'Sullivan [3, 4], Andrew Soundy [2], Nicola R Heneghan [5]

Affiliations

1 University Hospitals of Leicester NHS Trust, Leicester, United Kingdom

2 School of Sport, Exercise and Rehabilitation Sciences, University of Birmingham, Birmingham, United Kingdom

3 Department of Clinical Therapies, University of Limerick, Limerick, Ireland

4 Sports Spine Centre, Aspetar Orthopaedic and Sports Medicine Hospital, Doha, Qatar

5 Centre of Precision Rehabilitation for Spinal Pain, School of Sport, Exercise and Rehabilitation Sciences, University of Birmingham, Birmingham, United Kingdom

Correspondence:

Nicola R Heneghan

Centre of Precision Rehabilitation for Spinal Pain (CPR Spine),
School of Sport, Exercise & Rehabilitation Sciences
University of Birmingham, Edgbaston,
Birmingham. B15 2TT. UK
Email: n.heneghan@bham.ac.uk

Tel: +44 121 415 836

For peer review only

For peer review only - http://bmjopen.bmj.com/site/about/guidelines.xhtml

Abstract

Introduction: Disabling chronic low back pain (CLBP) is associated with negative beliefs and behaviours, which are influenced by culture, religion and interactions with healthcare practitioners (HCPs). In the United Kingdom (UK) HCPs encounter people from different cultures and ethnic backgrounds, with South Asian Indian's (including Punjabis) forming the largest ethnic minority group. Better understanding of the beliefs and experiences of ethnic minorities with CLBP might inform effective management.

Objectives: To explore the CLBP beliefs and experiences of English-speaking Punjabi and White British people living with CLBP, explore how beliefs may influence the lived experience of CLBP and conduct cross-cultural comparisons between the two groups.

Design: Qualitative study using semi structured interviews, set within an interpretive description framework and thematic analysis.

Setting: An NHS Hospital Physiotherapy department, Leicester, UK.

Participants: 10 CLBP participants (5 English-speaking Punjabi and 5 White British) purposively recruited from Physiotherapy waiting lists.

Results: Participants from both groups held negative biomedical CLBP beliefs such as the 'spine is weak'; experienced unfulfilling interactions with HCP's commonly due to a perceived lack of support, and negative psychosocial dimensions of CLBP with most participants catastrophising about their CLBP. Specific findings to Punjabi participants included (i) disruption to cultural-religious wellbeing, as well as (ii) a perceived lack of understanding and empathy regarding their CLBP from the Punjabi community. In contrast to their White British counterparts', Punjabi participants reported initially utilising passive coping strategies, however all participants reported a transition towards active coping strategies.

Conclusion: CLBP beliefs and experiences, irrespective of ethnicity, were primarily biomedically-orientated. However, cross-cultural differences included cultural-religious well-

For peer review only - http://bmjopen.bmj.com/site/about/guidelines.xhtml

being, the community response to CLBP experienced by Punjabi participants, and coping styles. These findings might help inform management of people with CLBP.

Strengths and limitations of this study

- The first study to provide a cross-cultural exploration of CLBP beliefs and experiences of English-speaking Punjabi and White British people living with CLBP.
- Using purposive sampling, 1:1 semi-structured interviews were conducted through a cultural lens to explore beliefs and experiences of Punjabi and White British people with CLBP.
- The study findings were data-driven and embedded in the participants' voice.
- Participants were all English-speaking and were only selected from one geographical location, which may limit the transferability of the findings.
- Member checking was not conducted to validate interview transcripts.

For peer review only

For peer review only - http://bmjopen.bmj.com/site/about/guidelines.xhtml

Introduction

Chronic low back pain (CLBP) is the primary cause of disability and financial burden to healthcare and society in the United Kingdom (UK).[1] Despite increasing resources spent to improve CLBP management, the associated disability continues to rise.[2]

Historically, the impact of CLBP on developing populations was perceived to be minimal, but is now understood to be similar to western populations.[3-8] In the UK a higher incidence of spinal pain has been reported in South Asians, [9] and a higher prevalence of widespread musculoskeletal pain reported in South Asian Indian Punjabis (22%) compared with Europeans (9%).[10]

Biomedical beliefs about CLBP are common within western populations and are emergent within developing populations.[3 11 12] Factors thought to influence these beliefs include ethnicity, religion, family and friends, as well as unhelpful interactions with healthcare practitioners (HCPs).[13 14]

Current CLBP research has largely focused on western societies with little emphasis on minority ethnic populations; a concern given ethnic migration and cultural diversity is increasing within western societies. According to the last UK Census (2011)[15] Leicester hosts the largest Indian (referring to themselves as Asian or British Asian) population (30%) of any local authority in England and Wales.

Understanding cultural variations in pain perception, beliefs, expectations, and behaviours is important to accurately identify patients' needs and behaviours relative to one's own potentially divergent culture.[16] This may help avoid health inequalities and suboptimal outcomes,[17 18] an important consideration for healthcare policy makers and those

For peer review only - http://bmjopen.bmj.com/site/about/guidelines.xhtml

responsible for service provision. To tailor management, HCPs might benefit from understanding an individual's beliefs and experiences of CLBP within the cultural context in which they occur.[7 19] Therefore, research is required to understand the management of CLBP within different populations within the UK. Cross-cultural comparative studies utilising qualitative methodologies may provide in-depth understanding of individual and culture specific beliefs and experiences of CLBP. However, the authors are not aware that any such comparisons have been made between English speaking Punjabi and White British CLBP populations

Briefly, Punjabi people are characterised by migrating from the traditional area of the Punjab region in India or Pakistan, or having relatives that had done so. They may speak the Punjabi language and share values, customs and beliefs, identifying with Sikhism, Hinduism or Islam.

Therefore, this study aimed to investigate the beliefs and experiences of people living with CLBP in English speaking Punjabi and White British populations. The objectives were to explore how these beliefs influence, and impact on, the experience of living with CLBP and identify similarities and differences between the two ethnic groups.

For peer review only - http://bmjopen.bmj.com/site/about/guidelines.xhtml

Methodology

Using semi structured interviews this study followed the COnsolidated criteria for REporting Qualitative studies (COREQ) (Supplementary File 1).[20] Interpretative description (ID) was chosen as this qualitative approach has been specifically developed for healthcare enquiries of a clinical phenomenon, using subjective accounts, for the purpose of informing clinical understanding.[21] Prior theoretical and clinical knowledge is valued as a starting point for research in ID, although this can be challenged and developed as the research progresses.[22]

Sample

Purposive sampling[23] was employed to recruit White British and Punjabi participants sufficient to enable relevant data to be obtained and analysed.[24] The study setting was Leicester (UK), which hosts a large White British and Punjabi population.

Eligible participants were White British and English speaking Punjabi people aged 18-65 years with CLBP of ≥ 6-month duration.[25] Individuals with a previous history of surgery for CLBP, diagnosed with a specific or a serious underlying cause of their CLBP (i.e. fracture, infection, inflammatory spondyloarthropathy, cancer, or nerve root compression)[26 27] or who had previous physiotherapy treatment from the authors (GS, CN) were excluded.

Recruitment

Potential participants were identified by GS following a GP or Consultant referral to an NHS Physiotherapy department for CLBP between April 2014 and April 2015. Study information was posted to eligible individuals with their physiotherapy appointment letter. Following this, an interview was arranged via telephone, prior to physiotherapy commencing. GS obtained written informed consent pre-interview. Recruitment continued until saturation was achieved [28], where robust common themes were established which included knowledge that could

For peer review only - http://bmjopen.bmj.com/site/about/guidelines.xhtml

also be applied back to and illustrate the individual cases which were identified.[21] All participants were eligible and included. The sample comprised of five White British (2 males: 3 females) and five English speaking Punjabi (3 males: 2 females) people, with a mean age of 40 years (Table 1). All Punjabi participants were English speaking, third generation, UK born citizens.

Data collection

In-depth semi-structured interviews were conducted by GS (British Punjabi male) or CN (White British male), with 11 and 13 years musculoskeletal physiotherapy experience respectively, with a special interest and *a-priori* knowledge of CLBP. Both authors undertook three hours of National Institute of Health Research training on semi-structured interviewing.[29] No prior relationship was established with participants, following the interview all participants commenced physiotherapy treatment with physiotherapists that were not involved with this study. Interviews took place in a quiet room in the physiotherapy department and lasted between 60-70 minutes.

A topic guide was informed by contemporary CLBP literature,[7 13] and research team expertise. This informed the basis and boundary of focus moving forward to analysis in accordance with interpretive description[21]. Further refinements were made following two pilot interviews with CLBP patients.[30] The topic guide included open-ended questions related to the individual's 'story of their CLBP', their beliefs about causation, management and the future) as well as the lived experience of CLBP (related to interaction with HCPs, coping with CLBP and its personal, psychological, social and cultural impact) (Supplementary File 2).

Participants provided demographic data and completed validated questionnaires for pain severity (Numerical Pain Rating Scale was a sub-item score from the Short Form Orebro Musculoskeletal Questionnaire (SFOQ)),[31] functional disability (Oswestry Disability Index)[32]

For peer review only - http://bmjopen.bmj.com/site/about/guidelines.xhtml

and psychosocial risk profile (including sleep, anxiety, depression, catastrophising, fear-activity and fear-work were sub-item scores from the SFOQ)[31] (Tables 1 and 2).

Data analysis

Interviews were audio-recorded and transcribed verbatim by GS who analysed the data using thematic analysis.[33] GS considered each script repeatedly as a way to immerse himself in the data before coding began[21]. Each transcript was analysed line-by-line using an iterative model immediately after the first interview. This involved: data sampling, collection and analysis occurring in tandem as an on-going constant comparative process[34] to facilitate the capture of emergent themes during data collection.[33] This process allowed active engagement and familiarisation with the data. However some of the terms for beliefs and coping were identified from previous literature.[7 13] These terms represented critical analysis and recontexulisation of knowledge from which the analysis could be shaped [21]. From this, initial themes were generated and data-driven coding facilitated the development of a thematic table, which was modified as data analysis and interpretation evolved (Supplementary File 3). Crucially this involved critique by the co-authors (CN, KOS, AS and NH) [35] to enhance rigour and trustworthiness of study findings.[23] GS, CN and AS independently assessed the accuracy and completeness of all the transcripts, ensuring these related to the thematic development and emerging themes; this process was collated as an audit trail (Supplementary File 4). Data collection and analysis was transparent and detailed.

For peer review only - http://bmjopen.bmj.com/site/about/guidelines.xhtml

Results

Five main themes emerged from the interviews: i) biomedical back pain beliefs, ii) coping with CLBP, iii) the psychological and emotional dimensions of living with CLBP, iv) the social and cultural-religious impact of CLBP and v) reflecting on HCP interactions, management experience and expectations of future management. These themes are presented in a compare/contrast style between the ethnic groups. Due to the commonalities between the two groups, the findings presented apply to both groups unless otherwise stated (Supplementary File 5).

Table 1: Participant Characteristics

Participant code	CLBP duration (years)	Age (years)	Sex	Ethnicity
S1	25	40	Male	Punjabi
S2	2	51	Male	White British
S3	18	35	Female	White British
S4	20	42	Female	White British
S5	2	23	Female	Punjabi
S6	7	37	Male	Punjabi
S7	4	40	Male	Punjabi
S8	1	53	Male	White British
S9	2	49	Female	White British
S10	14	38	Female	Punjabi

For peer review only - http://bmjopen.bmj.com/site/about/guidelines.xhtml

Table 2: Participant pain, disability and psychosocial risk profile data

Participant code	NPRS	ODI (%)	SFOQ	Sleep	Anxiety	Depression	Catastrophising	Fear-Activity	Fear-Work	Employment Status
S1	7	(Moderate) 28	(Moderate) 48	4	5	4	7	7	3	Working
S2	8	(Moderate) 38	(High) 59	1	6	5	10	5	5	Working
S3	5	(Moderate) 24	(Moderate) 46	6	0	0	5	10	5	Working
S4	8	(Moderate) 30	(Moderate) 48	5	7	2	10	2	2	Retired
S5	3	(High) 51	(High) 50	7	6	7	5	0	1	Off work
S6	2	(Low) 14	(Low) 22	2	0	0	3	4	3	Working
S7	5	(Moderate) 24	(Moderate) 41	6	3	2	8	7	1	Working
S8	2	(Low) 14	(Low) 29	2	3	1	3	8	3	Working
S9	4	(Moderate) 40	(High) 73	7	8	9	8	9	3	Working
S10	8	(High) 50	(High) 77	7	1	6	10	10	7	Working

ODI = Oswestry Disability Index, SFOQ = Short Form Orebro musculoskeletal pain questionnaire, NPRS = Numeric Pain Rating Scale

For peer review only - http://bmjopen.bmj.com/site/about/guidelines.xhtml

Theme 1: Biomedical back pain beliefs

Cause of CLBP attributed to physical and structural/anatomical factors

All participants held similar biomedical CLBP beliefs. Common causal beliefs attributed CLBP to physical and structural/anatomical factors. These mainly included bending and lifting strains, for example:

> *"I basically bent down to pick up a pen or something and it clicked and I couldn't straighten myself up"* [S6]

Consistent with these beliefs, in cases where a physical causal mechanism could not be recalled, participants self-diagnosed a structural/anatomical cause for their CLBP. The most frequently expressed labels included: 'slipped disc' (n=5), 'wear and tear' (n=3) and 'trapped nerve' (n=3).

Recalling HCPs biomedical diagnosis and the biomedical beliefs adopted

Most participants recalled a diagnostic label derived from HCPs embedded within the biomedical model, consistent with their own beliefs. Nonetheless, some interpreted this information negatively. Following a consultation with a chiropractor, one participant perceived his back *"...was out of place"* [S7]

Biomedical CLBP beliefs were influenced by manual-handling training and by participants' occupation. A HCP working in a hospital believed the repetitive nature of manual handling in ward settings to be a cause of his CLBP. Subsequently, participants' adopted the belief their spine needed protecting when bending and lifting:

> "I suppose if you lift incorrectly it will cause back pain. I mean we have all had it drummed in to us (manual-handling training), how to bend our knees and how to do all that malarkey" [S2]

Vulnerability of the spine

Many viewed their spine as vulnerable, central to function and critical to one's well-being. Given participants' had 'one back', that was hard to see, combined with a feeling of weakness, the back was commonly described as 'precious', and when compared to an ankle sprain required more protection:

For peer review only - http://bmjopen.bmj.com/site/about/guidelines.xhtml

> *"It just feels as though the spine controls so much of your function in your legs and everything, that if you injure it, it's much more serious than perhaps injuring your ankle*" [S2]

Consistent with higher levels of disability, participants' fear avoidant beliefs suggested they believed pain indicated harm, and was a warning signal from the back:

> "*your back is trying to tell you something. It's trying to tell you to stop doing whatever you're doing if it's hurting…because you are making it worse."* [S3]

<u>*Future outlook*</u>

Participants tried to maintain a positive future outlook; this was mainly based on prior pain experiences, and individual personalities.

> "I even think now that I will wake up and it will all go away and it maybe will." [S2]

Despite this, there was an overriding feeling of uncertainty:

> "*I don't want to think about that because I don't know how I'm going to be*" [S5]

For some Punjabi participants' religion was expressed as a key part of their lives and interlinked with their positive outlook on pain. One participant described a hand injury whereby his fingers were amputated *as 'God's will'* and expressed his positive outlook in the context of his religious beliefs:

> "Someone up there wanted them so they were gone...my sin plate was wiped clean on that day and we start again." [S1]

Theme 2: Coping with CLBP

<u>*Active coping strategies*</u>

White British participants' predominantly demonstrated active coping 'self-help' strategies based on prior CLBP experiences and searching online for knowledge and understanding:

> *"I went onto NHS choices and typed in sciatica - just that word and a whole load of stuff comes up and there is one big sentence and it says 'keep active…."* [S2]

Amongst the White British group, exercise strategies such as stretching, yoga and football were utilised. They commonly shared the narrative 'confronting pain and battling on'.

For peer review only - http://bmjopen.bmj.com/site/about/guidelines.xhtml

Influenced by a need to avoid interference in one's life, fulfill family and parental duties and to avoid burdening others, this often meant enduring pain.

"I even think now that I will wake up and it will all go away and it maybe will." [S2]

Reliance on HCPs and biomedical interventions

A lack of understanding and control over pain led some participants to rely on HCPs in an unrelenting search for a diagnostic label, whilst others sought reassurance via scans:

"I wanted to have a scan just to see if there was anything major ermmm major wrong" [S9]

Both groups expressed varying levels of reliance on medication, ranging from daily use, to situations of desperation where they felt 'physically stuck' or to prevent pain intensifying.

In contrast to White British participants, Punjabi participants' demonstrated a passive reliance on HCPs to provide 'quick fix" interventions including acupuncture, massage and most frequently manual therapies:

"you go there (manual therapist) for a quick fix" [S6]

Protective and avoidance coping strategies

In terms of bending, lifting and heavy physical tasks, many participants used protective and avoidance strategies. Meanwhile, experiencing exercise-related pain, increased fear avoidance around exercise, which promoted resting behaviours. Uncertainty about the cause of pain increased hypervigilance to the threat of pain and adherence to manual handling advice to protect the back reflecting a belief of spinal vulnerability:

"I think a bit more…. if I have a task that it would probably involve lifting a box or whatever, I will now consciously think, make sure you bend your knees and you keep your back straight if you're lifting something or whatever. Whereas previously you think you are fine, it doesn't matter, nothing is going to happen so…" [S1]

Coping transition

An interesting difference emerged in the coping trajectories of White British and Punjabi participants. Although, it was not clear how this transition took place, all Punjabi participants' reported a transition from a passive reliance on HCPs for a "quick fix" to more active coping

For peer review only - http://bmjopen.bmj.com/site/about/guidelines.xhtml

strategies such as self-searching the internet, as well as replacing rest with increased physical and social activities in order to resume normal life:

"Instead of relax it…. I'd just go back to my normal routine" [S6].

Theme 3: The psychological and emotional dimensions of CLBP

Psychological and emotional consequences

Across both groups, a number of negative psychological and emotional dimensions of CLBP were expressed. Those with more disabling CLBP most frequently reported depressed mood, hopelessness, frustration, catastrophising thoughts, and uncertainty about pain and lack of control over it.

Depressed mood was most often expressed in relation to the persistence of CLBP, and as a consequence of failed interventions, disruption to sleep and engagement in meaningful activities. Some attributed disabling CLBP to weight gain resulting in depressed mood:

"because I put on a lot of weight when I was bed bound that got me really down" [S5]

Hopelessness was attributed to a lack of control over pain and a lack of support from HCPs, who in some cases painted a pessimistic outlook:

"No I mean they just said it's just down to wear and tear basically and you've got to live with it" [S9]

Frustration was repeatedly expressed in relation to a lack of understanding of the cause of pain, and the interference with daily life, and in some cases the desire to exercise and lose weight. Participants with more disabling pain held catastrophic CLBP thoughts. Similar to depressed mood and frustration, this was associated with diagnostic uncertainty and a lack of control over pain, resulting in catastrophising about the future:

"I might not walk again" [S5]

Catastrophic nature of pain flare-ups

Often participants' used catastrophic language to portray the unpredictable, fluctuating and uncontrollable disabling impact of flare-ups, for example:

For peer review only - http://bmjopen.bmj.com/site/about/guidelines.xhtml

"I would say at it's very worst point it feels like a ball of fire. It's debilitating to the point where I want to cut my left-side off. It's awful and the longer the pain continues the worse it seems to spread" [S4]

For some this entailed a physical and emotional struggle. However only a few seemed to form a link between their thoughts and CLBP:

"When I have got a project or something... I am not thinking about my back at all. I am just cracking on. I'm noticing it more because I have got time on my hands" [S4]

<u>*Threat to self-identity*</u>

Cross-cultural comparisons highlighted that CLBP posed a major threat to participants' *'self' and their identity. S*ome described negative consequences of CLBP such as low self-esteem, reduced independence and disempowerment. One participant expressed disabling CLBP as a major loss:

"Everything is just gone now like dignity, confidence. I had a stick but I would only use that sometimes if I was going out" [S5]

During flare-ups, a small number expressed difficulties carrying out daily functional activities. The greatest impact seemed to be on bending-related tasks such as putting on socks and hoovering. This led to dependency on family members and changing roles in their relationships, which negatively impacted participants' psychological and emotional well-being.

Theme 4: The social and cultural-religious impact of CLBP

<u>*Threat to family/friend relationships and social life*</u>

Mostly expressed by White British participants', the interference of CLBP on family relationships and fulfilling parental roles was described as 'restrictive' on their spouse/partner, often eliciting negative emotions in family members. One participant identified her coping strategies as a potential cause of frustration:

"My husband also gets frustrated with me. He says 'what you being a legend for, why don't you just sit down and do it later'. I don't know… he still thinks I have OCD" [S4]

For peer review only - http://bmjopen.bmj.com/site/about/guidelines.xhtml

CLBP inhibited social interactions across both groups. Social isolation was the consequence for some previously sociable participants' with more disabling pain:

> *"Just walking round town I have to stop and go in a café and have a coffee just to sit down to rest my back. I stay at home now"* [S9]

Work impact

Disrupting work roles, those sitting at work constantly fidgeted and had move in an attempt to control pain. Participants also commented how CLBP impacted on colleagues. Sickness absence was discussed by most with varied views, some cited having time off work due to a flare-up:

> *"I did have time off 'cos my back was hurting too much"* [S7]

The impact of CLBP on cultural and religious wellbeing

CLBP negatively impacted cultural-religious wellbeing of Punjabi participants, consistently impeding meditation, particularly as this involved sitting cross-legged on a floor for long periods:

> *"I do sit down crossed legs on the floor when we pray and meditate and maybe that has slight impact on it, you know when it gets just maybe it gets tired"* [S6]

This impact varied from 'the hips and back locking', to an inability to sit leaving one participant secluding herself from religious rituals that involved sitting:

> "[Referring to sitting in the temple] I'll just go when it's quiet y'know, ermmm do my praying and then come back out and then just go downstairs where there's chairs or.. I can just hang around somewhere else and that kind of…I feel excluded sometimes because y'know people tend to like wanna sit upstairs and I have to like go just downstairs" [S10]

Cultural roles and obligations were impacted. In this context, one Punjabi female described the 'perceived' female cultural role and how CLBP disrupted her ability to carry out household duties including cooking. Viewed as essential to the female role amongst Punjabi's, difficulties with or an inability to carry out these duties had far-reaching consequences such as finding a marriage partner:

For peer review only - http://bmjopen.bmj.com/site/about/guidelines.xhtml

"With the Asian culture a girl has to do housework - she has to get prepared for her married life, so she has to learn how to cook, she has to learn how to do housework, look after her husband and when you can't do that, you're you know not suitable anymore" [S10]

The response of family, friends and wider community to CLBP

Participants' reported experiencing varied responses from friends and family members to their CLBP. These included feeling pushed to seek healthcare to 'get it fixed', feeling supported in some cases, and over supported in others. In contrast, one Punjabi participant experienced very little empathy and support:

"My family don't take me seriously anymore because they're just sick of hearing about it and my friends just don't understand" [S10]

Some Punjabi participants' felt stigmatized, with cultural comparisons indicating a perceived lack of empathy and understanding from people within the Punjabi community:

"In other cultures do kind of ... they tend to be a bit more understanding" [S10]

CLBP advice from Punjabi community members, the self-acclaimed 'back pain experts', left one participant frustrated:

"*Unfortunately we live in a community that everyone thinks they are a qualified doctor. You know, don't do this, do this sort of a thing*" [S1]

Recalling family experiences of CLBP

Participants recalled back pain experiences of family members with regards to their coping strategies, levels of disability and interactions with HCPs. Some family members reluctantly relied on medication, whilst others adopted active coping strategies and demonstrated self-efficacy. However, participants did not consistently adopt the coping strategies observed. For example, one Punjabi participant initially utilised passive interventions (including massage) in contrast to his father:

For peer review only - http://bmjopen.bmj.com/site/about/guidelines.xhtml

> *"He has not let it really impact him to be honest because he still goes to the gym, he still lift weights, just does everything. Before every now and again his back hurts a little bit. He does what he does - he hasn't changed anything"* [S6]

Theme 5: Reflecting on HCP interactions, management experience and expectations of future management

Varying quality of therapeutic alliance

Reflecting on previous HCP interactions, many experienced variations in therapeutic alliance, with mostly negative interactions. Strong therapeutic alliance was associated with HCPs providing clear communication. This included a clear explanation about the cause of pain, as well as reassurance, collaborative compassionate care with on-going support and guidance. This increased adherence to treatment, built empowerment and trust in their HCPs:

> "*I've great faith in the physio*" [S2]

In contrast, several factors were attributed to weak therapeutic alliance. Some depicted a power struggle, where the HCP was in control, *and* access to investigations such as MRI scans was rejected, others reported a lack of individualised holistic care. However, most cited HCPs communication as a major problem, particularly not being given time, not being taken seriously, not feeling understood nor listened to. One participant reported feeling disrespected:

> *"It really did upset me when I went to see a consultant - I felt very belittled by how he approached me"* [S9]

Expectations were often unmet, where participants did not receive investigations, a clear diagnosis, a physical examination or manual therapy whilst enduring unexpected pain flare-ups. A perceived lack of guidance and support from HCPs was also cited, in some cases resulting in feelings of helplessness and low mood.

For peer review only - http://bmjopen.bmj.com/site/about/guidelines.xhtml

Interpreting the HCP explanation

Iatrogenic language used by HCPs was fear-inducing for some participants. Interpretations of HCPs explanations led to a sense of vulnerability around the spine and a need to adopt caution. One participant in his thirties recalled his interpretation of 'wear and tear':

> *"It makes you think that something you've got something permanent and you're basically going to have to live with it"* [S6]

However, uncertainty about the actual cause of their pain was most common, partly due to mixed messages conveyed by HCPs and inconclusive radiological investigations.

Appraising interventions and ability to control CLBP

Reflecting on prior CLBP management, this was embedded within the biomedical model for the majority. Treatment interventions were appraised in relation to their therapeutic effect and the participant's ability to control their pain. Medications, despite being taken for long periods were deemed largely ineffective by many, as was acupuncture. In addition, a reliance on manual therapies offered minimal long-term effect:

> *"If I'm honest - at the time it's a psychological plaster"* [S4]

Expectations of future management

Interestingly, when exploring cross-cultural expectations of future management, all participants' sought an individualised, mind-body approach, which involved a physical 'hands on' examination. Many expressed the need for HCPs to possess strong communication skills which include: empathy, active listening skills, providing time, clear communication and to:

> *"Explain things thoroughly, don't frighten the patient, and just generally be welcoming"* [S3]

For peer review only - http://bmjopen.bmj.com/site/about/guidelines.xhtml

Discussion

This is the first study to examine the lived experience and CLBP beliefs of English speaking Punjabi and White British people. Our findings suggest several between-group similarities amongst most participants including biomedical back pain beliefs, unfulfilling HCP interactions and negative psychological emotional and social influences of CLBP. Differences included CLBP disrupting Punjabi participants' participation in cultural-religious activities. One Punjabi participant reported CLBP disrupted her 'perceived female role' within the home. Many Punjabi participants also experienced a lack of empathy and understanding from the Punjabi community. Whilst White British participants adopted active coping strategies, all of their Punjabi counterparts initially reported a preference for passive coping strategies, but all reported a transition to active coping strategies.

Biomedical beliefs

Biomedical CLBP beliefs were common amongst all participants, their family, friends and particularly the wider Punjabi community. This supports the view that biomedical beliefs may not be exclusive to certain populations, instead reflecting the views of western society overall.[8 36] Biomedical beliefs conveyed by HCPs were adopted by, or were similar to those already held by, participants', consistent with other studies.[13 37] These beliefs were often associated with negative CLBP information,[38] around bending and lifting, perpetuating beliefs of spinal vulnerability culminating in fear avoidance beliefs and behaviours.[39] Further, the role of cultural-religious pain beliefs in promoting a positive future outlook has been documented in other cultural groups.[40] However only one Punjabi participant expressed pain beliefs within a positive cultural-religious context, perhaps reflecting participants' predominantly biomedical beliefs.

For peer review only - http://bmjopen.bmj.com/site/about/guidelines.xhtml

Coping strategies and transition

Illustrated as an on-going challenge by many participants, coping strategies have been shown to influence the development and persistence of CLBP.[41] Most White British participants at times utilised active coping strategies such as self-searching for knowledge and exercises, thus demonstrating self-efficacy. A recent qualitative CLBP study[42] reported patients require an explanation and understanding of their CLBP, consistent with our study. Many searched relentlessly, primarily via reliance on HCPs for biomedical interventions, perhaps reflecting the importance participants attached to finding a diagnosis that would legitimise their pain.[19] Meanwhile, Punjabi participants' initial reliance on HCPs to provide passive 'quick fix' interventions and a dependency on family members may highlight their biomedical beliefs, underpinned by a lack of understanding and control over pain resulting in low self–efficacy. These coping strategies may have been influenced by interactions with family, or cultural community members, or the HCP management approach. In support, passive coping strategies have been identified in a previous UK study among a South Asian population with chronic pain.[43] However, acculturation levels were low and perhaps participants lacked knowledge about western medicine. Other UK studies in South Asian populations have identified a reliance on 'complementary' medications.[44] In contrast, in our study Punjabi participants' pursued more conventional western medications and interventions. This may reflect greater awareness of, or access to, these treatment options, given Punjabi participants were third generation UK born and likely well acculturated. A novel finding of our study is that all Punjabi participants reported a transition from passive to active coping strategies.[3 5] This may reflect the limited effects of passive interventions, as well as greater knowledge and understanding of CLBP and the potential benefits of active coping strategies. Alternatively, this group may have perceived the HCP as an authoritarian figure and complied with the HCPs approach to management even if it was not their preference.

For peer review only - http://bmjopen.bmj.com/site/about/guidelines.xhtml

Psychological and emotional dimensions

The impact of CLBP has been found to extend beyond physical domains,[3] with many negative and often life-changing psychological and emotional effects.[19 36 45] Contrary to earlier research,[46] participants did not appear to consider these factors as contributors to CLBP, instead viewing these as secondary effects of CLBP. Supporting this, one study found South Asians were unwilling to recognise the influence of psychological, emotional and social factors on their pain.[43] However, negative beliefs about the control of CLBP and the resulting passive coping often reported by participants may have a mediating influence on depressed mood, pain and disability.[47] Feelings of frustration were common amongst participants' due to a lack of explanation and understanding about CLBP, including inconclusive diagnostic radiological investigations. This may reflect their desire for answers linked with pain legitimisation and validation.[48] Contrary to previous research,[49] perceptions of 'not feeling believed' were not consistent with participants' views, nor were feelings of anger and perceived injustice associated with the negative impact of CLBP. Perhaps, these feelings did exist, but were not expressed due to fear of being judged or it negatively impacting on physiotherapy. Furthermore, our findings lend support to a study showing catastrophic thoughts were associated with a magnified threat to the 'unpredictable' and 'fluctuating' nature of pain flare-ups, excessive worry about pain and a pessimistic view of controlling pain.[50]

Social dimensions

Similar to other studies, CLBP was highly disabling, described as a 'major loss' by some. It impacted on many aspects of individual's lives including their identity, self-esteem and independence, leaving some disempowered.[45 51 52] For many, this meant their lives were 'on hold', a finding consistent with a recent systematic review.[36] Our data supports the notion that CLBP impacts meaningful relationships, threatening parental and family duties and for some resulting in social isolation.[3 5 45] With regards to work, although participants'

For peer review only - http://bmjopen.bmj.com/site/about/guidelines.xhtml

demonstrated some avoidance behaviours in the workplace, at the time of interviewing only one participant was absent from work due to CLBP. This may reflect active coping in relation to work, possibly influenced by financial concerns associated with sickness absence, good work support and job satisfaction [53] or positive HCP advice relating to work.

Therapeutic alliance and a person-centred approach

Strong therapeutic alliance in the management of CLBP has been associated with greater treatment compliance, improved clinical outcomes[54] and greater levels of patient satisfaction.[55] However, most participants' experienced weak therapeutic alliance. Associated with weak therapeutic alliance,[56] the management of CLBP for most was viewed as lacking an individualised and holistic approach. Furthermore, participants' predominantly experienced a lack of guidance and support, and poor HCP communication.[19] Examination of HCP communication revealed a lack of clear explanation and participants' understanding about pain, instead creating uncertainty for many. Language has been identified as an important facet of effective communication given it is personal and variable, particularly amongst different ethnic populations. Consistent with findings in other populations,[7] HCPs commonly used fear-inducing language, which in combination with biomedical CLBP beliefs and poor HCP communication is linked to weak therapeutic alliance and CLBP related disability.[57] These factors may reflect HCPs lack of interpersonal skills, particularly specialised communication skills, and their over-utilisation of biomedical approaches to CLBP management,[58] posing a greater challenge to managing ethnic minority populations. These findings question how HCPs interact with people living with CLBP. HCPs may require training to enhance their communication skills and partnerships with patients.[59 60]

It is also worth noting that participants' in our study did not experience inequalities in accessing care or treatment, contrary to findings in other South Asian ethnic populations,[61 62] who utilised participants' with low acculturation levels. Participants' experience of HCP interactions highlighted a biomedical approach to CLBP. This may be due to the influence of

For peer review only - http://bmjopen.bmj.com/site/about/guidelines.xhtml

HCPs biomedical CLBP beliefs on their clinical management.[13] Interestingly, most participants' sought an individualised self-management approach[59 60 63] delivered by empathetic HCPs with effective communication,[64] perhaps more aligned with the bio-psychosocial model. This quest, along with Punjabi participants' transition to seek active coping strategies, demonstrates a desire for self-efficacy, which has been linked with reduced disability.[65] HCPs biomedical preference for managing CLBP may highlight discordance with the bio-psychosocial model advocated by recent NICE guidelines,[66] and has been associated with poor adherence to treatment.[13] Thus more individualised multi-dimensional approaches to management,[67] built on effective communication facilitating strong therapeutic alliance[68] and self-management might be needed.

Cultural differences

To date, qualitative research in the UK has paid little attention to the CLBP experience through a cultural and ethnic lens. This requires consideration, given the degree to which individuals identify with their ethnic group, share beliefs and engage in culture roles, which can influence pain experiences.[40] Consistent with other ethnic minority CLBP studies,[7 11] Punjabi participants' experienced a disruption to cultural-religious wellbeing, and endured a negative response from the wider community. In this context, some novel and potentially important themes were identified. As Punjabi participants expressed, religion maybe important to people in ethnic minority populations,[40] disruption to sitting-based meditation in some cases resulted in immense frustration and isolation. In some populations the perceived view of the female role involves housework and preparing for marriage, or serving the husband.[3 11] This perception may be similar to those held within the Punjabi community. CLBP undermined the ability to carry out these duties. For one participant this created uncertainty about finding a marriage partner, and feelings of guilt and burden on other female family members, due to increased workloads placed upon them. Our findings support those in other ethnic populations where gender differences exist in the experience of CLBP.[3 5] One novel finding of our study was that Punjabi participants' perceived a lack of empathy

For peer review only - http://bmjopen.bmj.com/site/about/guidelines.xhtml

and understanding from the Punjabi community, and for some CLBP was a source of stigmatisation.[59] These factors may reflect cultural attitudes towards people with CLBP within Punjabi communities. Influencing factors may include biomedical beliefs held or limited understanding of CLBP. Alternatively stoicism and perhaps the communication and meaning of pain may differ amongst these people and playing down pain may be more acceptable than gesturing emotion.[69] Stoicism towards CLBP may have existed amongst community members possibly due to levels of acculturation, personal response to, or outlook on CLBP. Other influences may include the participants' relationship with the community members they encountered. Findings of this study illustrate the existence of cultural-religious and gender differences specific to Punjabi participants, and highlight the need to consider factors specific to the individual in the management of CLBP.[67] It is noteworthy that while the recent NICE guidelines[66] call for a bio-psychosocial approach to CLBP, there is no specific guidance on how to acknowledge or manage socio-cultural factors and beliefs. Consequently, it may be challenging for HCPs to provide individualised, culturally sensitive bio-psychosocial management for CLBP patients from different ethnic populations.

Strengths limitations and implications for future research

One of the study strengths is its relevance to clinical practice. This is the first cross-cultural study to explore CLBP beliefs and experiences in English speaking Punjabi and White British people living with CLBP. The study findings were data-driven and embedded in the participants' voice. Reflexivity was demonstrated throughout with the authors declaring how their ethnicity; novice researcher role, special clinical interest in CLBP and *a-priori* knowledge may have influenced data collection, analysis and interpretation.

Waiting for treatment may have influenced participants' response. Member checking was not conducted to validate interview transcripts due to time and funding. High acculturation levels, over-representation of Punjabi male participants and variations in socio-demographic status, may limit the transferability of the findings. Therefore, future research could consider Punjabi populations with low acculturation rates in different geographical regions.

For peer review only - http://bmjopen.bmj.com/site/about/guidelines.xhtml

Implications for practice and policy

This study contributes to existing knowledge by providing HCPs managing CLBP in White British and English-speaking Punjabi people new insights,

Which could improve CLBP management within these groups. There may be specific training needs for HCPs to better understand the multifactorial nature of CLBP, specifically the individual's beliefs and experiences within their psychosocial and cultural-religious context.[58 70] This, in addition to developing a flexible communication style that facilitates strong therapeutic alliance may help tailor management within a person-centred approach. Other HCP priorities could include disseminating evidence-based beliefs amongst patients and the public including ethnic minority populations.[7 47]

Conclusion

CLBP beliefs and experiences similar across both groups were biomedically-orientated. CLBP was associated with negative psychological and social consequences. Cross-cultural differences related to the negative impact on cultural-religious aspects of Punjabi participants' lives. Punjabi participants also reported a transition from passive to active CLBP coping strategies and experiencing a lack of empathy from Punjabi community members. HCPs should therefore adopt a culturally-sensitive approach to the management of CLBP, which considers individuals' beliefs and experiences.

For peer review only

For peer review only - http://bmjopen.bmj.com/site/about/guidelines.xhtml

Acknowledgements

Thanks to all participants who took the time to share their stories and experiences.

Footnotes

Twitter: Follow @gsingh1902, @ChrisNewtonPT, @A_Soundy, @kieranosull @HeneghanNicola

Contributor ship statement: GS together with CN, KOS, AS and NH were responsible for the conception and design of the study. GS and CN were responsible for data collection. GS was responsible for transcription, leading data analysis and initial drafting of the article. GS, CN, KOS, AS and NH contributed to analysis, interpretation and manuscript development. All authors approved final submitted manuscript.

Data sharing: Supplementary data is available in supplementary files or can be requested through the corresponding author.

Funding: GS time funded by National Institute of Health Research

Competing interests: None Declared

Ethics Approval: Ethical approval was obtained from NRES Committee, London – Riverside, Reference Number: 14/LO/0510.

For peer review only

For peer review only - http://bmjopen.bmj.com/site/about/guidelines.xhtml

References

1. Hoy D, Brooks P, Blyth F, et al. The epidemiology of low back pain. *Best Pract Res Clin Rheumatol*2010;24:769-81. doi.org/10.1016/j.berh.2010.10.002.
2. Freburger JK, Holmes GM, Agans RP, et al. The rising prevalence of chronic low back pain. *Arch Intern Med*2009;169:251-58. doi:10.1001/archinternmed.2008.543.
3. Tavafian SS, Gregory D, Montazeri A. The experience of low back pain in Iranian women: a focus group study. *Health Care Women Int*2008;29:339-48 doi.org/10.1080/07399330701876356.
4. Snelgrove S, Liossi C. An interpretative phenomenological analysis of living with chronic low back pain. *Br J Health Psychol*2009;14:735-49 doi: 10.1348/135910709X402612.
5. Lin IB, O'sullivan PB, Coffin JA, et al. 'I am absolutely shattered': The impact of chronic low back pain on Australian Aboriginal people. *Eur J Pain*2012;16:1331-41 doi:10.1002/j.1532-2149.2012.00128.x.
6. Scheermesser M, Bachmann S, Schämann A, et al. A qualitative study on the role of cultural background in patients' perspectives on rehabilitation. *BMC Musculoskelet Disord*2012;13:5.doi.org/10.1186/1471-2474-13-5.
7. Lin IB, O'Sullivan PB, Coffin JA, et al. Disabling chronic low back pain as an iatrogenic disorder: a qualitative study in Aboriginal Australians. *BMJ open* 2013;3:e002654.dx.doi.org/10.1136/bmjopen-2013-002654.
8. Darlow B, Dean S, Perry M, et al. Easy to harm, hard to heal: patient views about the back. *Spine*2015;40:842-50. doi: 10.1097/BRS.0000000000000901.
9. Webb R, Brammah T, Lunt M, et al. Prevalence and predictors of intense, chronic, and disabling neck and back pain in the UK general population. *Spine*2003; 28:1195-1202.
10. Palmer B, Macfarlane G, Afzal C, et al. Acculturation and the prevalence of pain amongst South Asian minority ethnic groups in the UK. *Rheumatology*2007;46:1009-1014. doi.org/10.1093/rheumatology/kem037.
11. Madden V, O'Sullivan P, Fisher J, et al. Our training left us unprepared-two physiotherapists' reflections after working with women with low back pain in a rural Zulu community in South Africa. *J Community Health*2013;8(2).
12. Zusman M. Belief reinforcement: one reason why costs for low back pain have not decreased. *J Multidiscip Healthc*2013;6:197. doi.org/10.2147/JMDH.S44117.
13. Darlow B, Fullen BM, Dean S, et al. The association between health care professional attitudes and beliefs and the attitudes and beliefs, clinical management, and outcomes of patients with low back pain: a systematic review. *Eur J Pain*2012;16:3-17. DOI: 10.1016/j.ejpain.2011.06.006.
14. Schulz PJ, Hartung U, Riva S. Causes, coping, and culture: a comparative survey study on representation of back pain in three Swiss language regions. *PLoS one*2013;8(11):e78029.doi.org/10.1371/journal.pone.0078029
15. Census Key Statistics for Local Authorities in England and Wales [online]. Office for National Statistics (2011) *http://www.ons.gov.uk/ons/rel/census/2011-census/key-statistics-for-local-authorities-in-england-and-wales/rpt-ethnicity.html#tab-Differences-in-ethnicity-across-local-authorities* [Accessed 20th January, 2015].

For peer review only - http://bmjopen.bmj.com/site/about/guidelines.xhtml

16. Hobara M. Beliefs about appropriate pain behavior: cross-cultural and sex differences between Japanese and Euro-Americans. *Eur J Pain*2005;9(4):389–93 doi: 10.1016/j.ejpain.2004.09.006.

17. Brockopp DY, Ryan P, Warden S. Nurses' willingness to manage the pain of specific groups of patients. *Br J Nurs*.2003;12(7):409-15 doi.org/10.12968/bjon.2003.12.7.11261
18. Nazroo J. Health and Social research in Multiethnic Societies. Oxford and New York: Routledge 2006.
19. Snelgrove S, Liossi C. Living with chronic low back pain: a metasynthesis of qualitative research. *Chronic Illn*2013;9(4):283-301. doi: 10.1177/1742395313476901.
20. Tong A, Sainsbury P, Craig J. Consolidated criteria for reporting qualitative research (COREQ): a 32-item checklist for interviews and focus groups*. Int J Qual Health Care* 2007*;*19*:*349–57.doi:10.1093/intqhc/mzm042
21. Thorne S, Kirkham SR, MacDonald-Emes, J. Interpretive description: a noncategorical qualitative alternative for developing nursing knowledge. *Res Nurs Health*1997;20(2):169–177.
22. Thorne S, Kirkham SR, O'Flynn-Magee K. The analytic challenge in interpretive description. *Int J Qual Methods*2008;3(1):1-11.
23. Green J, Thorogood N. Qualitative methods for health research. London: Sage 2009.
24. Sandelowski M. Sample size in qualitative research. *Res Nurs Health*1995;18:179–183. doi: 10.1002/nur.4770180211
25. Airaksinen O, Brox JI, Cedrassch C, et al. European guidelines for the management of non-specific chronic low back pain. *Eur Spine J*2006;15 (2):s192-300. DOI:10.1007/s00586-006-1072-1
26. Van Tulder MW, Furlan A, Bombadier C, et al. Updated method guidelines for systematic reviews in the Cochrane Collaboration Back Review Group. *Spine(Phila Pa 1976)*2003;28(12):1290–9. DOI:10.1097/01.BRS.0000065484.95996.AF
27. Slade SC, Keating JL. Trunk-strengthening exercises for chronic low back pain: a systematic review. *J Manipulative Physiol Ther*2006;29(2):163-73. DOI: 10.1016/j.jmpt.2005.12.011
28. Guest G, Bunce A, Johnson L. How many interviews are enough? An experiment with data saturation and variability. *Field methods*2006;18(1):59-82.
29. Loera GM. Experiencing the barriers of nursing research. *Gastroenterol*2006;29(3):251-53.
30. Buck D, Gamble C, Dudley L, et al. From plans to actions in patient and public involvement: qualitative study of documented plans and the accounts of researchers and patients sampled from a cohort of clinical trials. *BMJ Open*2014;4(12):e006400. dx.doi.org/10.1136/bmjopen-2014-006400.
31. Linton SJ, Nicholas M, MacDonald S. Development of a short form of the Orebro Musculoskeletal Pain Screening Questionnaire. *Spine(Phila Pa 1976)* 2011;36:1891–5.doi: 10.1097/BRS.0b013e3181f8f775.
32. Fairbank JC, Pynsent PB. The Oswestry Disability Index. *Spine(Phila Pa 1976)*2000;25(22):2940-2952.
33. Braun V, Clark V. Using thematic analysis in psychology. *Qual Res Psychol*2006;3(2),77–101.
34. Bazeley P. Qualitative data analysis: Practical strategies. London: Sage 2013.
35. Norris N. Error, bias and validity in qualitative research. *Educational Action*

For peer review only - http://bmjopen.bmj.com/site/about/guidelines.xhtml

*Research*1997;5:172–6.

36. Bunzli S, Watkins R, Smith A, et al. Lives on hold: A qualitative synthesis exploring the experience of chronic low-back pain. *Clin J Pain*2013;29(10):907-16. doi: 10.1097/AJP.0b013e31827a6dd8.
37. Daykin A, Richardson B. Physiotherapists' pain beliefs and their influence on the management of patients with chronic low back pain. *Spine(Phila Pa 1976)*2004;29(7):783-95. doi: 10.1097/01.BRS.0000115135.19082.97.
38. Linton S, Boersma K, Jannson M, et al. A randomised controlled trial of exposure in vivo for patients with spinal pain reporting fear of work-related activities. *Eur J Pain*2007;12:722-30. DOI:10.1016/j.ejpain.2007.11.001.
39. Bunzli S, Smith A, Watkins R, et al. What Do People who score highly on the Tampa Scale of Kinesiophobia really believe? A mixed methods investigation in people with chronic non-specific low back pain. *Clin J Pain*2015;31(7):621-32. doi: 10.1097/AJP.0000000000000143.
40. Shavers VL, Bakos A, Sheppard VB. Race, ethnicity, and pain among the US adult population. *J Health Care Poor Underserved*2010;21(1):177-220. doi: 10.1353/hpu.0.0255.
41. Wertli MM, Burgstaller JM, Weiser S, et al. Influence of catastrophizing on treatment outcome in patients with nonspecific low back pain: a systematic review. *Spine(Phila Pa 1976)*2014;39(3):263-73. doi:10.1097/BRS.0000000000000504.
42. Bunzli S, McEvoy S, Dankaerts W, et al. Patient perspectives on participation in Cognitive Functional Therapy for chronic low back pain: A qualitative study. *Phys Ther*2016;96(9):1397-407. doi: 10.2522/ptj.20140570.
43. Rogers A, Allison T. What if my back breaks?: Making sense of musculoskeletal pain among South Asian and African–Caribbean people in the North West of England. *J Psychosom Res*2004;57(1):79-87. doi: dx.doi.org/10.1016/S0022-3999(03)00570-1.
44. Samanta A, Samanta J, Johnson, MR, et al. Rheumatoid arthritis in minority ethnic groups: patterns of disease, clinical and sociocultural features among British South Asians. *Diversity in Health and Social Care*2005;2(2):99-118. URI**:** http://hdl.handle.net/2086/1315 (accessed Sept 2017).
45. Froud R, Patterson S, Eldridge S, et al. A systematic review and meta-synthesis of the impact of low back pain on people's lives. *BMC Musculoskelet Disord*2014;15(1):50. doi: 10.1186/1471-2474-15-50.
46. Linton SJ. A review of psychological risk factors in back and neck pain. *Spine(Phila Pa 1976)*2000;25(9):1148-1156.
47. Main CJ, Foster N, Buchbinder R. How important are back pain beliefs and expectations for satisfactory recovery from back pain? ***Best** Pract Res Clin Rheumatol*2010;24(2):205-217. doi: 10.1016/j.berh.2009.12.012.
48. Toye F, Barker K. "Could I be imagining this?"—the dialectic struggles of people with persistent unexplained back pain. *Disabil Rehabil*2010;32:1722–1732. doi:10.3109/09638281003657857.
49. Scott W, Trost Z, Bernier E, et al. Anger differentially mediates the relationship between perceived injustice and chronic pain outcomes. *Pain2013*;154(9):1691-8. doi: 10.1016/j.**pain**.2013.05.015.
50. Sullivan MJ, Lynch ME, Clark AJ. Dimensions of catastrophic thinking associated with pain experience and disability in patients with neuropathic pain conditions. *Pain*2005;113(3):310-15. doi:10.1016/j.pain.2004.11.003.
51. Walker J, Sofaer B, Holloway I. The experience of chronic back pain: accounts of loss

For peer review only - http://bmjopen.bmj.com/site/about/guidelines.xhtml

in those seeking help from pain clinics. *Eur J Pain*2006;10(3):199–207. doi: 10.1016/j.ejpain.2005.03.007.

52. Crowe M, Whitehead L, Gagan MJ, et al. Listening to the body and talking to myself– the impact of chronic lower back pain: a qualitative study. *Int J Nurs Stud*2010;47(5):586-92. doi: 10.1016/j.ijnurstu.2009.09.012.
53. Salathé CR, Melloh M, Mannion AF, et al. Resources for preventing sickness absence due to low back pain. *Occup Med*2012;*62*(4):273-280. doi.org/10.1093/occmed/kqs024.
54. Ferreira PH, Ferreira ML, Maher CG, et al. The therapeutic alliance between clinicians and patients predicts outcome in chronic low back pain. *Phys Ther2013*;93(4):470-78. doi:10.2522/ptj.20120137.
55. Staiger TO, Jarvik JG, Deyo RA, et al. Patient–physician agreement as a predictor of outcomes in patients with back pain. *J Gen Intern Med*2005;20:935–7. doi: 10.1111/j.1525-1497.2005.0175.
56. Potter M, Gordon S, Hamer P. The physiotherapy experience in private practice: the patients' perspective. *Aust J Physiother*2003;49(3):195-202.
57. Toye F, Barker K. I can't see any reason for stopping doing anything, but I might have to do it differently - restoring hope to patients with persistent non-specific low back pain - a qualitative study. *Disabil Rehabil*2012;34(11):894-903. doi:10.3109/09638288.2011.626483.
58. Synnott A, O'Keeffe M, Bunzli S, et al. Physiotherapists may stigmatise or feel unprepared to treat people with low back pain and psychosocial factors that influence recovery: a systematic review. *J Physiother*2015;61(2):68-76. doi: 10.1016/j.jphys.2015.02.016.
59. Slade SC, Molloy E, Keating JL. 'Listen to me, tell me': a qualitative study of partnership in care for people with non-specific chronic low back pain. *Clin Rehabil*2009;23: 270–80. doi: 10.1177/0269215508100468.
60. Jeffrey JE, Foster NE. A qualitative investigation of physical therapists' experiences and feelings of managing patients with nonspecific low back pain. *Phys Ther*2012;92(2):266-78. doi: 10.2522/ptj.20100416.
61. Greenhalgh T, Helman C, Chowdhury AM. Health beliefs and folk models of diabetes in British Bangladeshis: a qualitative study. *BMJ*1998;316(7136):978-83.
62. Allison TR, Symmons DP, Brammah T, et al. Musculoskeletal pain is more generalised among people from ethnic minorities than among white people in Greater Manchester. *Ann Rheum* Dis2002;61(2):151-6.
63. Klaber Moffett JA, Newbronner E, Waddell G, et al. Public perceptions about low back pain and its management: a gap between expectations and reality? *Health Expect2000*;3(3):161-68.
64. May SJ. Patient satisfaction with management of back pain. Part 2: An explorative, qualitative study into patients' satisfaction with physiotherapy. *Physiotherapy*2001; 87:10–20. dOI: http://dx.doi.org/10.1016/S0031-9406(05)61186-8
65. Costa LD, Maher CG, McAuley JH, et al. Self-efficacy is more important than fear of movement in mediating the relationship between pain and disability in chronic low back pain. *Eur J Pain*2011;15(2):213-9. doi: 10.1016/j.ejpain.2010.06.014
66. Bernstein IA, Malik Q, Carville S, et al. Low back pain and sciatica: summary of NICE guidance. *BMJ* (Online)2017;356. doi: https://doi.org/10.1136/bmj.i6748 (Published 06 January 2017).

For peer review only - http://bmjopen.bmj.com/site/about/guidelines.xhtml

67. Fersum VK, O'Sullivan P, Skouen JS, et al. Efficacy of classification-based cognitive functional therapy in patients with non-specific chronic low back pain: A randomized controlled trial. *Eur J Pain*2013;*17*(6):916-928. doi: 10.1002/j.1532-2149.2012.00252.x
68. Parsons S, Harding G, Breen A, et al. The influence of patients' and primary care practitioners' beliefs and expectations about chronic musculoskeletal pain on the process of care: a systematic review of qualitative studies. *Clin J Pain*2007;23(1): 91–98. DOI: 10.1097/01.ajp.0000210947.34676.34
69. Peacock S, Patel S. Cultural influences on pain. *Rev Pain*2008;1(2):6–9.doi: 10.1177/204946370800100203.
70. Nicholas MK, Linton SJ, Watson PJ, et al. Early identification and management of psychological risk factors ('yellow flags') in patients with low back pain: a reappraisal*. Phys Ther* 2011*;*91*:*737–53 doi.org/10.2522/ptj.20100224.

For peer review only

Supplementary File 1

Consolidated criteria for reporting qualitative studies (COREQ): 32-item checklist

No. Item	Guide questions	Description	Location in manuscript (Section)
**Domain 1: Research team and reflexivity**			
Personal Characteristics			
1. Interviewer/facilitator	Which author/s conducted the interview or focus group?	GS, CN	Methods and Results
2. Credentials	What were the researcher's credentials? E.g. PhD, MD	GS – BSc<br><br>CN –MSc, BSc<br><br>KOS – PhD, MSc PG Cert HE<br><br>AS – PhD, BSc<br><br>NH - PhD, MSc, Grad Dip Phys, PG Cert HE,	Methods
3. Occupation	What was their occupation at the time of the study?	Musculoskeletal Physiotherapists	Methods
4. Gender	Was the researcher male or female?	Male	Methods
5. Experience and training	What experience or training did the researcher have?	GS/CN undertook 3 hours of NIHR training on semi-structured interviewing	Methods
Relationship with participants			
6. Relationship established	Was a relationship established prior to study commencement?	No	Methods
7. Participant knowledge of the interviewer	What did the participants know about the researcher? e.g. personal goals, reasons for doing the research	Participants were briefed on the purpose of the study and understood that it was a research project for GS.<br><br>Ethical approval had been granted, participants reviewed the participant information leaflet prior to giving their written informed consent to be involved.	Methods, Acknowledgements: footnotes (ethical approval)
8. Interviewer	What characteristics were	Potential source of bias: GS is a	Methods, Strengths

For peer review only

characteristics	reported about the inter viewer/facilitator? e.g. Bias, assumptions, reasons and interests in the research topic	British Punjabi male and CN a White British male with 11 and 13 years musculoskeletal physiotherapist experience respectively, both with special interests in CLBP disorders	limitations and implications for future research
**Domain 2: study design**			
Theoretical framework			
9. Methodological orientation and Theory	What methodological orientation was stated to underpin the study? e.g. grounded theory, discourse analysis, ethnography, phenomenology, content analysis	Interpretive description and thematic analysis	Methods
Participant selection			
10. Sampling	How were participants selected? e.g. purposive, convenience, consecutive, snowball	Purposive sampling	Methods
11. Method of approach	How were participants approached? e.g. face-to-face, telephone, mail, email	Mail – study<br><br>Information, and a consent letter for telephone contact were posted to eligible individuals with their physiotherapy appointment letter.	Methods
12. Sample size	How many participants were in the study?	Ten	Methods and Results – Table 1
13. Non-participation	How many people refused to participate or dropped out? Reasons?	Ten potential participants that were invited for a semi-structured interview, all gave informed consent and completed the interview. There were no participants who subsequently refused to participate, withdrew consent or dropped out.	Methods
Setting			
14. Setting of data collection	Where was the data collected? e.g. home, clinic, workplace	All interviews were conducted in a quiet room in the physiotherapy department.	Methods
15. Presence of non-participants	Was anyone else present besides the participants and researchers?	No	n/a
16. Description of sample	What are the important characteristics of the sample? e.g. demographic	The sample comprised of five White British (2 males: 3 females) and five English speaking Punjabi (3 males: 2 females) people, with	Methods and Results – Table 1

For peer review only

	data, date	a mean age of 40 years. Data was collected between April 2014 and April 2015.	
Data collection			
17. Interview guide	Were questions, prompts, guides provided by the authors? Was it pilot tested?	Interviews were semi-structured using a topic guide (Supplementary File 3) informed by contemporary CLBP literature, and research team expertise. Further refinements were made following two pilot interviews with CLBP patients.	Methods, Supplementary File 3
18. Repeat interviews	Were repeat inter views carried out? If yes, how many?	No	n/a
19. Audio/visual recording	Did the research use audio or visual recording to collect the data?	The interviews were audio-recorded	Methods
20. Field notes	Were field notes made during and/or after the inter view or focus group?	No	n/a
21. Duration	What was the duration of the interviews or focus group?	Interviews lasted between 60-70 minutes.	Methods
22. Data saturation	Was data saturation discussed?	Recruitment continued until data saturation was met for the purpose of the study.	Methods
23. Transcripts returned	Were transcripts returned to participants for comment and/or correction?	No	n/a
**Domain 3: analysis and findings**			
Data analysis			
24. Number of data coders	How many data coders coded the data?	GS, CN and AS independently assessed the accuracy and completeness of all the transcripts	Methods
25. Description of the coding tree	Did authors provide a description of the coding tree?	Provided in Table 3: The thematic development illustrating key stages when themes were changed.	Results – Table 3
26. Derivation of themes	Were themes identified in advance or derived from the data?	GS, CN and AS ensured themes related to the thematic development and emerging	Methods

For peer review only

		themes	
27. Software	What software, if applicable, was used to manage the data?	Microsoft word	n/a
28. Participant checking	Did participants provide feedback on the findings?	Member checking was not conducted to validate interview transcripts due to time and funding issues.	Strengths limitations and implications for future research
Reporting			
29. Quotations presented	Were participant quotations presented to illustrate the themes/findings? Was each quotation identified? e.g. participant number	Yes, specific comments were supported with direct quotes derived from each participant. Quotations were identified using anonymised participant codes.	Results and Supplementary file 5
30. Data and findings consistent	Was there consistency between the data presented and the findings?	Yes	n/a
31. Clarity of major themes	Were major themes clearly presented in the findings?	Yes	n/a
32. Clarity of minor themes	Is there a description of diverse cases or discussion of minor themes?	Yes	Discussion

For peer review only

Supplementary File 2

1st Topic guide

Notes: The topic guide will be a flexible tool which is open to revision as new areas of interest arise during the process of data collection. In order to limit the length of the interviews, it is not essential that each interview should include every line of questioning as detailed below. Depth of exploration of fruitful areas of discussion is more important than complete coverage of all areas in every interview.

Introduction to the interview:

The interviewer will reassure the person being interviewed that:

- The research study is interested in finding out about their beliefs and experiences in living with CLBP
- There is no intention of 'testing' the interviewees about how well they are managing their CLBP.
- That they are free to say as much or as little as they wish in response to any line of questioning.
- That the content of the interview will not be divulged to their Doctor or Physiotherapist.

Background information:

In order to be able to describe the sample of patients who contribute to the study, the interviewer will invite the person being interviewed to provide information about:

- Ethnicity - participant to define their ethnicity
- Age
- Occupation
- When their LBP first started
- Previous treatment

Interview guide with prompts:

Opening

A. History of illness and health seeking behaviour

1) When did it start to impact on your daily life?

2) Who have seen regarding your CLBP problem?

Probe:

- Have you had any Investigations?

For peer review only

Prompts i.e MRI/ x-ray

- What did they show?
- What treatment (s) have you had in the past for your LBP?
- How **helpful** were these **treatments?**

B. Coping strategies

3) How do you cope with your CLBP at present?

Probe:

- What strategies do you have to manage your CLBP?
- What do you do when you have a flare up of pain?
- How do those around you cope/help (i.e. family or friends)?
- Does anyone in your family suffer with CLBP? How do they cope?

  How well do you think you manage your CLBP?

C. Cause of condition

4) What have you been told about the **cause** of your CLBP?
   - How did this make you feel?

5) What are your thoughts about the cause of your CLBP? Why do you think this?

D. CLBP beliefs

6) Have you come across any other views or beliefs related to the cause of CLBP amongst people you know?

Probe:

- Where do you think they come from?
- Is that what you think yourself?

E. Alternative treatments

7) Some people use alternative treatments e.g. acupuncture/reiki/reflexology for LBP,
8) What are your thoughts on these types of treatment?
9) Is this something you have used or would consider using?
   a. Why?
   b. Why not?

For peer review only

Probe:

- Benefits
- Disadvantages

F. CLBP and physical activity

10) What are your thoughts about people with back pain doing **physical tasks/jobs**?

Probe:

- Is bending/ lifting safe to do?
- Is there anything you avoid doing because of your CLBP?

G. Living with CLBP

11) What do you think is the effect of CLBP on your mood?

12) How much of your focus and attention does your CLBP take?

13) What do you feel will be the **best way to treat** your CLBP from now?

Probe:

- What type of treatment?

14) How do you see the **future** with regards to your CLBP?

Probe:

- How hopeful are you about the future?
- Do you feel your LBP will get better?

Ending questions

15) Is there anything else that you would like to say that we may not have covered already or discussed?

Thank you for co-operation and for taking the time to answer all the questions.

After 3rd interview Revised Topic guide

Background information:

In order to be able to describe the sample of patients who contribute to the study, the interviewer will invite the person being interviewed to provide information about:

- Ethnicity - participant to define their ethnicity
- Age

For peer review only

- Occupation
- PMH
- DH
- Activity levels /exercise
- Sleep hygiene

Interview guide with prompts:

Opening

A. History of illness and health seeking behaviour

Could you tell me the story about your LBP?

Probe (if it is not clear from the narrative question)

- When it started, how it started?
- When did it start to impact on your life?
- How did this affect you?

B. CLBP experiences and beliefs

Childhood/Family history of LBP/pain

Can you recall any early life experiences of LBP /pain?

- What was your experience?
- Were you told anything about it at the time? By whom?
- How did you cope with it?
- How did your family/friends react /respond towards your LBP/pain?
- What was the attitude of your parents towards your LBP/pain?

While you were growing up, did your parents/family member(s) suffer with LBP/pain?

- What was their experience?
- How did it impact on them?
- What was the cause of their pain?
- How did they try to cope with it?
- Did it have any impact on you and your family?
- Do you feel these experiences of pain have affected the way you think about your LBP?

Who have you been to see regarding your LBP?

- What was your experience?
- Did you have any examinations or tests? i.e. MRI/ x-ray
- What did they show?
- How was this explained to you?
- Was the language used simple, or did it include medical terms?
- What was your understanding of this?

For peer review only

- How did this make you feel?

What have you been told about the cause of your LBP?

- By whom?
- How was this explained to you?
- Was the language used simple, or did it include medical terms?
- What was your understanding of this?
- How did this make you feel?

What are your thoughts about the cause of your LBP?

- What makes you think this?
- What things have influenced your thoughts?
  - Family, culture, pain experiences, interactions with HCPs?

What do you feel people around you think causes LBP?

- Where do you think these ideas come from?

What treatment(s) have you had in the past for your LBP?

- What was your experience of these treatments?
- Did they match what you thought would be best for your LBP? If not, why not?
- Did these treatments influence your:
  - Control over your LBP?
  - Ability to do things you wanted to do?
  - Confidence regarding your back?

How do you cope with your LBP now?

- If you had a flare up, how would you manage it? Why?
- How would you manage an acute ankle or knee sprain?
- Would this be the same or different to a LBP flare up? If not, why?
- How do those around you respond or react to you regarding your LBP (i.e. family or friends)?

C. <u>HCP relationships</u>

How do you feel HCP's have interacted with you regarding your LBP?

- What was your experience of the time they spent?
- How well did they listening to you?
- Did they build your confidence?

For peer review only

Do you feel your cultural background influences your relationship with HCP's and the way you have been treated?

- What are your experiences?
- Have you experienced any differences or inequalities due to your cultural background?

D. Views on exercise and physical activity

What are your thoughts about people with LBP doing physical exercise?

- Why?
- Is there anything you should avoid doing because of your LBP? Why?
- What are views on people with LBP bending/ lifting? Why?

E. Alternative treatments

Many people use alternative treatments e.g. acupuncture/reiki/reflexology for LBP

What are your thoughts on these types of treatments? Why?

What do you think are the best treatments for LBP? Why?

F. Living with CLBP

In your experience has LBP impacted on your:

- Health? Activity? Social life? Work? General health? Comorbidities? Other pain areas? Sleep? Mood?
- How?
- How much do you think about your back? Why?

How do you picture yourself in 5 years with regards to your CLBP?

- How hopeful are you about the future?
- Do you feel your LBP will get better? Why?

Ending questions

Is there anything else that you would like to say that we may not have covered already or discussed?

Thank you for co-operation and for taking the time to answer all the questions.

For peer review only

Supplementary File 3

Table 3: The thematic development illustrating key stages

	Thematic development stage 1		Thematic development stage 2	Thematic development stage 3	Final theme stage 4
**Theme 1**	Back pain beliefs	Revised Topic guide	Back pain beliefs	Biomedical Back pain beliefs	Biomedical Back pain beliefs
**Theme 2**	Coping with CLBP		Coping with CLBP	Coping with CLBP	Coping with CLBP
**Theme 3**	Psychosocial, dimensions of pain		Impact of CLBP	Psychological, emotional and social dimensions of CLBP	The psychological and emotional dimensions of living with CLBP
**Theme 4**	Cultural impact of CLBP		Cultural impact of CLBP	Cultural impact of CLBP	The social and cultural-religious impact of CLBP
**Theme 5**	HCP interactions		HCP interactions, management experience and future expectations	HCP interactions, management experience and future expectations	Reflecting on HCP interactions, management experience and expectations of future management

CLBP = Chronic low back pain
HCP = Health care practitioner

For peer review only

Supplementary File 4

Audit Trail example

Interview transcript exerts and comments

The GS's comments on interviews are illustrated below,

Transcript S1, lines 1019-1042
I: Right, that's interesting. So with regards to bending and lifting, what are your thoughts on that?
P: I make sure that…. when I am lifting something or I have to bend down, I make absolutely sure that I am bending my knees and not just tipping over or whatever
I: Ok
P: You know, your manual handling and all that comes in….so you start thinking, the last thing I want to do is injure myself further or cause any more discomfort
I: Ok
P: So you are bending your knees and making sure you are lifting it….***GS's thoughts*** *Impact of manual handling training/Impact of pain on function – bending/lifting - Adopted cautious/protective behvoiur to bending /lifting -Psychological impact vigilance/catstrophising*
I: And how long have you been doing that for?
P: Well, years now
I: So before your back started hurting?
P: O yeah
I: Then has that changed at all since your back?
P: No….I think a bit more….conscious. Consciously, it's almost like if you have….when I say you…, if I have a task that it would probably involve lifting a box or whatever, I will now consciously think, make sure you bend your knees and you keep your back straight if you're lifting something or whatever. Whereas previously you think you are fine, it doesn't matter, nothing is going to happen so…. ***GS's thoughts*** *Psychological impact of pain – hypervigilance/ catastrophising with lifting having - Adopted manual handling behavoiur to protect back from future pain - Increased threat to back*
I: So your thought process around your lifting has changed?
P: It has changed, yes. It will change
I: Ok. So what thoughts are going through your mind then when you see something heavy on the floor?
P: Making sure that you don't end up causing more damage ***GS's thoughts*** *Coping startgeies- Atuuidtde to pain -Biomedical belief -Protective behavoiur/Caution*

For peer review only

Supplementary File 5

Table: Theme 1 examples

Theme	Subtheme	Code	Example quotes from participants
**Biomedical back pain beliefs**	*Subtheme 1.1: Cause of CLBP attributed to physical (structural/anatomical) factors*	Back strain with bending	"I went pick a pair of socks up from the floor so and as I went to pick it up as I was getting back up there was just like this crack sounding noise and erm I think that was my first experience of it." [S10]
		Back strain with lifting	"It was definitely related, there was a timber and I went to pick it up and I felt my back hurt" [S2]
		Specific traumatic event	"I was involved in a car accident where my car, at a roundabout junction, was hit from the back and I think because of the angle I was standing with my neck turned, the impact was quite severe" [S1 PM MD MPR].<br>"I had a complicated pregnancy in 1995. Ahh, with my son, it was a very difficult birth; really prolonged labour and ammm significant problems during the actual birth. That was quite a shock, needless to say. I didn't have any epidural or anything like that when I had him so my body went in to shock I think with the experience ….I started to get it…." [S4 WBF MD MPR]
		Biomedical self diagnosis	"It could be a trapped nerve, a slipped disc" [S1 PM MD MPR]<br>"…I suppose I'd always think it gets to the point where they cant actually find anything wrong so…its wear and tear because of your age, because there's 50 odd years of…and its almost something which I accept. You almost expect it because you see old people who are struggling with joints and this sort of thing and you think well yeah but for 80 odd years those knees have worked so I can understand a bit of it" [S8 WM LD LPR]
		Biomedical Back Pain beliefs in the Family and cultural community	"My mother in law just says its lifting and stuff" [S3 WBF MD MPR].<br>"They think that by lifting heavy weights or something or moving something heavy that that does their back in"[S7 PM MD MPR]
	Subtheme 1.2: Recalling HCP biomedical diagnosis and the biomedical beliefs	Biomedical diagnosis	"They thought it was sciatica"[S5 PF HD HPR]<br>"The doctors have told me that I have got sacralisation"[S2 WBM MD HPR]
		Biomedical diagnosis via MRI	[Recalling MRI diagnosis] "It ermm showed like a bulging…ermm at the lower…spine but it was down to my age" [S9 WBF MD HPR]<br>"I had an M.R.I. scan which was showing that I had got wear and tear on the vertebrates and that L4 and L5 were closing up" [S3 WBF MD

For peer review only

	adopted		MPR].
		<u>Manual handling influence: protect spine with lifting and bending</u>	" I suppose if you lift incorrectly it will cause back pain…. I mean we have all had it drummed in to us, how to bend our knees and how to do all that malarkey" [S2 WBM MD HPR]<br>"Well that's what you get on all these health and safety things at work and lifting weights, they, they reckon you're supposed to bend at the knees, keep your back straight and stand with your legs" [S8 WM LD LPR]
		<u>Nurse occupational belief</u>	"I was a nurse for many many years on a very heavy surgical ward and I wonder if maybe I have damaged it while nursing, you kind of associate nursing with back pain, well I do anyway."[S2 WBM MD HPR]
	Subtheme 1.3: Vulnerability of the spine	<u>Central to function</u>	"I rely on it well most people rely on their back"[S6 PM LD LPR]<br>"Your spine in general is…central to you ….. I think you're reminded of it more each day" [S8 WM LD LPR]
		<u>Can't see the back</u>	"Because your back's...behind you, you can't see what's going on inside it or outside it."[S10 PF HD HPR]
		<u>Feeling weak and cautious</u>	"It's always been slightly weaker well felt slightly weaker well it felt slightly weaker so I was just a bit more cautious of it"[S6 PM LD LPR]<br>"I've always had a bit of a weakness in my back for quite a few years" [S9 WBF MD HPR]
		<u>Precious</u>	"My back seems a bit more precious" [S6 PM LD LPR]<br>"I feel like my back's more precious rather than my wrist or ankle because it is a vital piece" [S5 PF HD HPR]
		<u>Needs urgent attention</u>	"So if you know there is a problem, go and fix it….the thing is if you don't fix it…then the impact of it…so the old English saying 'a stitch in time saves nine" [S1 PM MD MPR].<br>"Do try and get it sorted out at the most earliest point. Don't leave it" [S3 WBF MD MPR].
		<u>Fear avoidant beliefs</u>	"Obviously not lifting really heavy stuff"[S5 PF HD HPR]<br>"…Only pick up something that you feel you could pick up, it's not too heavy." [S10 PF HD HPR]
	Subtheme 1.4: Future outlook	<u>Positivity</u>	"I even think now that I will wake up and it will all go away and it maybe will." [S2 WBM MD HPR]<br>"Well hopefully in a little less pain than I am in right now" [S3 WBF MD MPR]
		<u>Pessimism</u>	"I think it's always going to hurt me and it's always going to cause me pain" [S5 PF HD HPR]<br>"When I look into the future I just think how am I gonna cope with a child when I can't yknow

For peer review only

		Uncertainty<br><br>Religious beliefs interlinked with outlook on pain	carry heavy things" [S10 PF HD HPR)<br>"I can only play it by ear" [S8 WM LD LPR]<br>"But I'm not sure….having lived with it for this long, I'm not sure" [S3 WBF MD MPR]<br>[Referring to finger amputation] "As I would say, someone up there wanted them so they were gone …..my sin plate clean on that day and we start again sort of thing" [S1 PM MD MPR]

Table: Theme 2 examples

Theme	Subtheme	Code	Quote from participants
**Coping with CLBP**	*Subtheme 2.1: Active coping strategies*	Self-searching for knowledge and understanding<br><br>Learning from pain experience<br><br>Confronting pain and battling on<br><br>Acceptance and adapting to pain, due to a lack of understanding, ineffective interventions and pain duration	"Ammm, it was just something off the internet that suggested you stretch and what not…"[S3 WBF MD MPR]<br>"You have got to look at why it is hurting and you can ask yourself that question. Don't expect anyone else to have all the answers" [S4 WBF MD MPR]<br>"With me neck it certainly helps being..active, being yknow rather than sitting staring at a screen" [S8 WM LD LPR]<br>"All I was bothered about was getting things better for him (disabled child) because he literally couldn't do anything" [S4 WBF MD MPR]<br>"I just managed it and got on with it..I tend to be quite stubborn" [S3 WBF MD MPR]<br>"I have kind of giving up looking for an answer. I just think whatever happens I have just got to deal with it" [S5 PF HD HPR]<br>"Put up with it because basically nothing seemed to work" [S3 WBF MD MPR]<br>"It became normal to me because I've had that for so long. It got normal for me to me feel little niggles in my back"[S6 PM LD LPR]
	Subtheme 2.2: Reliance on HCP's and biomedical interventions	Unrelenting search for understanding answers<br><br>Explanation & reassurance via investigation	"I wonder what it is and for the children ….. I would like to know" [S3 WBF MD MPR]<br>"I'm looking for answers but I want those answers. I am a dog with a bone" [S4 WBF MD MPR]<br>"Well I wanted a second scan" [S3 WBF MD MPR]<br>"An X-ray, MRI scan or anything just so I could have some reassurance that it's nothing major" [S10 PF HD HPR]

For peer review only

		Reliance on medication<br><br><br><br><br><br><br>Punjabi reliance on Biomedical Quick fix	"I basically lived on painkillers ever since" [S3 WBF MD MPR]<br>"Well if it does get severe to the point where I can actually feel it…… That's the day I would probably go home and have a couple of paracetamol or something" [S1 PM MD MPR]<br>"Manipulation yeah …… I just wanted to get the back fixed"[S7 PM MD MPR]<br>" I have a massage every 4-6 weeks. Am… and acupuncture" [S5 PF HD HPR]<br>"I have had new chairs…..I got the one with the lumbar support and an incline" [S1 PM MD MPR]
	Subtheme 2.3: Protective and avoidance coping strategies	Adopted caution, protect spine<br><br><br><br><br><br><br><br><br><br><br><br><br><br><br><br><br><br><br>Avoidance behaviour: exercise, physical task	"If you keep bending at the back all the time. The back takes the strain for everything. If you're bending at the knee to access what it is you need to get it takes the pressure out of your back from a moving and handling perspective" [S4 WBF MD MPR]<br>"I can stay in the office and look after my back …… whereas previously I might think, o well I might go for a walk around the building ……I wouldn't be doing that because it wouldn't be enjoyable and it would hurt" [S2 WBM MD HPR]<br>"Subconsciously you know that you should move a certain way to avoid injury. You know that, well I do because it is part of how I live yeah" [S4 WBF MD MPR]<br>"I can't do this my back hurts, or I can't do that my back hurts" [S10 PF HD HPR]<br>"Yeah I have stopped swimming" [S4 WBF MD MPR]<br>"I used to be very active, I just don't do any exercise really at the minute" [S9 WBF MD HPR]<br>"If I bend over, that's how my back went, and it's more than once, it's twice now, anytime I go to bend down now I think maybe it's going to go again or I shouldn't be doing this" [S6 PM LD LPR]<br>"I don't do any heavy lifting" [S2 WBM MD HPR]<br>"On my drive now I've got a load of bricks and if I need to lift them up ….. ten years ago I wouldn't of thought nothing about it I would of picked them ….. whereas now maybe.... I think really haven't got it in me to lift them ….. I feel a bit weaker so it's more, now it's become if I can avoid it I avoid it . I just think why take the risk on that"[S6 PM LD LPR]
	Subtheme 2.4: Coping	Punjabis passive to active	"I felt like I was doing more instead of sitting in my bed all day" [S5 PF HD HPR]

For peer review only

	transition	coping style	"Over time I tried to not spend too much time lying down and just tried to keep myself active as possible …… just try to keep things moving" [S7 PM MD MPR]<br>"I've had to like buy books and stuff myself and do research on the internet myself to ermm be able to look into these things more" [S10 PF HD HPR]
	Subtheme 2.5: Health benefits of an active lifestyle in Punjabis	Psychological, emotional effect	"I try and do other things now to…like maybe go to the cinema and like sit in the big seats and like try and make myself a bit comfortable and like..because I've had it for so long I've gotten used to like erm making plans with my back pain so erm I'm not as depressed as I would've been maybe 5-10 years ago" [S10 PF HD HPR]

Table: Theme 3 examples

Theme	Subtheme	Code	Quote from participants
**The psychological and emotional impact of CLBP**	*Subtheme 3.1: Psychological and emotional consequences*	Depressed mood<br><br><br><br><br><br><br><br><br><br><br><br><br><br><br>Hopelessness<br><br><br><br><br><br><br><br><br><br>Frustration	"I wake up grumpy. I mean obviously for a person who has been able to sleep 7,8,9 hours when you are constantly waking up….even the small break in a sleep" [S1 PM MD MPR]<br>"I've never felt as low as I have done" [S9 WBF MD HPR]<br>"The injections didn't work. I kind of felt low"[S7 PM MD MPR]<br>"Sometimes I still feel really bad, really low mood because I can't do what I used "[S5 PF HD HPR]<br>"I am awful. I am moody a lot of the time…. it does make me grumpy" [S3 WBF MD MPR]<br>"Not much I can do about it is basically what the guy said" [S4 WBF MD MPR]<br>"There was no other route to go down" [S3 WBF MD MPR]<br>"I went and saw a chiropractor… he gave an x-ray looking for back symptoms …they said it didn't show anything, there was nothing they could do" [S7 PM MD MPR]<br>"I had an MRI scan and that's when they done and my spine was fine I was just annoyed that there was no answers" [S5 PF HD HPR]<br>"It's a ball ache and it's getting on my

		Catastrophising thoughts<br><br>Uncertainty<br><br>Lack of control over pain	nerves" [S4 WBF MD MPR]<br>"It's so frustrating where, when…sighs, yknow things like, picking up my sewing machine I just think well what's up with me?! It's ridiculous" [S9 WBF MD HPR]<br>"You wonder, what if? What if you can't carry on at the same level that you are" [S1 PM MD MPR]<br>"It just makes you think is this the start of something bigger for me?"[S6 PM LD LPR]<br>"They couldn't really give me an explanation really I was thinking then oh what's caused my problem now"[S7 PM MD MPR]<br>"Apprehensive, apprehensive about all those things that I have just spoken about. About what would the impact be in later life…..ahh, that's about it at the moment" [S1 PM MD MPR]<br>"But I'm not sure….having lived with it for this long, I'm not sure" [S3 WBF MD MPR]<br>"It's not improving, it's getting probably a bit more……regular" [S1 PM MD MPR]<br>"I'm just getting to the stage where I'm thinking on the days where it gets really really bad that I'm gone beyond the need of self-help" [S4 WBF MD MPR]
	Subtheme 3.2: Catastrophic nature of pain flare-ups	Catastrophic description: impact on body and mind	"I just…I got up and I had…I got up on the Monday morning ermmm and I felt like a cripple…I felt like I was folding it half, it was hard work to stand up straight"[S8 WBM LD LPR]<br>"It's horrible. It's the worst pain. It makes you cry it's that bad. the pain is ermmm you just can't, you just feel like you've lost control. You can't do anything for yourself you can't like… you can't even go to yknow the loo because you're taking so many steps to get there and every step you take you're like ow, ow, ow and it's that acute ermm it's just horrible. You just…I think you just kind of lose yourself and that's when you start getting depressed like "oh god it's happened again" [S10 PF HD HPR]
	Subtheme 3.3: Threat to self-identity	Threat to self	"I suppose it's hindering my life" [S3 WBF MD MPR]<br>"Obviously it's restricted what I can

			and can't do" [S2 WBM MD HPR]
		Disrupting personal care/daily function	"It's a small thing like bending over the sink to brush your teeth" [S4 WBF MD MPR]<br>"I got out of bed …it took me quite a while to sort of straighten up" [S8 WM LD LPR]<br>"My socks, my shoes, tying my shoes up, I can't bend down" [S9 WBF MD HPR]
		Dependency on family	"It's embarrassing sometimes and degrading because he ..has to help me put my underwear on because I cant bend down to even put my knickers on" [S9 WBF MD HPR]
		Disrupting house chores	"I get the hoover out and hoover, within 10 minutes I'm absolutely shattered and it's hurting my back" [S9 WBF MD HPR]<br>"Mowing the lawn... It's that pushing forward movement I mean it's a push mower.. I mean it takes us about an hour to mow our lawn. And I know that if I sit down after doing that, that I will be in trouble" [S3 WBF MD MPR]<br>"I can't stand at a sink for more than 10 minutes without pain." [S10 PF HD HPR]
		Barrier to recreational activities	"A fair weather golfer…. by the time I played a few holes. I could feel that there was additional pressure on the back" [S1 PM MD MPR]<br>"I can't ride my bike because it's in the leaning forward position and I'll have only gone half a mile but it causes pain in my back" [S3 WBF MD MPR]
		Sleep disruption	"Over the last year it is a genuine struggle if I woke up in the night I struggle to get back to sleep" [S4 WBF MD MPR]<br>"It's okay during the day but at night I'm really suffering"[S5 PF HD HPR]<br>"I am constantly tossing and turning ...."[S1 PM MD MPR]<br>"I sleep on my side but then….i start to ache in my hips and then I have to change sides …. I do wake up because of it and then it takes me about 20 minutes to go back to sleep" [S10 PF HD HPR].

For peer review only

Table: Theme 4 examples

Theme	Subtheme	Code	Quote from participants
**The social and cultural-religious impact of CLBP**	*Subtheme 4.1: Threat to family/friend relationships and social life*	Disrupting family roles/social activity	"If my back goes I can't do nothing, I wouldn't be able to do any housework or…ermmm no bending certainly no bending" [S10 PF HD HPR] "If I'm restricted so is she [wife] in respect of doing things and going out and stuff…."[S2 WBM MD HPR] "Even when I'm driving when we go over a speed bump or a pothole the intense pain that shoots up my back is amazing" [S3 WBF MD MPR]
		Elicit negative family emotions	"When I'm down everybody else feels down in the family" [S9 WBF MD HPR]
		Social disruption, isolation	"You have to stand up in a bar or evening and that just is so painful……., so I tend not to be social" [S10 PF HD HPR].
	Subtheme 4.2: Work impact	Disrupting work	"The pain constraints on work"[S1 PM MD MPR]. "I've also had to raise it with work because of the amount of time I sit by the computer and they've had to get me a special chair" [S10 PF HD HPR] "While my job is actually fully sitting down, any activities where they have asked me to go and do outreach sessions, I have had to restrict them or send other people, which again isn't ideal but other people can deputise as necessary" [S2 WBM MD HPR]
		Burdening colleagues	"I didn't drive.,,.,,I went into the office a few times but then got a lift into the office from colleagues" [S7 PM MD MPR]
		Sickness absence	"I think I only might have had a few days off in the whole time I was there for the pain" [S3 WBF MD MPR] "I'm off work I've had so much time off work"[S9 WBF MD HPR]
		Financial pressures	"I'm in the building trade without a back I can't earn no money. So that goes through your mind as well as that you're not going to get no sick pay or anything" [S6 PM LD LPR] "Still go to work ermmm…because I've got still got bills to pay and I've still for me daughter to support through university and so on and so forth so there's that pressure" [S8 WM

For peer review only

		Changing role	LD LPR]<br>"Initially I suppose I thought…might as well have to look for a different type of job because of the….the physical aspect if you like" [S8 WBM LD LPR]
	Subtheme 4.3: The impact of CLBP on cultural and religious wellbeing	Religious rituals: disrupted sitting to meditate /secluding self<br><br>Disrupting female role within the home/burdening others	"I couldn't do like sit on the floor if it was too hard ..if I sat on the floor for a certain amount of time, then got up, started moving around, my legs would get pins and needles and would reach my back" [S7 PM MD MPR]<br>" [Referring to sitting in the temple] I'll just go when it's quiet yknow, ermmm do my praying and then come back out and then just go downstairs where there's chairs or.. I can just hang around somewhere else and that kind of…I feel secluded sometimes because yknow people tend to like wanna sit upstairs and I have to like go just downstairs" [S10 PF HD HPR]<br>"Because of the pain yknow like I can't help around the house as much as I would like" [S10 PF HD HPR]
	Subtheme 4.4: The response of family, friends and wider community to CLBP	Family: varying response/ support<br><br>Punjabi community: Lack of empathy/ understanding<br><br>Punjabi community: Stigmatized	"My brother……, he was just getting more and more annoyed every time because there was no answers"[S5 PF HD HPR]<br>"People were badgering me, yknow its not getting any better you ought to go …..and see"[S8 WM LD LPR]<br>"Yeah and whenever I did anything they'd say oh watch your back, you'd be carefully about, you don't want that happening again" [S9 WBF MD HPR]<br>"Everyone thinks like "oh her back her back, she just uses that as an excuse now…" [S10 PF HD HPR]<br>"Unfortunately there is always this advice from the community that oh I had a similar thing…no you didn't because you have no idea what the cause of my problem is…"[S1 PM MD MPR]<br>"I believe they're like the doctors that I've experienced. They always assume the worst first yknow" [S10 PF HD HPR]<br>"Oh there comes a fat cow, she's got back, yknow she just can't get up, or oh let's make room for her she's like she can't stand up or just that kind of response. It's not about pain it's about…oh she's too big, she can't do

For peer review only

			it cus she's too big"[S10 PF HD HPR]
	Subtheme 4.5: Recalling family experiences of CLBP	<u>Varying impact of CLBP/coping strategies</u><br><br><u>Varying HCP interactions</u>	"He [Brother] has medication to deal with that on a daily basis"[S4 WBF MD MPR]<br>"He [Dad] felt like a bit of self-dignity went because he was always self-dependent and independent, always doing his own thing"[S5 PF HD HPR]<br>"He [referring to husbands back pain] could sit but he couldn't stand and he couldn't walk…he could just barely walk from the edge of his room to the other side… to get to the kitchen and he would be bent over and he would be crippled". [S3 WBF MD MPR]<br>"He [Dad] has not let it really impact him to be honest" because he still goes to the gym, he still lift weights, just does everything. Before every now and again his back hurts a little bit. He does what he does it he hasn't changed anything"[S6 PM LD LPR]<br>"Well my mum used to have back pain and she always used to say 'oh doctors don't do anything they say they just can't do anything with it'"[S10 PF HD HPR]<br>"My dad suffered from back pain. He had a slipped disk. They said he would never walk again"[S5 PF HD HPR]<br>"She [Husbands physio] explained things; she showed us…..she showed us a back. What you should do with it, what you can do… she gave him exercises to do and how to manage it in the future better" [S3 WBF MD MPR]

For peer review only

Table: Theme 5 examples

Theme	Subtheme	Code	Quote from participants
**Reflecting on HCP interactions, management experience and expectations of future management**	*Subtheme 5.1: Varying quality of therapeutic alliance*	<u>Strong therapeutic alliance</u>	"I felt that it was explained pretty much…completely if you like" [S2 WBM MD HPR]<br>"I left that consultation knowing that I can physically do more than what I thought I could do"[S6 PM LD LPR]<br>"She helped me and showed me how to do it"[S5 PF HD HPR]<br>"I felt they were a lot more like approachable, they were a lot more

		Weak therapeutic alliance	understanding…..I was listened to, that was the biggest thing” [S10 PF HD HPR]<br>“I'm doing exercises, …. which I've been told to do“[S9 WBF MD HPR]<br>“My confidence was improving“[S5 PF HD HPR]<br>“[Referring to chiropractic] Why is that treatment not available on the NHS?” [S10 PF HD HPR]<br>“I get the feeling sometimes that it's maybe a textbook thing to treat back pain” [S4 WBF MD MPR]<br>“I think probably like my GP didn't take me seriously…. I don't think anybody like has the time” [S10 PF HD HPR].<br>“I don't think people understand the severity of the situation“[S4 WBF MD MPR]<br>“With the chiropractor there wasn't much listening ,with the back specialists there was to an extent but it wasn't too involved” [S6 PM LD LPR]<br>“I went back 5 times in total……………..So I kept going back until I got more or less what I had hoped for” [S2 WBM MD HPR]<br>“I got the impression from the Dr that when I went to see the back specialist they would do at least an ultrasound and she didn't” [S8 WM LD LPR]<br>“I was expecting a diagnosis. …. I want him to tell me” [S3 WBF MD MPR]<br>“Yeah, expected no more flare ups ….. within a matter of weeks, 2 weeks if that, I was just sitting down and had another flare up” [S7 PM MD MPR]<br>“She hadn't really done anything to my back, she hadn't really done the tests or anything”[S7 PM MD MPR]<br>“I think I was expecting to be….to kind of have something like that done to me rather than just saying, ‘ok here are the exercises, do them at home” [S5 PF HD HPR]<br>“No sort of a aftercare at all you know put an ice pack on your back that's it “[S6 PM LD LPR]
	Subtheme 5.2: Interpreting the HCP explanation	Adopt caution, Protect spine<br><br>Diagnostic	“[Told by HCP] Be more careful so when you get into bed, don't just jump in”[S7 PM MD MPR]<br>“Just be careful of what I do, just be careful of how I bend, Dr's and physio” [S9 WBF MD HPR]<br>“It could be your movements, it could be

For peer review only

		uncertainty	your muscles, it could be disc, it could be .. anything. Ermmm but obviously…I don't know what's causing my back pain..” [S10 PF HD HPR]<br>“Mixed messages …. oh it could be your stomach, they told me that wear and tear is on my hip and that's causing pain in…. my back” [S7 PM MD MPR]
	Subtheme 5.3: Appraising interventions and ability to control CLBP	Ineffective medication, acupuncture<br><br>Short lasting manual therapies<br><br>Variable impact of injection and exercise therapies	“Some of the pain killers made no difference” [S2 WBM MD HPR]<br>“I went through the acupuncture stage and that didn't work” [S3 WBF MD MPR]<br>“I did go to see a chiropractor as well ermm because at one stage I was just like oh I can't take this anymore…. I had about 8 treatments?......I probably had about 2 or 3 treatments in one week … as soon as it stopped ermm probably about….the first 2 weeks I would say the pain was still there” [S10 PF HD HPR]<br>“They gave me some injections into my facet joints and they didn't help that much... they gave me some more injections but this time they were deeper. So since that time, I've not had any flareups as what they were before but I've had constant pain” [S7 PM MD MPR]<br>“Because I'm exercising its obviously building my muscles and whether that's causing me more pain”[S5 PF HD HPR]<br>“I went to a yoga class three weeks ago and it about killed me but I love it, the next day although there was a dull ache in my back it felt wonderful. I felt like I had really stretched it out” [S4 WBF MD MPR]<br>“My flexibility has increased”[S6 PM LD LPR]<br>“The pain goes away if I do stretching, it goes away and like arching your back over and putting your hands on your buttocks and arching back” [S3 WBF MD MPR]<br>“Exercises … I do them everyday, every morning or night… the underlying problems still there its not going away, its not getting any better” [S9 WBF MD HPR]<br>“When I was walking or running or whatever I was doing the pain was worse than it was before” [S10 PF HD HPR]<br>“My back pain got worse…it was like exercises everyday to do like my back and twisting it” [S7 PM MD MPR]

For peer review only

	Subtheme 5.4: Expectations of future management	<u>Individualised management Mind body approach</u>	"Almost take it case by case, person by person and work out what's best for that person"[S8 WM LD LPR]<br>"The psychological mixed with other things, it's a huge things" [S4 WBF MD MPR]<br>"I think you can help yourself a lot…. By...believing in yourself" [S8 WM LD LPR]
		<u>Effective communication: listen, give time,</u>	"Listening to what I have to say" [S6 PM LD LPR]<br>"They will give me enough time to explain the problem" [S2 WBM MD HPR]<br>"I think, to me its almost just a matter of communicating rather than having a rigid set routine" [S8 WM LD LPR]
		<u>Physical examination Clear explanation and understanding of pain</u>	"Give me some sort of examination of the problem area" [S2 WBM MD HPR]<br>"All I want to know is what's triggering it" [S4 WBF MD MPR]<br>"Explain why and what they're doing in detail that would be understandable, cause sometimes the physio would explain something in their term… like L1 or something, I remember hearing that"[S5 PF HD HPR]<br>"Give you the right advice and guidance"[S3 WBF MD MPR]
		<u>Support and guidance, gain control</u>	"Maybe some follow up consultations to see how have you progressed" [S6 PM LD LPR]<br>"Most importantly that they will give me some advice or treatment, exercise. Something that I can go home and feel that there is progress being made" [S2 WBM MD HPR]
		<u>Increase exercise</u>	"Exercise – yeah well I need to keep doing exercise"[S7 PM MD MPR]<br>"Just some sort of activity I can do that helps it and relieves the pain"[S9 WBF MD HPR]

For peer review only

BMJOPEN Checklist

Title page page 1

Abstract page 2 -3

Strengths and limitations of this study page 3

Introduction page 4-5

Methodology page 6-8

Results page 9 -19

- Table 1: Participant Characteristics page 9
- Table 2: Participant pain, disability and psychosocial risk profile data page 10
- Theme 1: Biomedical back pain beliefs
- Theme 2: Coping with CLBP
- Theme 3: The psychological and emotional dimensions of CLBP
- Theme 4: The social and cultural-religious impact of CLBP
- Theme 5: Reflecting on HCP interactions, management experience and expectations of future management

Discussion page 20 - 25

- Biomedical beliefs
- Coping strategies and transition
- Psychological and emotional dimensions
- Social dimensions
- Therapeutic alliance and a person-centred approach
- Cultural differences

Strengths limitations and implications for future research page 25 -26

Implications for practice and policy page 26

Conclusion page 26

For peer review only

Acknowledgements page 27

Footnotes page 27

- Twitter
- Contributors
- Funding
- Competing interests
- Ethics Approval

References page 28-32

Supplementary files (attached)

- Supplementary File 1
- Supplementary File 2
- Supplementary File 3
- Supplementary File 4
- Supplementary File 5

For peer review only
